# Natural variation in the *ZmPIMT1* promoter enhances seed aging tolerance by regulating PABP2 repair in maize

**DOI:** 10.1093/plcell/koaf217

**Published:** 2025-09-18

**Authors:** Yumin Zhang, Lynnette M A Dirk, Jingliang Zheng, Jiahao Chai, Xianbo Song, Jie Cao, Hao Wang, Yan Liu, Yunjun Liu, Sihan Zhen, Junjie Fu, Guoji Wang, Shixiao Li, Arthur G Hunt, A Bruce Downie, Tianyong Zhao

**Affiliations:** State Key Laboratory of Crop Stress Resistance and High-Efficiency Production, College of Life Sciences, Northwest A&F University, Yangling, Shaanxi 712100, China; Department of Horticulture, Seed Biology, Martin-Gatton College of Agriculture, Food and Environment, University of Kentucky, Lexington, KY 40546, USA; State Key Laboratory of Crop Stress Resistance and High-Efficiency Production, College of Life Sciences, Northwest A&F University, Yangling, Shaanxi 712100, China; State Key Laboratory of Crop Stress Resistance and High-Efficiency Production, College of Life Sciences, Northwest A&F University, Yangling, Shaanxi 712100, China; State Key Laboratory of Crop Stress Resistance and High-Efficiency Production, College of Life Sciences, Northwest A&F University, Yangling, Shaanxi 712100, China; State Key Laboratory of Crop Stress Resistance and High-Efficiency Production, College of Life Sciences, Northwest A&F University, Yangling, Shaanxi 712100, China; State Key Laboratory of Crop Stress Resistance and High-Efficiency Production, College of Life Sciences, Northwest A&F University, Yangling, Shaanxi 712100, China; Chinese Academy of Agricultural Sciences, Institute of Crop Science, Beijing 100081, China; Chinese Academy of Agricultural Sciences, Institute of Crop Science, Beijing 100081, China; Chinese Academy of Agricultural Sciences, Institute of Crop Science, Beijing 100081, China; Chinese Academy of Agricultural Sciences, Institute of Crop Science, Beijing 100081, China; Research Center, Gansu Wugu Seed Co., Ltd, Lanzhou, Gansu province 730070, China; Research Center, Gansu Wugu Seed Co., Ltd, Lanzhou, Gansu province 730070, China; Department of Plant and Soil Sciences, Martin-Gatton College of Agriculture, Food and Environment, University of Kentucky, Lexington, KY 40546, USA; Department of Horticulture, Seed Biology, Martin-Gatton College of Agriculture, Food and Environment, University of Kentucky, Lexington, KY 40546, USA; State Key Laboratory of Crop Stress Resistance and High-Efficiency Production, College of Life Sciences, Northwest A&F University, Yangling, Shaanxi 712100, China

## Abstract

PROTEIN L-ISOASPARTYL O-METHYLTRANSFERASE (PIMT) promotes seed vigor by repairing damaged proteins. However, whether PIMT variants have arisen during maize (*Zea mays*) domestication remains unknown. Here, we found 2 variants in the *ZmPIMT1* promoter. The *ZmPIMT1*  ^Hap C7–2^ promoter exhibited stronger activity than the *ZmPIMT1*  ^Hap Z58^ promoter. Maize inbred lines carrying the *ZmPIMT1*  ^Hap C7–2^ promoter had greater seed vigor than *ZmPIMT1*  ^Hap Z58^ lines in a population of Zhengdan 958 recombinant inbred lines (RILs) and a maize inbred population. By characterizing the maize *zmpimt1* knockdown mutant, *ZmPIMT1*-overexpressing maize and *Arabidopsis thaliana* heterologous *ZmPIMT1* overexpression lines, we demonstrated that ZmPIMT1 positively regulates seed vigor. Co-IP and LC-MS/MS assays showed that ZmPIMT1 interacts with and repairs damaged POLY(A) BINDING PROTEIN2 (PABP2). ZmPIMT1 stabilizes PABP2 RNA-binding activity and regulates the stability and translation efficiency of the mRNA during maize seed germination. Disruption of *PABP2* decreases seed vigor in *Arabidopsis thaliana*. Furthermore, the *F*-statistics (Fixation index; *F*_ST_) and nucleotide diversity (*θ*π) ratio between teosinte and maize lines showed that *ZmPIMT1* likely has not undergone selection during maize domestication. Our findings unveil a molecular mechanism in which ZmPIMT1 regulates seed vigor in maize and highlight a potential application of the advantageous *ZmPIMT1* haplotype for breeding new varieties with increased seed vigor.

## Teaser

A natural variation in the promoter of a protein damage repair gene enhances seed vigor and longevity, providing a target for breeding improvement.

## Introduction

Seed longevity denotes the viability of orthodox seeds during long-term storage and is crucial for ensuring food and nutritional security. Several proteins that regulate seed viability have been identified, such as those that repair damage (PROTEIN L-ISOASPARTYL O-METHYLTRANSFERASE (PIMT), METHIONINE SULFOXIDE REDUCTASEs (MSRs)) (Hazra et al. 2022; Zhang et al. 2023), and those that protect proteins (HEAT SHOCK PROTEINs (HSPs), LATE EMBRYOGENESIS ABUNDANT PROTEINs (LEAPs)) (Ma et al. 2019); additionally various sugars (raffinose) (Li et al. 2017) are involved in seed longevity as are antioxidants (tocopherols (Vitamin E)) (Sattler et al. 2004).

PIMT functions by repairing L-isoAsp residues that spontaneously and non-enzymatically arise in proteins, converting them to L-Asp. PIMTs (PROTEIN CARBOXY METHYLTRANSFERASE (PCMT) in micro-organisms) are present in most kingdoms of life, from archaea to humans (*Homo sapiens*) (O'Connor 2006). Plants and some of the archaea possess more than 1 PIMT paralog (Kamble and Majee 2020). This repair pathway is particularly active in seeds as part of the natural protection and repair mechanism (Fernandez-Marin et al. 2013; Sano et al. 2016; Dirk and Downie 2018). Only some PIMT enzymes can methylate D-Asp (Lee et al. 2012).

The expression patterns and subcellular localizations of PIMTs varies in plants. Usually, PIMT1 is expressed in all organs and tissues, whereas PIMT2 is mainly expressed during seed dehydration and maturation (Zhang et al. 2023). PIMT2 possesses a unique N-terminal extension (about 50 amino acids containing a signal peptide) compared with PIMT1 that leads to different subcellular localizations of the PIMTs. AtPIMT2 and ZmPIMT2 are localized in the cytoplasm, chloroplasts, and mitochondria (Dinkins et al. 2008; Zhang et al. 2023). OsPIMT2 and CaPIMT2 are localized in the nucleus (Verma et al. 2013; Wei et al. 2015). PIMT1 is usually localized in the cytosol (Verma et al. 2013; Wei et al. 2015).

Transcription factors involved in the ABA pathway regulate the expression of *PIMTs*. VIVIPAROUS1 (VP1) regulates *ZmPIMT2* and promotes the accumulation of *ZmPIMT2* during seed maturation in maize (Zhang et al. 2023). In *Arabidopsis*, the expression of *AtPIMT1* is directly controlled by ABSCISIC ACID INSENSITIVE4 (AtABI4) (Kamble et al. 2022), and the expression of *AtPIMT2* is regulated by AtABI3 (Tian et al. 2020). Despite studies on the expression regulation and subcellular localization of PIMTs, whether natural variants of *ZmPIMT1* exist in maize populations and whether alleles have undergone selection during maize domestication remains unknown.

Several target proteins preferentially interact with and are repaired by PIMT. In *Arabidopsis*, PLANT RNA HELICASE75 (PRH75), SUPEROXIDE DISMUTASE (SOD), and CATALASE enzymes (CAT) are the targets of AtPIMT (Nayak et al. 2013; Ghosh et al. 2020). Maize 3-METHYLCROTONYL COA CARBOXYLASE (ZmMCC) is a substrate of ZmPIMT2 (Zhang et al. 2023). In humans, tumor suppressor p53, CALMODULIN, and TUBULIN have been identified as preferred target proteins of PIMT (O'Connor and O'Connor 1998; Lee et al. 2012). It is well known that PIMT regulates seed vigor (defined here as enhanced viability) in plants producing orthodox seeds (Oge et al. 2008; Verma et al. 2010; Verma et al. 2013; Wei et al. 2015; Petla et al. 2016; Zhang et al. 2023)n on the role and regulation of PIMTs in recalcitrant seed-producing plants are still limited.

RNA is easily degraded during environmental stress. Dry seeds contain various stored mRNAs that are believed to be required for protein synthesis during the early stages of seed germination (Nakabayashi et al. 2005; Howell et al. 2009). Poly(A)-binding proteins (PABPs) bind RNA and regulate mRNA translation and stability (Eliseeva et al. 2013). Protein synthesis is crucial for the initiation of seed germination (Bai et al. 2020). However, little is known about the repair of damaged PABPs and its effects on seed vigor or longevity.

This report demonstrates that natural variation exists in the *ZmPIMT1* promoter in maize populations. The *ZmPIMT1*  ^Hap C7–2^ promoter had higher activity than *pZmPIMT1*  ^Hap Z58^. In addition, the *ZmPIMT1* expression level and the seed vigor of the *ZmPIMT1*  ^Hap C7–2^ type seeds was greater than that of *ZmPIMT1*  ^Hap Z58^ type seeds. We also find that ZmPIMT1 interacts with the maize ortholog of PABP2 (ZmPABP2) and that it acts to preserve or stabilize the activity of the protein by converting isoaspartate residues to aspartate residues in the protein. These results suggest that natural variation in ZmPIMT1 expression regulates translation by modulating PABP2, maintaining the protein's structure and activity during seed maturation and germination.

## Results

### *ZmPIMT1*  ^Hap C7–2^, a natural variant of *ZmPIMT1* promoter, exhibits increased activity and correlates with higher seed vigor

**Figure 1.**
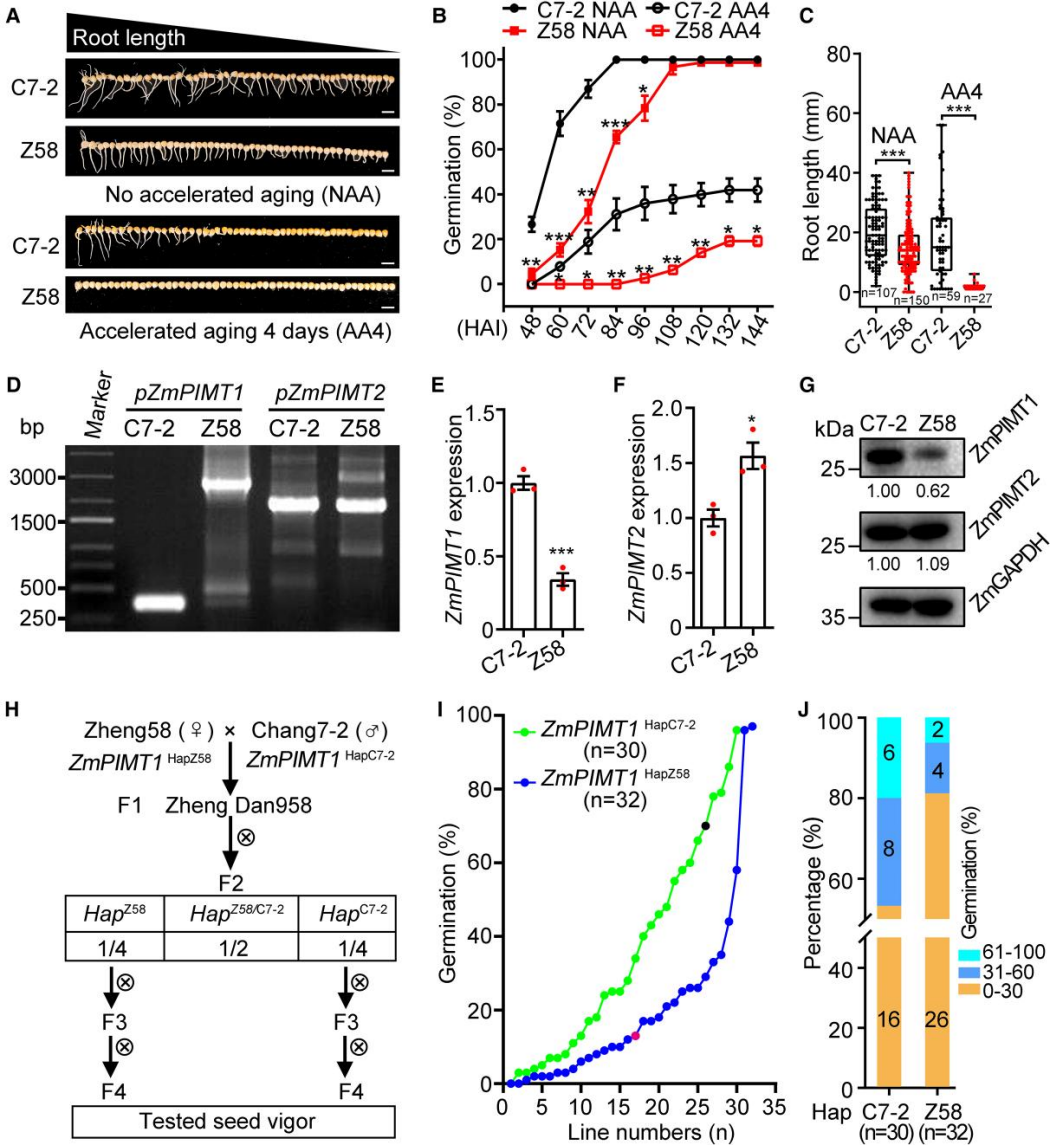
Natural variation of the *ZmPIMT1* promoter is highly relevant to maize seed vigor in the Zhengdan 958 RIL population. **A)** Photographs of 1 replicate of Chang7-2 (C7-2) and Zheng58 (Z58) seedlings after imbibition for 144 h without accelerated aging (NAA) treatment or with accelerated aging treatment for 4 d (AA4). The scale bar represents 2 cm. **B)** Comparison of seed germination percentages between Chang7-2 (C7-2) and Zheng58 (Z58) untreated (NAA) or AA-treated seeds (AA4). Seeds were imbibed at 25 °C in the dark, and the completion of germination was monitored every 12 h from 48 to 144 h. There were 3 biological replicates per treatment, each containing 50 seeds. Results are expressed as the mean ± SE. Asterisks denote significance relative to Chang7-2 within the same AA treatment (Student's *t*-test; * *P* < 0.05; ** *P* < 0.01; *** *P* < 0.001). **C)** Comparison of root lengths of seedlings generated in (A). The root length distribution of each group is displayed as a box plot. In the box plot, the box's lower and upper edges represent the first and third quartiles, respectively, with an internal line marking the median. The whiskers extend from the quartiles to the dataset's minimum and maximum values. Each dot, representing a seedling root length, is indicated. The number of roots measured are below each box. Statistical significance was determined using a 2-sided *t*-test (***P < 0.001). **D)** Amplicon size identified variation in the promoter of *ZmPIMT1* but not in that of *ZmPIMT2* in Chang7-2 and Zheng58 inbred lines. Specific primers for each gene (F4 and R4 for *ZmPIMT1* and F5 and R5 for *ZmPIMT2*; Supplementary Table S1) were used. **E)** Real-time RT-PCR analysis of the *ZmPIMT1* expression in the embryo of Chang7-2 and Zheng58 inbred lines. The expression of *ZmPIMT1* was normalized to *ZmGAPDH* expression. Data are means ± SE (n = 3) (***P < 0.001). **F)** Real-time RT-PCR analysis of the *ZmPIMT2* expression in the embryo of Chang7-2 and Zheng58 inbred lines. The expression of *ZmPIMT2* was normalized to *ZmGAPDH* expression. Data are means ± SE (n = 3) (*P < 0.05). **G)** Western blot analysis of the ZmPIMT1 and ZmPIMT2 protein accumulation in the 12 HAI embryos of Chang7-2 and Zheng58 inbred lines. The ZmGAPDH protein was used for equal protein loading control and to calculate the ZmPIMT1/ZmGAPDH or ZmPIMT 2/ZmGAPDH signal intensity ratios (given below each of the PIMT blots). **H)** The diagram indicates the process of obtaining *ZmPIMT1*  ^Hap C7–2^ and *ZmPIMT1*  ^Hap Z58^ RIL population from the parental maize inbred lines Zheng58 and Chang7-2. **I)** Comparison of seed germination percentages between the *ZmPIMT1*  ^Hap C7–2^ and *ZmPIMT1*  ^Hap Z58^ RIL population seeds treated with accelerated aging (AA) for 7.5 d. After AA treatment, the seeds were then germinated at 25 °C in the dark and the completion of germination was monitored at 120 h. For germination, there were 100 seeds tested for each RIL population (29 lines denoted with green dots and line for *ZmPIMT1*  ^Hap C7–2^; 31 lines denoted with blue dots and line for *ZmPIMT1*  ^Hap Z58^) and the respective parental line (black dot denote Chang7-2; magenta dot denote Zheng58). **J)** The distributions of the parental lines and all of the *ZmPIMT1*  ^Hap C7–2^ and *ZmPIMT1*  ^Hap Z58^ RIL population 144 HAI seed germination percentages after AA 7.5 d.

Stored seed from the same harvest of Zheng58 and Chang7-2, the 2 parental lines of the maize hybrid Zhengdan 958, the most popular corn variety in China based on the area planted (Liu et al. 2013), exhibited significant differences in final seed germination percentage under both optimal and accelerated aging (AA) conditions. Without AA treatment (NAA), Chang7-2 seeds showed a significantly higher germination percentage than Zheng58 from 48 to 96 h after imbibition (HAI), though both lines reached full germination by 120 HAI (Fig. 1, A and B). After a 4-d AA treatment (AA4), completion of germination was significantly reduced in both lines, but Chang7-2 still outperformed Zheng58, particularly after 60 HAI (Fig. 1, A and B). Additionally, Chang7-2 seedlings developed longer roots following AA treatment (Fig. 1C).

Given the role of PROTEIN L-ISOASPARTYL O-METHYLTRANSFERASE (PIMT) in seed vigor, we examined the PIMT-encoding genes in these lines. There are 2 PIMTs (ZmPIMT1 and ZmPIMT2) in maize (Zhang et al. 2023). While *ZmPIMT2* showed no significant variation, the *ZmPIMT1* promoter region differed markedly between Chang7-2 and Zheng58 (Fig. 1D). The levels of *ZmPIMT1* mRNA transcripts in Chang7-2 embryos were significantly higher than in Zheng58 embryos (Fig. 1E), Quantitative analysis revealed higher *ZmPIMT1* mRNA and protein levels in Chang7-2 embryos compared to Zheng58 (Fig. 1, E and G), whereas *ZmPIMT2* expression was lower in Chang7-2 (Fig. 1F).

To further investigate the impact of *ZmPIMT1* promoter variation, we developed a Zhengdan 958 recombinant inbred line (RIL) population (Fig. 1H). A total population of 31 *ZmPIMT1*  ^Hap Z58^ RILs (Supplementary Fig. S1A) and 29 *ZmPIMT1*  ^Hap C7–2^ RILs were confirmed by PCR (Supplementary Fig. S1B). The seed morphology of the Zhengdan 958 RIL population differed from that of either parent, Zheng58 or Chang7-2 (Supplementary Fig. S2). Lines carrying *ZmPIMT1*  ^Hap C7–2^ exhibited superior seed vigor compared to those with the *ZmPIMT1*  ^Hap Z58^ promoter (Fig. 1I). Notably, 81% of *ZmPIMT1*  ^Hap Z58^ lines had germination percentages below 30%, whereas only 53% of *ZmPIMT1*  ^Hap C7–2^ lines fell into this category after 7.5-d AA treatment (Fig. 1J). Conversely, 20% of *ZmPIMT1*  ^Hap C7–2^ lines achieved germination percentages exceeding 60%, compared to just 6.25% of *ZmPIMT1*  ^Hap Z58^ lines (Fig. 1J). These results underscore the critical role of *ZmPIMT1* promoter variation on seed vigor.

**Figure 2.**
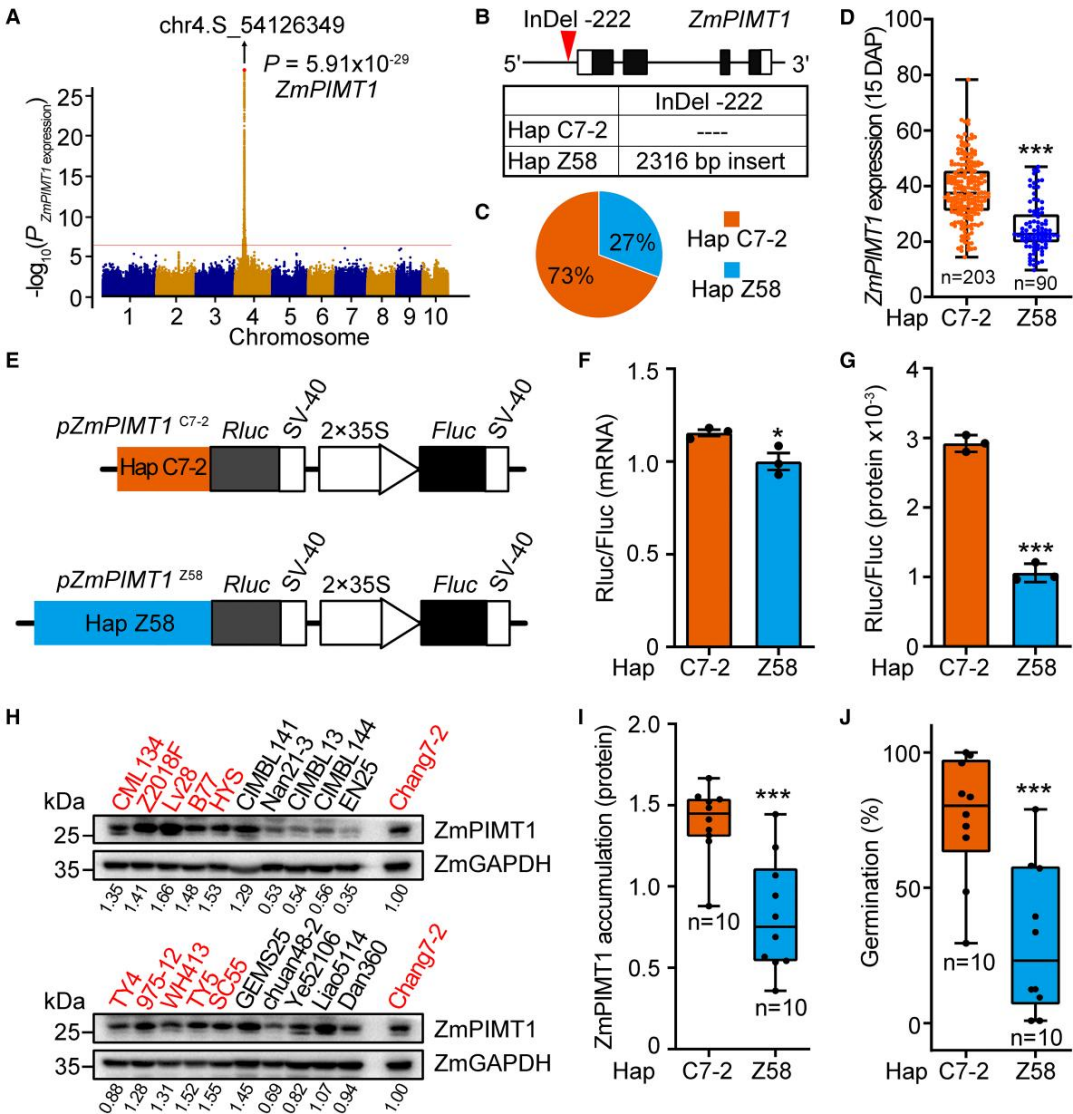
The *ZmPIMT1*  ^Hap C7–2^ lines exhibits increased *ZmPIMT1* promoter activity and seed vigor compared with the *ZmPIMT1*  ^Hap Z58^ lines. **A)** eGWAS analysis of Z*mPIMT1* expression of 15 d after pollination (DAP) embryos using mixed linear model (MLM) method. The red dot line represents the threshold (*P* = 3.37 × 10^−7^) derived from the Bonferroni correction method, and the red dot signify significant SNP sites corresponding to *ZmPIMT1* itself. **B)** Haplotype analysis of the promoter region of *ZmPIMT1* in maize inbred lines. Exons are shown as black boxes, and UTRs are shown as white boxes. The InDel insertion site was shown with the red triangle in the graphic representation (sequence details in Supplementary Fig. S4). Hap, Haplotype. **C)** Frequency distribution of 2 haplotypes of *ZmPIMT1*  ^Hap C7–2^ and *ZmPIMT1*  ^Hap Z58^ among 301 maize inbred lines, as determined by amplicon size with primers F4 and R4 (as in Fig. 1D and Supplementary Fig. S3). Hap, Haplotype. **D)** Comparison of the *ZmPIMT1* expression at 15 DAP embryos between *ZmPIMT1*  ^Hap C7–2^ and *ZmPIMT1*  ^Hap Z58^ haplotypes by box plot. In the box plot, the box's lower and upper edges represent the first and third quartiles, respectively, with an internal line marking the median. The whiskers extend from the quartiles to the dataset's minimum and maximum values. Each dot represents the *ZmPIMT1* expression at 15 DAP of an inbred line. Statistical significance was determined using a 2-sided *t*-test (***P < 0.001). Hap, Haplotype. **E)** Depiction of the 2 types of *ZmPIMT1* promoter, *ZmPIMT1*  ^Hap C7–2^ or *ZmPIMT1*  ^Hap Z58^, upstream of the *Rluc*, and 2 × 35S promoter upstream of *Fluc*. Fluc, Firefly luciferase. Rluc, Renilla luciferase. **F)** Comparison of the *Rluc* mRNA expression in maize protoplast cells transformed with *Rluc* expression vectors in which *Rluc* is driven by either *ZmPIMT1*  ^Hap C7–2^ or by *ZmPIMT1*  ^Hap Z58^. Data are presented as means ± SE (n = 3), and statistical significance (*P < 0.05) was determined using a Student's *t*-test. Hap, Haplotype. Fluc/Rluc, Firefly luciferase/Renilla luciferase. **G)** Comparison of the Rluc activity in maize protoplast cells transformed with *Rluc* expression vectors in which *Rluc* is driven by either *ZmPIMT1*  ^Hap C7–2^ or by *ZmPIMT1*  ^Hap Z58^.The Rluc activity of *ZmPIMT1*  ^Hap C7–2^ is significantly higher than *ZmPIMT1*  ^Hap Z58^. Data are means ± SE (n = 3), and statistical significance (***P < 0.001) determined using a Student's *t*-test. Hap, Haplotype. Fluc/Rluc, Firefly luciferase/Renilla luciferase. **H)** Western blot detecting protein accumulation of ZmPIMT1 in the embryos of 20 inbred lines at 12 h after imbibition (HAI) (both top and bottom figures). The ZmGAPDH protein was used for equal protein loading control (bottom panel in each figure). The red and black fonts denote the inbred line with the C7-2 and Z58 haplotypes, respectively. **I)** Grayscale analysis of the ZmPIMT1 protein abundance between the 2 haplotypes. The amount of ZmPIMT1 in Chang7-2 was used to normalize the results across blots. In the box plot, the box's lower and upper edges represent the first and third quartiles, respectively, with an internal line marking the median. The whiskers extend from the quartiles to the dataset's minimum and maximum values. Each dot represents the calculated ZmPIMT1 volumes from 1 Western blot. Statistical significance was determined using a 2-sided *t*-test (***P < 0.001). Hap, Haplotype. **J)** Comparison of final seed germination percentage at 7 d after imbibition (DAI) between Hap C7-2 and Hap Z58 seeds treated for 3 d with accelerated aging. In the box plot, the box's lower and upper edges represent the first and third quartiles, respectively, with an internal line marking the median. The whiskers extend from the quartiles to the dataset's minimum and maximum values. Each dot represents an inbred line. Statistical significance was determined using a 2-sided *t*-test (***P < 0.001). Hap, Haplotype.

To further explore the variation of *ZmPIMT1* in maize inbred populations, the expression of *ZmPIMT1* at 15 d after pollination (DAP) in 368 maize inbred lines (Fu et al. 2013) was used to perform an expression genome-wide association analysis (eGWAS). This analysis yielded a significant SNP (Chr4.S-53962085, B73 RefGen V2) within the *ZmPIMT1* gene region (Zm00001d049966) (Fig. 2A). To further explore the variation type and exact site of *ZmPIMT1* variation, we designed specific primers capable of amplifying a segment of the *ZmPIMT1* promotor region. We used PCR to analyze gDNA from 301 maize inbred lines (those lines coming from the 368 inbred lines and grown in Yangling), teosinte, Zheng58 and Chang7-2 (Supplementary Fig. S3). This analysis yielded as many as 4 identifiable polymorphisms (color-coded in Supplementary Fig. S3). A plurality showed the shorter-(365 bp), Chang7-2-like PCR product, while many other lines showed the longer-(2682 bp), Zheng58-like amplicon (Fig. 1D, Supplementary Fig. S3); these latter lines were further distinguished by the number and sizes of the longer PCR products. Sequencing results identified a 2,316 bp insertion located 222 bp upstream of the start codon in the *ZmPIMT1* gene of the Zheng58 type and 15 other polymorphisms (2 of which are in the 5' UTR) (Fig. 2B, Supplementary Fig. S4). Among the inbred lines investigated, a total of 221 (73%), including Chang7-2, had the shorter *ZmPIMT1* promoter; this haplotype was named *ZmPIMT1*  ^Hap C7–2^. In contrast, 80 inbred lines (27%), including Zheng58, had the longer *ZmPIMT1* promoter; this haplotype was named *ZmPIMT1*  ^Hap Z58^ (Fig. 2C).

These polymorphisms in the *ZmPIMT1* promoter suggest an explanation for the differences in *ZmPIMT1* expression noted in Fig. 1. To test this, the expression of the *ZmPIMT1* gene was measured in the different inbred lines characterized in Supplementary Fig. S3. The expression of *ZmPIMT1* in *ZmPIMT1*  ^Hap C7–2^ was significantly higher than in *ZmPIMT1*  ^Hap Z58^ at 15 DAP (Fig. 2D). To confirm that these differences were due to the promoters, the activities of the *ZmPIMT1*  ^Hap C7–2^ and *ZmPIMT1*  ^Hap Z58^ promoters were measured in maize protoplasts using a dual luciferase assay (Fig. 2E). The results show that the *ZmPIMT1*  ^Hap C7–2^ promoter activity was significantly higher than *ZmPIMT1*  ^Hap Z58^ (Fig. 2, F and G). These results indicated that sequence differences in the *ZmPIMT1* promoter affected its activity, with the *ZmPIMT1*  ^Hap C7–2^ promoter showing stronger activity than the *ZmPIMT1*  ^Hap Z58^ promoter.

To investigate whether the reduction of *ZmPIMT1* transcript abundance due to promoter variation influenced ZmPIMT1 abundance and maize seed vigor in maize inbred lines, ZmPIMT1 protein accumulation of 10 maize inbred lines carrying *ZmPIMT1*  ^Hap C7–2^ and 10 lines carrying *ZmPIMT1*  ^Hap Z58^ (PCR identified as in Supplementary Fig. S5) was measured at 12 HAI by Western blot analysis (Fig. 2H). The results showed that ZmPIMT1 protein accumulation was significantly higher in the *ZmPIMT1*  ^Hap C7–2^ lines than in the *ZmPIMT1*  ^Hap Z58^ lines (Fig. 2I). The seed vigor of these 20 inbred lines was compared after 3 d of AA treatment. Those lines in which ZmPIMT1 was more abundant (*ZmPIMT1*  ^Hap C7–2^) had greater seed vigor than lines with lower ZmPIMT1 protein levels (*ZmPIMT1*  ^Hap Z58^) (Fig. 2J).

**Figure 3.**
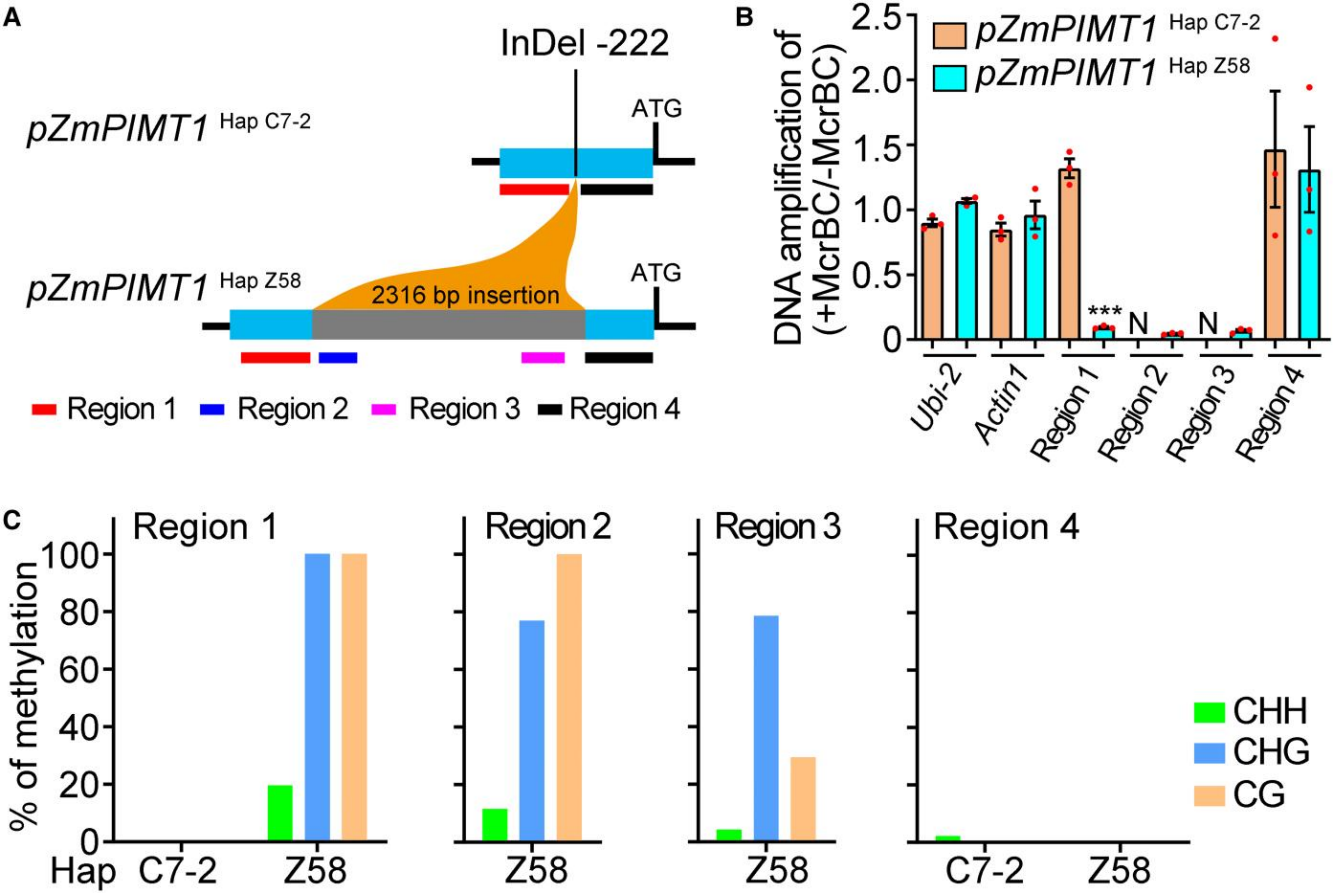
Comparison of DNA methylation status in the *ZmPIMT1* promoter between *pZmPIMT1*  ^Hap C7–2^ and *pZmPIMT1*  ^Hap Z58^. **A)** The diagram of regions 1, 2, 3, and 4 in the *ZmPIMT1* promoter of different haplotypes of *ZmPIMT1*. InDel −222 indicates the 2316-bp insertion site in the *ZmPIMT1* promoter. **B)** Comparison of DNA methylation status of the 4 regions (R1–R4) in the *ZmPIMT1* promoter between haplotypes with McrBC-qPCR analysis. Data are means ± SE (n = 3), and statistical significance (***P < 0.001) determined using a Student's *t*-test. The DNA methylation state of the maize *Ubi-2* and *Actin1* were tested in parallel as negative controls. N denotes that the *pZmPIMT1*  ^Hap C7–2^ will not have the binding site for the primers. **C)** Comparison of the CHH (H = A, C, or T), CHG, and CG methylation percentages of Regions 1, 2, 3, and 4 by bisulphite sequencing between *pZmPIMT1*  ^Hap C7–2^ and *pZmPIMT1*  ^Hap Z58^. Eight clones of each genotype were sequenced. Hap, Haplotype.

To investigate how the 2316-bp insertion in the promoter of *ZmPIMT1* correlates with the reduced *ZmPIMT1* expression, we examined the difference of the DNA methylation status of the promoter region of *ZmPIMT1* between the *ZmPIMT1*  ^Hap C7–2^ and *ZmPIMT1*  ^Hap Z58^ haplotypes (Fig. 3A). Of the 2 regions (1 and 4) in common between the 2 haplotypes, McrBC-qPCR analysis results revealed that region 1 was methylated only in the Zheng58 haplotype (Fig. 3B). Both *pZmPIMT1*  ^Hap Z58^ specific regions (2 and 3) were also methylated, as determined by the same analysis (Fig. 3B). By the locus-specific bisulfite sequencing (BS-seq) method, regions 1, 2 and 3 of *pZmPIMT1*  ^Hap Z58^ had relatively large percentages of cytosine methylation of CHG (H = A, C, or T) and CG types (dependent on the context, specifically CHG and CG) and a small percentage with the CHH context (Fig. 3C). In region 4, that same analysis showed barely detectable CHH methylation percentage for only the *pZmPIMT1*  ^Hap C7–2^(Fig. 3C). These data demonstrate that the 2316-bp insertion likely represses the *ZmPIMT1* promoter activity of the Zheng58 haplotype through, at least partially, DNA methylation.

To better understand the temporal expression pattern, protein accumulation dynamics, and the correlation between transcript abundance and protein levels, specific primers were designed and ZmPIMT1 antibodies were employed. The accumulation of both the mRNA and ZmPIMT1 protein at 25, 30 and 40 DAP were higher than that of 20 DAP during W22 seed maturation (Supplementary Fig. S6, A and B). During seed germination, *ZmPIMT1* mRNA amounts were similar at 0, 12, and 24 HAI, and dramatically increased at and after 36 HAI (Supplementary Fig. S6C). ZmPIMT1 protein levels were 2-fold higher as soon as 12 HAI than at 0 HAI (Supplementary Fig. S6D). Since transcript amounts had not changed at 12 HAI, this result implicates regulation at the level of translation or protein stability within that timeframe.

**Figure 4.**
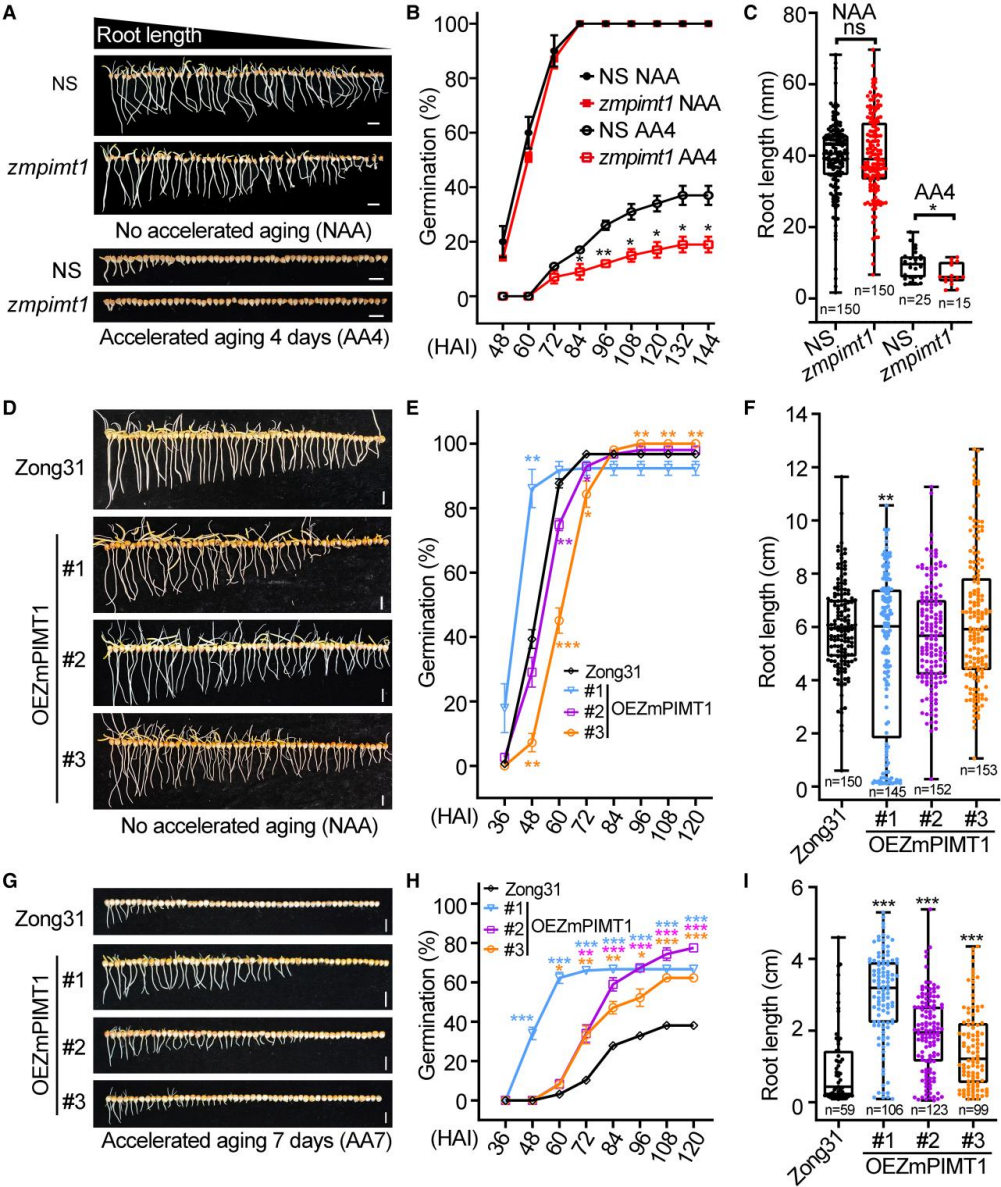
ZmPIMT1 positively regulates maize seed vigor. **A)** Photographs of 1 replicate of NS and *zmpimt1* seeds/seedlings after imbibition for 120 h without accelerated aging of the seed (NAA; top panel) and 144 HAI following 4 d of accelerated aging (AA4; bottom panel). The white scale bar represents 2 cm. **B)** Comparison of seed germination percentages between NS and *zmpimt1* untreated (NAA) or AA-treated (AA4) seeds. Seeds were imbibed at 25 °C in the dark, and the completion of germination was monitored every 12 h from 48 to 144 h. There were 3 biological replicates per treatment, each containing 50 seeds. Results are expressed as the mean ± SE. The asterisks denote significance relative to NS within the same AA treatment (Student's *t*-test; *P < 0.05 and **P < 0.01). **C)** Comparison of root length of seedlings generated in (B). The root length distribution of each group is displayed as a box plot. Each dot represents 1 biological replicate. The number of biological replicates is indicated. Statistical significance was determined using a 2-sided *t*-test (**P < 0.01). **D** and **G)** Photographs of 1 replicate (n = 3) of Zong31 and *ZmPIMT1*-transformed seed/seedlings after imbibition for 120 h without prior accelerated aging (NAA) treatment **(D)** or following 7 d of accelerated aging (AA7) treatment **(G)**. The white scale bar represents 2 cm. **E** and **H)** Comparison of seed germination percentages between Zong31 and *ZmPIMT1*-overexpressing seeds that were untreated with accelerated aging **(E)** or following AA7 treatment **(H)**. Seeds were imbibed at 25 °C in the dark, and the completion of germination was monitored every 12 h from 36 to 120 h. There were 3 biological replicates per treatment, each containing 50 seeds. Results are expressed as the mean ± SE. The asterisks denote significance relative to Zong31 within the same treatment (Student's *t*-test; *P < 0.05, **P < 0.01 and ***P < 0.001). **F** and **I)** Comparison of root length of seedlings at the final 120 HAI generated in **(E)** and **(G)**, respectively. The root length distribution of each group is displayed as a box plot. Each dot represents 1 biological replicate. Statistical significance was determined using Student's *t*-test (**P < 0.01 and ***P < 0.001).

The role of ZmPIMT1 in seed vigor was further studied using a *zmpimt1* knockdown mutant line (Supplementary Fig. S7A). The homozygous mutant and its respective null segregant (NS) line were selected from the segregation of the self-fertilized heterozygous mutant. The insertion of *Mu* in *zmpimt1* is illustrated (Supplementary Fig. S7A) and was confirmed by PCR (Supplementary Fig. S7B) and amplicon sequencing (Supplementary Fig. S7A, Supplementary Fig. S8). After 12 HAI, the levels of *ZmPIMT1* mRNA in the *zmpimt1* mutant were approximately 30% lower than in the NS control (Supplementary Fig. S7C); whereas the *ZmPIMT2* transcript was unaltered (Supplementary Fig. S7E). ZmPIMT1 protein accumulation (as a ratio to ZmGAPDH) in the *zmpimt1* mutant was 37% less than that seen with the NS control (Supplementary Fig. S7D); whereas the ZmPIMT2 was essentially unchanged (Supplementary Fig. S7F). The seed vigor of the NS and *zmpimt1* mutant was tested using accelerated aging (AA) treatment (Fig. 4A). There was no significant difference in the germination percentage between unaged seeds of the *zmpimt1* mutant and the NS control line (Fig. 4B). However, with seeds aged for 4 d (AA4), beginning at 84 HAI, the germination percentage of *zmpimt1* mutant seeds was significantly less than that of the NS control line (Fig. 4B). In addition, the root lengths of *zmpimt1* seedlings were significantly shorter than NS control lines after AA4 treatment (Fig. 4C).

To determine whether overexpression of the *ZmPIMT1* gene in maize could enhance seed aging tolerance, a *ZmPIMT1*-overexpressing vector was constructed (Supplementary Fig. S9A) and transformed into the maize Zong31 inbred line. Three independent *ZmPIMT1*-overexpressing maize lines (OEZmPIMT1 #1, #2, and #3) were obtained and confirmed by PCR (Supplementary Fig. S9, A and B). The *ZmPIMT1* transcript abundance in 24 h-imbibed embryos of the 3 transgenic lines were significantly greater than those in similarly-treated non-transformed Zong31 plants (Supplementary Fig. S9C). Western blot analysis confirmed that the ZmPIMT1 protein accumulation in 24 h-imbibed, mature embryos of the 3 transgenic lines was greater than that of the non-transgenic Zong31 control (Supplementary Fig. S9D).

To further examine the connection between ZmPIMT1 and seed vigor, the germination of *ZmPIMT1* overexpressing lines and the non-transgenic Zong31 control line was tested without accelerated aging (NAA) or with (AA) aging treatment (Fig. 4, D and G, respectively). When seeds were germinated under optimal control conditions (NAA), the germination percentage of the OEZmPIMT1-#3 transgenic line at 48 and 60 h after imbibition (HAI), was significantly lower than that of the Zong31 control line; whereas the germination percentage of the OEZmPIMT1-#1 transgenic line at 48 HAI was greater than Zong31 control line (Fig. 4E). At and after 96 HAI, the germination percentage of the *OEZmPIMT1-#*3 line was greater than that of the Zong31 control (Fig. 4E). At 120 HAI, the root length of unaged transgenic line #1 was significantly shorter than that of the Zong31 control (Fig. 4F). There was no difference in the root length between lines #2, #3, and the Zong31 control line (Fig. 4F), despite the delayed completion of germination of seeds of #3 relative to Zong31 (Fig. 4E). With 7 d AA treatment, the germination percentage of all 3 transgenic lines was significantly greater than that of the Zong31 line at and after 72 HAI; and OEZmPIMT1-#1 and #3 had larger germination percentages relative to that of Zong31 as early as 48 and 60 h, respectively (Fig. 4H). The root lengths of all 3 transgenic lines with AA treatment were significantly greater than that of the Zong31 control line at 120 HAI (Fig. 4I).

The seed aging tolerance of *ZmPIMT1*-overexpressing *Arabidopsis* seeds was also investigated. The germination percentage of *ZmPIMT1*-overexpressing lines was significantly greater than that of WT when the seeds had been stored at room temperature for 1 year or after AA treatment (Supplementary Fig. S10).

### ZmPIMT1 interacts with PABP2

The results shown in Figs. 1 to 3 implicate the activity of ZmPIMT1, and in turn of 1 or more of its substrates (or targets), in the determination of seed vigor. To further explore this, an unbiased co-immunoprecipitation and LC-MS/MS analysis was conducted to identify possible ZmPIMT1 targets. Confirming that the anti-ZmPIMT1 antibody was specific, the Co-IP assay identified the ZmPIMT1 protein. In contrast, the ZmPIMT1 protein was not recovered in controls using an anti-ZmPIMT2 antibody (line 68 in Supplementary Table S2, Supplementary Fig. S11).

**Figure 5.**
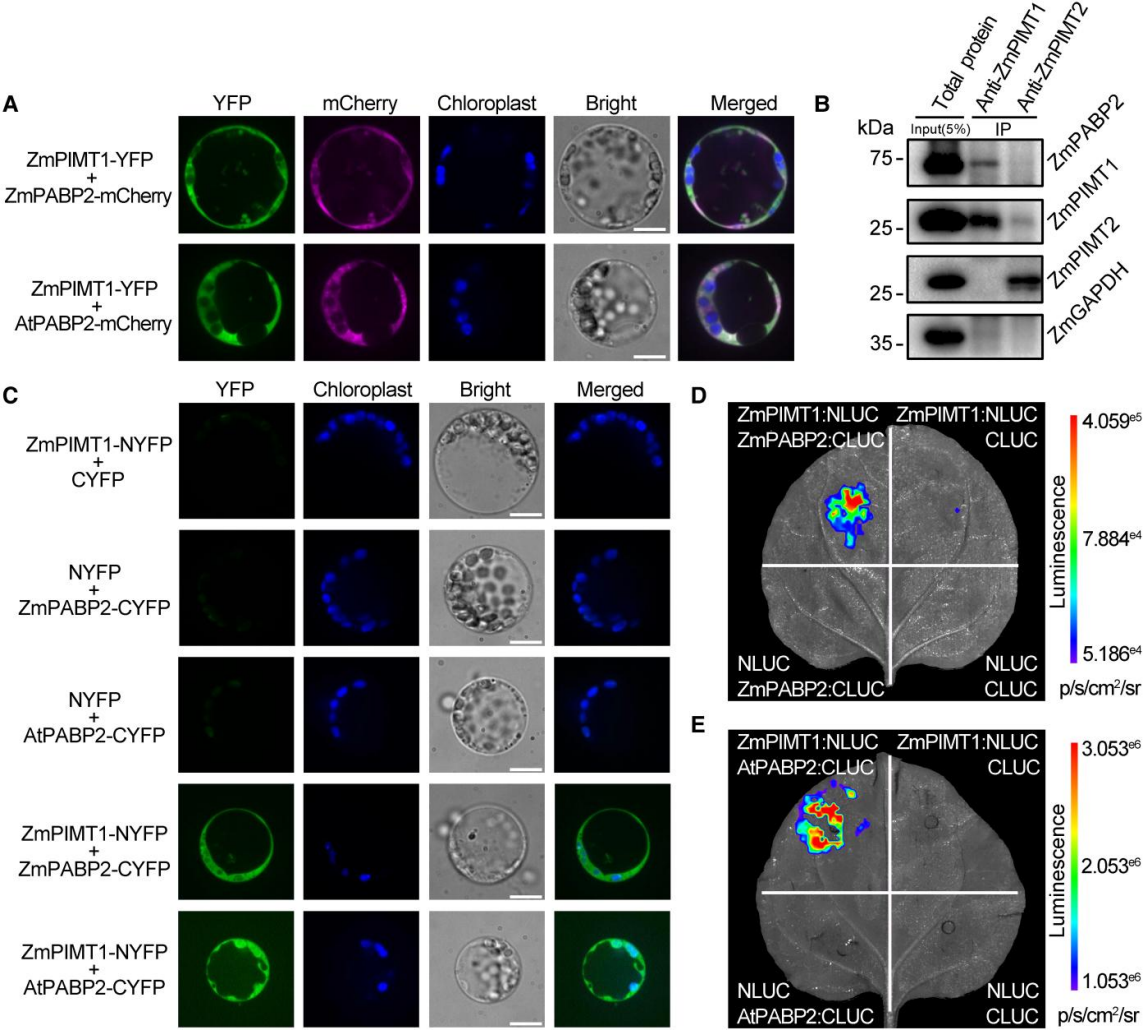
ZmPIMT1 interacts with PABP2. **A)** ZmPIMT1 and PABP2 are co-localized in the cytosol. ZmPIMT1 fused with YFP expression vector was co-transformed into maize protoplasts with ZmPABP2 fused with mCherry expression vector, or AtPABP2 fused with mCherry expression vector, respectively. The YFP fluorescence, mCherry fluorescence, and chloroplast autofluorescence signals were detected using confocal microscopy. A bright field image was taken and the white scale bar represents 10 *μ*m. The merged signal is the combination of all 3 fluorescent signals. Similar results were obtained in 3 independent experiments. **B)** Co-IP of ZmPABP2 by anti-ZmPIMT1 antibodies from 18 HAI-maize embryos. Protein extracts were incubated with anti-ZmPIMT1 or anti-ZmPIMT2 (as control) conjugated to protein A/G magnetic beads. Input (only 5% of that used for the experimental Co-IP) and immunoprecipitants (IP) were analyzed by immunoblotting using antibodies to ZmPABP2, ZmPIMT1, ZmPIMT2 or ZmGAPDH. **C)** The interaction between ZmPIMT1 and PABP2 was confirmed by BiFC. ZmPIMT1 was fused upstream to the N-terminal fragment of YFP (NYFP), and PABP2 was fused upstream to the C-terminal fragment of YFP (CYFP). The construct combinations were co-transformed into maize protoplasts. YFP fluorescence and chloroplast autofluorescence were detected using confocal microscopy, and a merged image was constructed from the 2 fluorescent channels. A bright-field image was obtained using differential interference contrast, with a white scale bar representing 10 *μ*m. Similar results were obtained in 3 independent experiments. **D)** Firefly luciferase complementation imaging (LCI) assay showing the interaction of ZmPIMT1 and ZmPABP2 in *N. benthamiana* leaves. ZmPIMT1:NLUC/ZmPABP2:CLUC, ZmPIMT1:NLUC/CLUC, NLUC/ZmPABP2:CLUC, and CLUC/NLUC were co-infiltrated into *N. benthamiana* leaves and examined after 48 h. Similar results were obtained in 3 independent experiments. Luciferase images were captured using a cooled charge-coupled device (CCD) imaging apparatus. **E)** Firefly LCI assay showing the interaction of ZmPIMT1 and AtPABP2 in a *N. benthamiana* leaf as described in **(D)** with the 1 difference being that the Arabidopsis PABP2 was used instead of the maize ortholog.

A total of 66 potential target proteins of ZmPIMT1 were identified in this exercise (Supplementary Table S2). Among the identified proteins, 4 *Zea mays* polyadenylate-binding proteins (ZmPABPs; Zm00001d005276, Zm00001d019824, Zm00001d025801, and Zm00001d003106) were identified (in bold in Supplementary Table S2). According to the expected protein size of ZmPABPs, the area where ZmPABPs were concentrated can be seen in stained SDS-PAGE gels, ZmPABP2 (Zm00001d005276) is the most frequently detected ZmPABP (Supplementary Table S2 and Supplementary Fig. S12). Phylogenetic analysis show that these 4 PABPs are all Class II PABPs, following the classification scheme described in Gallie and Liu (Gallie and Liu 2014) (Supplementary Fig. S13 and Supplementary File 1). AtPABP2 and AtPABP8 are also the Class II PABPs and are equally related to ZmPABP2 (Supplementary Fig. S13). The comparison of tissue expression profiling of ZmPABP2, AtPABP2 and AtPABP8 show that the expression of AtPABP2 is most similar to that of ZmPABP2 (Supplementary Fig. S14). Thus, we selected ZmPABP2 (Zm00001d005276) to prepare antibodies for further investigation. ZmPIMT1-YFP, ZmPABP2-mCherry, or AtPABP2-mCherry fusion protein expression vectors, were transformed into maize protoplasts to determine the subcellular localization of ZmPIMT1, ZmPABP2, and AtPABP2. The colocalized Yellow Fluorescent Protein (YFP) and mCherry fluorescence signals were predominantly observed in the cytosol (Fig. 5A).

To test whether ZmPIMT1 interacts with ZmPABP2 in vivo, proteins from 18 HAI maize embryos were immunoprecipitated with either anti-ZmPIMT1- or anti-ZmPIMT2 (control)-conjugated beads. After elution, the proteins were separated by 12% PAGE and transferred for immunoblot analysis using anti-ZmPIMT1, anti-ZmPIMT2, anti-ZmPABP2, and anti-ZmGAPDH antibodies. ZmPABP2 was precipitated by the anti-ZmPIMT1 antibody but not by the anti-ZmPIMT2 antibody, suggesting ZmPABP2 specifically interacts with ZmPIMT1 (Fig. 5B). Furthermore, BiFC assay was performed to confirm the interaction between ZmPIMT1 and PABP2 in maize protoplasts. The YFP signal was detected in protoplasts co-transformed with ZmPIMT1:NYFP and ZmPABP2:CYFP, or in protoplasts co-transformed with ZmPIMT1:NYFP and AtPABP2:CYFP, but not in protoplasts co-transformed with ZmPIMT1:NYFP and CYFP, neither in protoplasts co-transformed with NYFP and ZmPABP2:CYFP, or NYFP and AtPABP2:CYFP control constructs (Fig. 5C).

To provide additional confirmation of the in vivo interaction between ZmPIMT1 and ZmPABP2, a luciferase complementation imaging (LCI) assay was performed in *Nicotiana benthamiana* leaves. ZmPIMT1 was translationally fused to the N-terminus of LUCIFERASE (NLUC), and ZmPABP2 was translationally fused to the C-terminus of LUCIFERASE (CLUC) (Supplementary Fig. S15); vectors carrying these genes each driven by a strong plant promoter were co-infiltrated with each other and the presence of enzymatically-active luciferase assayed. Control infiltrations included co-infiltration of 1 or the other fusion construct with unmodified NLUC or CLUC in *N. benthamiana* leaves. Luminescence (and thus active luciferase) was readily detected when ZmPIMT1:NLUC was co-infiltrated with ZmPABP2:CLUC (Fig. 5D). In contrast, the co-infiltration of ZmPIMT1:NLUC with CLUC, or ZmPABP2:CLUC with NLUC, respectively, yielded no detectable luminescence (Fig. 5D). These results indicate that ZmPIMT1 interacts with ZmPABP2. In *N. benthamiana* leaves, ZmPIMT1:NLUC also interacted with the CLUC fusion of the *Arabidopsis* ortholog of ZmPABP2, the product of the At4g34110 gene (AtPABP2), as seen with the luminescence detected (Fig. 5E).

### Disruption of PABPs decreases seed vigor

**Figure 6.**
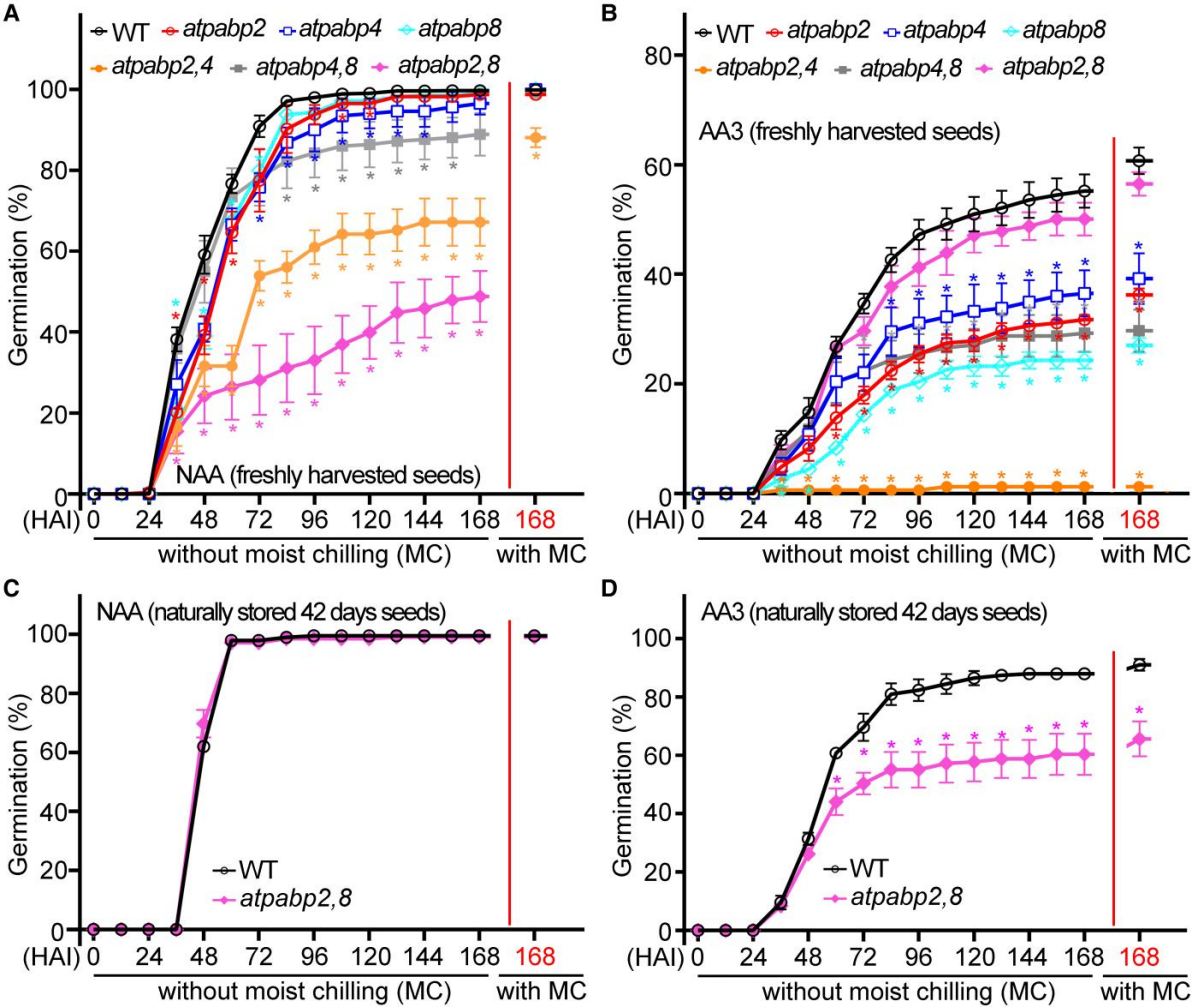
The seed vigor of *atpabp* single and double mutant seeds decreased after accelerated aging treatment. **A)** Seed germination percentages of WT, *atpabp* single and double mutants seeds that were freshly harvested without accelerated aging treatment (NAA). The completion of germination was calculated for every 12 h for 168 h and these numbers averaged and the standard error calculated and graphed. The values on the right side of the red line represent the germination percentages of the same seeds after 168 h, followed by an additional 3 days of moist chilling and a week of incubation at room temperature. A 2 tailed ANOVA (alpha = 0.05) was used to assess whether there were statistically significant differences among means and Dunnett's test was subsequently used with WT Col as the control and each of the *atpabp* single or double mutants compared to it. There were 3 replicates for each treatment and 80 seeds for each replicate. Those averages that differ significantly from the WT are denoted using a, asterisk the same color as the genotype symbol/line. **B)** A portion of the seeds from **(A)**. were treated over saturated KCl (75% RH) at 42 °C for 3 d (AA3) and then desiccated at room temperature for 24 h. The seed germination was then calculated as in (A). Note that the y-axis is scaled differently. **C)** Seed germination percentages of WT and *atpabp2,8* double mutants seeds that were dry after-ripened for 42 d at room temperature without accelerated aging treatment (NAA) or moist-chilling. The seed germination was then calculated as in **(A)**. **D)** A portion of the seeds from **(C)**. were treated over saturated KCl (75% RH) at 42 °C for 3 d (AA3) and then desiccated at room temperature for 24 h. The seed germination was then calculated as in (A).

To determine the role of PABP in regulating seed aging tolerance, the seed vigor of *atpabp2*, *atpabp4*, *atpabp8* single mutants, *atpabp2,4*, *atpabp4,8*, *atpabp2,8* double mutant seeds, and the WT (Col-0) control were compared. Without accelerated aging, freshly harvested seeds from WT completed germination to the same final percentage as seeds from all the single mutant lines while the double mutant lines shower considerably delayed germination than the WT (Fig. 6A). Moist chilling treatment of these seeds improved the final percentage germination 1 week later such that all but *atpabp2,4* seeds were statistically indistinguishable from WT (after the red line in Fig. 6A). These results demonstrate that 10 ± 5% (*atpabp4,8*), 20 ± 5% (*atpabp2,4*), and 50 ± 6% (*atpabp2,8*) of freshly harvested seeds were dormant. Following accelerated aging, all freshly harvested single and double mutant lines completed germination statistically significantly less than WT, except for the seeds from the *atpabp2,8* double mutant line which were statistically indistinguishable from WT (Fig. 6B). The seeds that had not completed germination following 7 d on germination media were moist chilled (3 d at 4 °C) to alleviate dormancy and then retested for the capacity to complete germination, but this improved final percentage germination 1 week later only marginally and not at all for *atpabp2,4* (after the red line in Fig. 6B). TTZ tests determined that the seeds failing to complete germination following accelerated aging, the initial germination trial, moist chilling for 3 d, and 1 week germination, were dead. Additionally, with or without accelerated aging, naturally aged (6 months dry storage at RT) *atpabp* single mutant seeds completed germination statistically significantly poorer than naturally aged WT seeds (Supplementary Fig. S16, A and B). Moist chilling treatment of these seeds did not improve the final percentage germination 1 week later (after the red line in Supplementary Fig. S16, A and B) and TTZ tests determined that most of the seeds failing to complete germination were dead.

Results from germination assays before and after moist chilling confirmed that the seeds from the double mutants, *atpabp4,8*, *atpabp2,4* and *atpabp2,8* exhibited an increase in seed dormancy relative to WT or single mutant seeds (Fig. 6A). The most dormant of these double mutants was *atpabp2,8*, on which we focused, alleviating dormancy of *atpapb2,8* and WT through dry after-ripening for 42 d and then retesting these genotypes for resilience to accelerated aging. After 42 d dry after-ripening, the completion of germination of WT and *atpapb2,8*, with or without moist chilling, were statistically indistinguishable (Fig. 6C; Supplementary Fig. S17). When these non-dormant seeds were subjected to accelerated aging for 3 d, the *atpabp2,8* seeds were statistically considerably less resilient than WT (Fig. 6D). When these seeds were then subjected to moist chilling for 3 d before a second week of germination trials, there was no substantial improvement in final germination percentage of either seedlot (after the red line of Fig. 6D). TTZ tests ascertained that those seeds failing to complete germination were dead.

### ZmPIMT1 stabilizes PABP2 RNA binding capacity

The interaction between ZmPIMT1 and 2 PABP2 orthologs promotes the hypothesis that ZmPIMT1 may reverse aging-associated damage of PABP2. To test this, the effects of ZmPIMT1 on the activities of PABP2 were studied. Initial attempts to conduct these experiments using ZmPABP2 were unsuccessful as bacterial cells expressing this protein ceased growth, thereby abrogating the purification of this protein from over-expressing cells. Since ZmPIMT1 also interacts with the *Arabidopsis* PABP2 ortholog (Fig. 5, A and C, and E), we expressed and purified AtPABP2 (Supplementary Fig. S18).

Methylation assays using radioactive S-adenosylmethionine showed that recombinant ZmPIMT1 can identify and methylate isoAsp residues in the isoAsp-containing peptide VYP-(isoD)-HA (VisoD), signifying that it is active as a PIMT enzyme (Supplementary Fig. S19). ZmPIMT1 was able to methylate recombinant AtPABP2 that had been subjected to an aging treatment, as well as the unaged AtPABP2 (Supplementary Fig. S19).

To explore the effects of ZmPIMT1 repair on the RNA binding activity of AtPABP2, we first determined whether ZmPIMT1 could bind poly(A) mRNA (negative control). For the RNA binding determinations, we used Temperature Related Intensity Change (TRIC) assays with a Dianthus Pico instrument; in these assays, a mimic of a natural mRNA with a 3' 40 nt poly(A) tracts was synthesized and labeled with cy5. In the presence of either AdoMet or AdoHcy, ZmPIMT1 did not bind poly(A) mRNA (periodically checked over a 38 h time course; Supplementary Fig. S20). Unaged AtPABP2 could bind poly(A) mRNA after 1 or 4 h assay regardless of ZmPIMT1 presence with AdoMet or AdoHyc (Table 1). Because aging through incubation at 37 °C for 3 h (used to expedite isoAsp formation) precipitated some of the thermo-fragile AtPABP2 but did not enhance isoAsp production over that of unaged AtPABP2 (Supplementary Fig. S19), aliquots of AtPABP2 were aged at 25 °C for 3 h (to avoid protein precipitation), before commencing the RNA binding assay, these assays were extended to 16 or 22 h using unaged or 25 °C-aged AtPABP2. Nonetheless, no legitimate *K_D_*s were acquired for AtPABP2 aged for 3 h at 25 °C, whether in the presence of ZmPIMT1 or not, with AdoMet or AdoHyc, for either 1 or 4 h incubation (Table 1).

**Table 1.** Time course of K_D_s (± S.E. µM; n = 3) of unaged or aged (3 h at 25 °C) AtPABP2 for poly(A) mRNA without or with ZmPIMT1 in the presence of AdoMet or AdoHcy

Treatment	Incubation duration (h)	AtPABP2 alone	AtPABP2 with ZmPIMT1 and AdoMet	AtPABP2 with ZmPIMT1 and AdoHcy
Unaged AtPABP2	1	2.853 ± 0.458 A	2.279 ± 0.349 A	2.763 ± 0.393 A
	4	1.407 ± 0.584 AB	1.680 ± 0.412 A	1.070 ± 0.103 A
	16	0.385 ± 0.054 Ba	0.198 ± 0.062 Ba	0.584 ± 0.393 Aa
	22	0.369 ± 0.137 Ba	0.166 ± 0.075 Ba	1.095 ± 0.987 Aa
Aged AtPABP2	1	–	–	–
	4	–	–	–
	16	0.814 ± 0.439 a	0.926 ± 0.070 b	3.300 ± 1.786 a
	22	3.037 ± 2.076 a	0.975 ± 0.211 b	6.800 ± 4.806 a

The “–” indicates that no legitimate *K_D_* was obtained for these measurements (Supplementary Fig. S21). Different upper-case letters following the *K_D_* estimates represents statistically significantly different *K_D_*s among incubation durations within a column (Unaged AtPABP2 only) based on Scheffe's multiple pairwise comparisons (alpha = 0.05). Different lower-case letters following the *K_D_* estimate for 16 and 22 h incubation only, denotes statistically significant differences in *K_D_*s between unaged and aged samples for a specific incubation period (16 or 22 h) within a column (i.e. an AtPABP2 treatment: alone, or ZmPIMT1 with AdoMet or ZmPIMT1 with AdoHcy).

**Figure 7.**
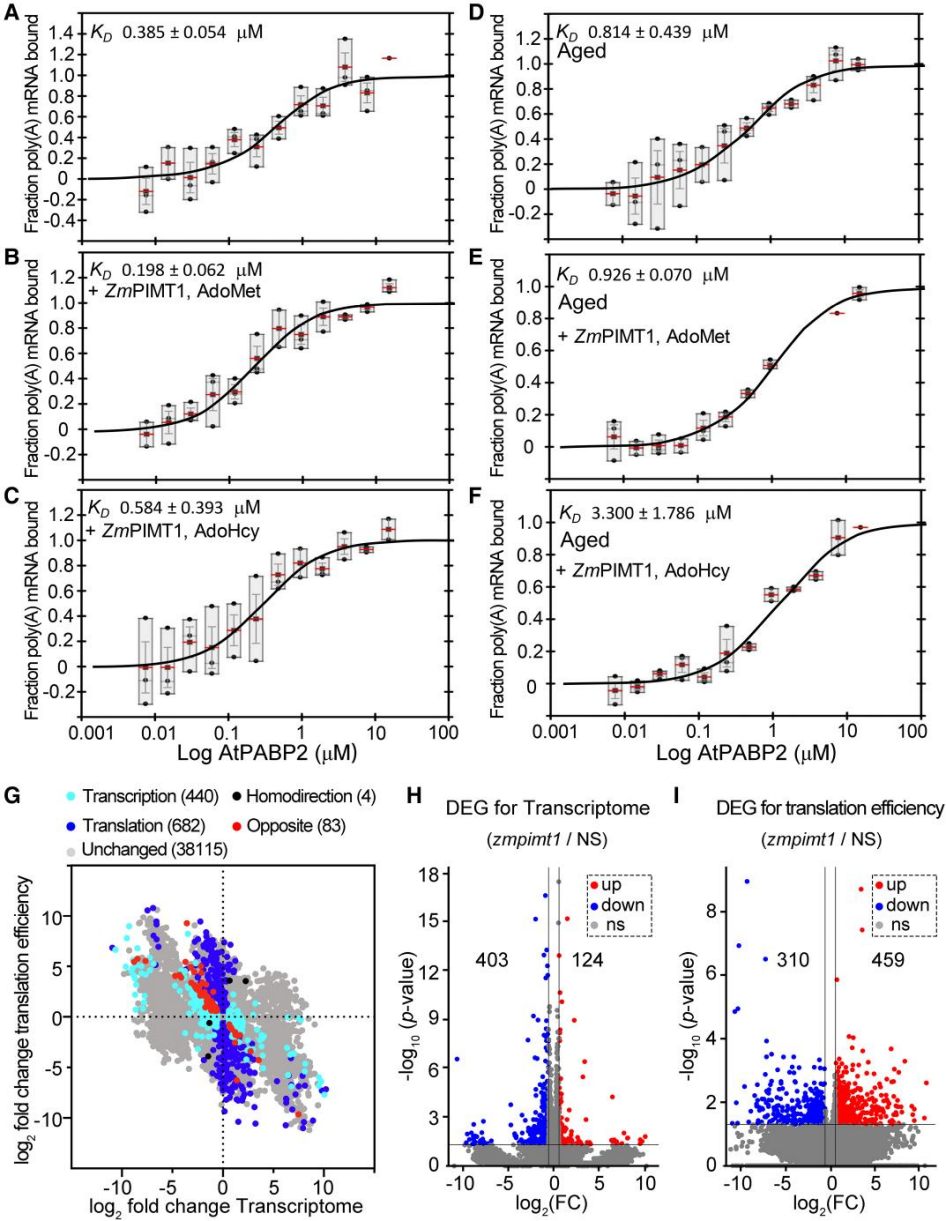
ZmPIMT1 stabilizes AtPABP2 poly(A) mRNA binding capacity. **A-C)** The quantification of poly (A) mRNA bound by unaged AtPABP2 when the mRNA is incubated with only unaged AtPABP2 **(A)** or with constant maintenance by ZmPIMT1 in the presence of AdoMet **(B)** or with constant maintenance by ZmPIMT1 in the presence of AdoHcy **(C)**. In the box plot, the box's lower and upper edges represent the minimum and maximum values of the dataset, respectively, with an internal red line marking the mean. The whiskers extending from the mean represent the stand error bars (n = 3). **D-F)** The quantification of poly (A) mRNA bound by aged AtPABP2 when the mRNA is incubated with only aged AtPABP2 **(D)** or with constant maintenance by ZmPIMT1 in the presence of AdoMet **(E)** or with constant maintenance by ZmPIMT1 in the presence of AdoHcy **(F)**. The estimate is the average *K_D_* from the 3 replications and the standard error of the mean. The *K_D_* of unaged AtPABP2 for poly(A) mRNA, in the presence of ZmPIMT1 and AdoMet, is statistically significantly less (ANOVA, α = 0.05) than the *K_D_* of aged AtPABP2 also in the presence of ZmPIMT1 and AdoMet (Table 1). In the box plot, the box's lower and upper edges represent the minimum and maximum values of the dataset, respectively, with an internal red line marking the mean. The whiskers extending from the mean represent the stand error bars (n = 3). **G)** The differences in the transcriptome and translation efficiency of the aged seed embryo between the *zmpimt1* mutant and its null segregant. Five separate categories were designated: (1) Transcription for those genes with significant changes only in the transcriptome; (2) Translation for those genes with significant changes only in translation efficiency; (3) Homodirection for those genes with significant differences in both omics analyses, and for which both omics either decreased or increased; (4) Opposite for those genes with significant differences in the 2 omics analyses, and for which when 1 omic analysis showed an increase, the other decreases or vice versa; and (5) Unchanged for those genes with no significant difference in either of the omics. **H)** Volcano plot of differentially expressed genes (transcriptome) in aged embryos between *zmpimt1* and its null segregant (NS) control lines. Each point represents 1 gene. The grey points portray genes not differentially expressed between *zmpimt1* and its null segregant (NS) control lines. The blue points stand for downregulated genes. The red points denote upregulated genes. **I)** Volcano plot of differentially translation efficiency genes in aged embryos between *zmpimt1* and its null segregant (NS) control lines. Each spot represents 1 gene. The black points are genes not differentially expressed between *zmpimt1* and its null segregant (NS) control lines. The blue points are downregulated genes. The red points are upregulated genes.

For either unaged or aged AtPABP2, within an incubation duration (rows in Table 1), no statistically significant differences were observed for *K_D_*s due to treatment (alone or with ZmPIMT1 with AdoMet or with AdoHcy; Table 1; Fig. 7, A to C). Although initial *K_D_*s (1 or 4 h incubation) of aged AtPABP2 for poly(A) mRNA were not generated by TRIC assay, after an additional 16 h incubation at 25 °C a *K_D_* was detectable which was neither mitigated nor worsened by incubation with ZmPIMT1 with AdoMet or AdoHcy (Fig. 7, D to F). Examining the raw well scans and TRIC traces, at 1 and 4 h incubation, aged AtPABP2 at higher concentrations generated anomalous XYZ-scans that were excluded from the TRIC assay (Supplementary Fig. S21). These anomalies dissipated over prolonged incubation even without ZmPIMT1 and AdoMet; although of the 3, treatment with ZmPIMT1 and AdoMet generated the most homogenous XYZ-scans, fewest unusable data points, and most robust *K_D_* estimates (least standard error; Table 1; Supplementary Table S3 (16 h analyzed)) following prolonged incubation (Supplementary Fig. S22 (22 h depicted); Table 1). At 22 h incubation, the *K_D_* of unaged AtPABP2 for poly(A) mRNA remained similar to that after 16 h incubation (Table 1). This was also the case for AtPABP2 *K_D_*s for poly(A) mRNA in the presence of ZmPIMT1 and AdoMet or AdoHyc although the latter was quite variable (Table 1; Supplementary Fig. S22 A-C). If AtPABP2 had first been aged prior to incubation, its *K_D_* for poly(A) mRNA, either alone or in the presence of ZmPIMT1 and AdoHyc, was statistically similar after 22 h, relative to 16 h, of incubation at 25 °C but with a tendency to become more variable (Table 1; Supplementary Fig. S22). In the presence of ZmPIMT1 and AdoMet, the *K_D_* was also uninfluenced relative to its value for this treatment after 16 h incubation (Table 1; Supplementary Fig. S22 D-F). Only in the presence of ZmPIMT1 and AdoMet did unaged AtPABP2 have a statistically significantly lower *K_D_* for poly(A) mRNA than aged AtPABP2 at both 16 and 22 h (Table 1; Fig. 7 and Supplementary Fig. S22, compare B with E). Additionally, the *K_D_* of unaged AtPABP2 for poly(A) mRNA significantly decreased during incubation without ZmPIMT1 as well as when chaperoned by ZmPIMT1 in the presence of AdoMet (Table 1). This was not the case when ZmPIMT1 activity was poisoned with AdoHcy (Table 1). These were the only statistically significantly different *K_D_*s registered among treatment comparisons.

The interaction between ZmPIMT1 and ZmPABP2 (as well as AtPABP2) raises the possibility that ZmPIMT1 may function, through ZmPABP2, in regulating RNA abundance (stability), mRNA translation, or both. To explore this, both RNA-seq and Ribosome Nascent-chain Complex sequencing (RNC-seq) were performed using embryos that had been subjected to 6 d of aging. The rationale for this study is the expectation that, should ZmPIMT1 be important for ZmPABP2 function, then there should be less active (more damaged) ZmPABP2 in aged embryos in the *zmpimt1* mutant than in the NS control. Since plant PABP2 s function in mRNA stability and translation, lower ZmPABP2 activity might elicit altered mRNA accumulation or translation. The expression or translation efficiency of 1,209 genes changed in *zmpimt1* compared with the NS control while 38,115 genes remained unchanged (Fig. 7G, Supplementary Table S4). The mRNA levels of 527 genes were changed, and 403 genes had down-regulated expression in *zmpimt1* relative to the NS (Fig. 7H). The translation efficiency of 769 genes were changed, of which 310 transcripts had decreased translation in *zmpimt1* relative to the NS (Fig. 7I). Gene Ontology (GO) analysis of the transcriptome shown that down regulated genes were primarily associated with secondary active transmembrane transporter activity (GO: 0015291), plasma membrane (GO: 0005886), and proton transmembrane transport (GO: 1902600) (Supplementary Fig. S23A). GO analysis of translation efficiency showed that down regulated genes were primarily associated with oxidoreductase activity (GO: 0016491) and post-embryonic plant organ development (GO: 0090696) (Supplementary Fig. S23B).

### *ZmPIMT1* has not undergone strong selection during maize domestication

**Figure 8.**
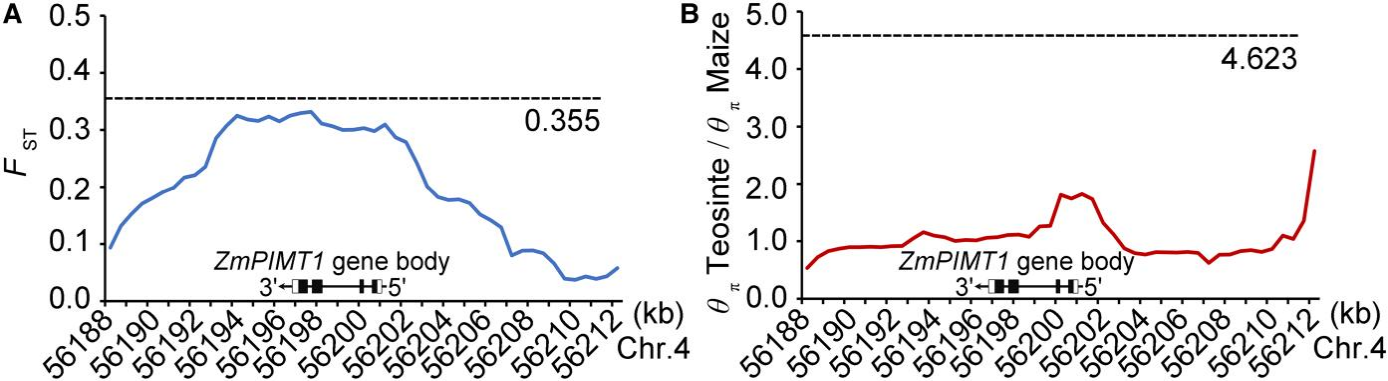
Characterization of the *F*_ST_ (Fixation index) and DNA polymorphism rates between teosinte and maize populations. **A)** Characterization of the *F*_ST_ between teosinte and maize cultivar entries across the *ZmPIMT1* locus (bottom). *F*_ST_ for evolution was calculated using a 3000-bp sliding window with a 100-bp step. The structure of the *ZmPIMT1* (Zm00001d049966) gene is illustrated at the bottom. The dotted line represents the significant threshold estimated by the permutation test of evolution. **B)** Characterization of the nucleotide diversity (*θ_π_*) ratio between teosinte and maize cultivar entries across the *ZmPIMT1* locus (bottom). The nucleotide diversity ratio was calculated using a 3000-bp sliding window with a 100-bp step. The structure of the *ZmPIMT1* (Zm00001d049966) gene is illustrated at the bottom. The dotted line represents the significant threshold (at *P* < 0.05) estimated by the maize neutral domestication bottleneck model.

To determine whether the *ZmPIMT1* gene has undergone selection during maize domestication, we analyzed nucleotide diversity around the *ZmPIMT1* locus using the third-generation *Zea mays* haplotype map (HapMap 3), which contains genome sequences of 18 teosintes and 1,034 maize lines (Bukowski et al. 2018). First, we used *F*-statistics (Fixation index; *F*_ST_) to assess the genetic differentiation of the *ZmPIMT1* gene. The max *F*_ST_ value among the *ZmPIMT1* gene body is 0.332, which was smaller than the background value of 0.355 for the whole genome (Fig. 8A). Furthermore, a sliding-window analysis revealed elevated DNA polymorphism rates between teosinte and maize lines over the *ZmPIMT1* gene region and 10.0 kb flanking region (*θ*π teosinte/*θ*π maize = 1.824; smaller than the 4.623 which is the background value of the whole genome). These results suggest that *ZmPIMT1* was left largely unselected during maize domestication (Fig. 8B).

**Figure 9.**
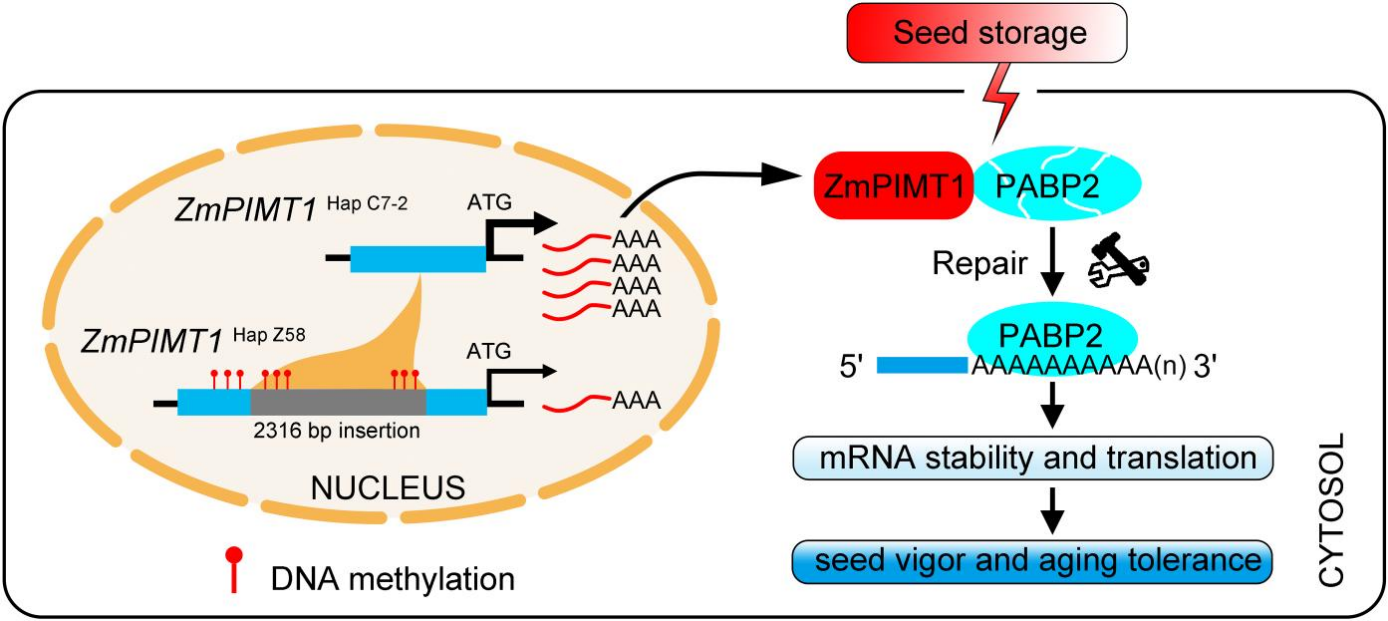
A proposed working model of how natural variation in the *ZmPIMT1* promoter enhances seed aging tolerance by regulating PABP2 repair in maize. The promoter of *ZmPIMT1*  ^Hap C7–2^, which is without the 2316 bp inserted fragment, increases *ZmPIMT1* expression by avoiding DNA methylation of the promoter, enhancing ZmPIMT1 repair of damaged PABP2, which, in turn, stabilizes mRNA, facilitates translation, and positively regulates seed aging tolerance.

Collectively, our results demonstrate that natural variation in the *ZmPIMT1* promoter influences seed aging tolerance by modulating ZmPIMT1 expression. ZmPIMT1, in turn, stabilizes PABP2 activity, ensuring efficient mRNA translation during germination (Fig. 9). These findings provide a molecular framework for improving seed longevity in maize and other crops, beyond PIMT as was already outlined (Wu et al. 2017).

## Discussion

### Why has the seed vigor trait not undergone selection during maize domestication?

In stark contrast to its ancestor, teosinte, modern maize has been shaped through domestication and selection for key crop traits, such as kernel size, row number, lack of seed dormancy, and flowering time, culminating in the world's most productive crop (López et al. 2011; Hufford et al. 2012; Huang et al. 2018; Wang et al. 2019). Seed vigor, a complex trait encompassing aging tolerance, seed dormancy, viability, rapid germination, and seedling establishment, is a less conspicuous, yet pivotal, trait (Wu et al. 2017) in the early domestication and improvement of maize as a source of calories.

Traditional corn production ensured sufficient seedlings through multi-seed sowing and thinning. This may be one of the reasons why seed vigor may not have been the focus of targeted selection during domestication. This, in turn, may explain the results shown in Fig. 8, indicating that the *ZmPIMT1* promoter has not been subject to selection, even though variation exists and is highly related to its expression and seed vigor in maize populations (Figs. 1 and 2). Other vigor-associated loci have been reported to be under selection. For example, the AP2 transcription factor ZmRAP2.7, which regulates seed vigor, has undergone selection during improvement (Guo et al. 2024). However, in addition to regulating seed vitality, *ZmRAP2.7* regulates flowering time and brace root development. Indeed, *ZmRap2.7* selection is much more associated with flowering time (Guo et al. 2024). These findings shed light on the implications for maize domestication and improvement, providing valuable insights for future research and breeding strategies.

In modern maize production, the single seed sowing method amplifies the need for high seed vigor. High-vigor seeds ensure uniform germination synchronizing germination completion and seedling emergence. It enhances robust early growth, resilience to stress, and, most importantly, it maximizes yield potential. *ZmPIMT1*  ^Hap C7–2^ is a molecular marker that will be useful for cultivating germplasm resources with high seed vigor in maize that could enhance the adoption of single-seed sowing.

### The function of ZmPIMTs in the regulation of maize seed vigor

In prokaryotes and animals, PIMTs are mainly encoded by single genes, while angiosperms possess 2 isoforms (*PIMT1* and *PIMT2*) (O'Connor 2006); these 2 *PIMT* isoforms are thought to be the result of a duplication event from an ancestral *PIMT* gene (Xu et al. 2004). PIMT2 possesses a unique N-terminal extension compared with PIMT1 in plants (Supplementary Fig. S11A) that serves as a mitochondrial targeting sequence that facilitates the transport of ZmPIMT2 into mitochondria (Zhang et al. 2023).

The highest activity of PIMT among maize tissues was found in the mature, desiccated seed, closely followed by 24 HAI seeds (Thapar et al. 2001). While this does not correlate well with the ZmPIMT1 protein abundance (Supplementary Fig. S6), the activity from the paralogous PIMT (ZmPIMT2) may be the reason for the discrepancy; the expression of *ZmPIMT2* remained at a higher level at 12 or 24 HAI, predominantly expressed in maize embryos (Zhang et al. 2023). Both ZmPIMT1 and ZmPIMT2 can regulate seed viability; however, the regulatory mechanisms are likely different. ZmPIMT1, located in the cytoplasm (Supplementary Fig. S24), interacts with ZmPABP2 and stabilizes PABP2 poly(A) RNA binding capacity—imparting greater seed vigor (Fig. 5). ZmPIMT2 binds 3-METHYLCROTONYL COA CARBOXYLASEα (ZmMCCα) in mitochondria, repairs isoAsp damage, and positively affects maize seed vigor (Zhang et al. 2023).

Seed germination requires protein synthesis and energy. During the early stages of seed imbibition, protein synthesis relies on translation from stored mRNAs in seeds (Bai et al. 2020). Besides ZmPABPs, several other proteins which regulate mRNA stability or translation were identified to interact with ZmPIMT1 (Supplementary Table S2), including DEAD-BOX ATP-DEPENDENT RNA HELICASE 13 (Zm00001d003031), TRANSLATIONALLY-CONTROLLED TUMOR PROTEINS (Zm00001d014957 and Zm00001d014954), EUKARYOTIC TRANSLATION INITIATION FACTOR 5A (Zm00001d006760), and 5 ELONGATION FACTORS (Zm00001d036959, Zm00001d016358, Zm00001d009480, Zm00001d022134, Zm00001d022513). Besides protein synthesis, seed germination requires energy from stored carbohydrates. Several related proteins involved in starch and sucrose metabolism were also determined to interact with ZmPIMT1 (Supplementary Table S2), including GRANULE-BOUND STARCH SYNTHASE 1 (Zm00001d045462, *WAXY1*), SUCROSE SYNTHASE (Zm00001d045042) and GLUCOSE-1-PHOSPHATE ADENYLYLTRANSFERASE (Zm00001d044129). These results suggest that ZmPIMT1 protects the mRNA-associated proteins and key enzymes associated with carbohydrate metabolism, which may contribute to positively regulating seed vigor.

While our study primarily focuses on the critical role of the ZmPIMT1–PABP2 interaction in maintaining protein integrity during seed aging, we fully acknowledge that seed vigor is a complex, polygenic trait governed by a network of proteins and pathways. The ZmPIMT1-PABP2 interaction serves as a mechanistic cornerstone in regulating seed vigor through translational maintenance. It operates within a broader, systems-level network (the natural protection and repair mechanism) involving protein repair (PIMTs, MSRs, chaperones) (Hazra et al. 2022), metabolic protection (RFOs, antioxidants, LEAs) (Sattler et al. 2004; Li et al. 2017), all subject to regulatory control (ABA signaling, epigenetics). Our study provides 1 paradigm-ZmPIMT1-as a key player in this network. Future efforts could aim at integrating these pathways through multi-omics and gene-editing strategies to develop maize varieties with superior seed performance under climate stress.

### PABPs mediate seed longevity and dormancy dynamics

Seeds in which any one of the class II PABPs were compromised due to mutation did not withstand natural- or accelerated aging as well as WT (Fig. 6A). Seeds of the non-dormant double mutants were also more susceptible to controlled-deterioration than WT (Fig. 6C, D). The fact that non-dormant single and double mutant lines all withstood accelerated aging less well than WT emphasizes how important the interaction of these class II PABPs with mRNA is for longevity. While the PABPs are also involved in mRNA decay (Gallie 2017), they are particularly important for mRNA translation (Belostotsky 2003) which is, itself, required for the completion of germination (Rajjou et al. 2004). This is an example of Job's rule (Dirk and Downie 2018), underscoring the importance of the seed natural protection and repair mechanism (the current example emphasizing repair) that safeguards the proteins comprising the translational apparatus, foundational to normal seed longevity.

Without accelerated aging, freshly harvested *atpapb4,8*, *atpabp2,4* and *atpabp2,8* double mutant seeds completed germination to a lesser degree than all other lines (Fig. 6A). Seeds from these genotypes subsequently responded to moist chilling (comparing 168 h before to after the red line in Fig. 6A), emphasizing a role for these type II PABPs in dormancy alleviation, their involvement dampening seed dormancy establishment late during development, or both. This relationship to seed dormancy was only unveiled when 2 type II PAPBs were simultaneously dysfunctional as there was no indication that any of the single mutants experienced enhanced seed dormancy. Thus, there is no apparent continuum of dormancy increase with any single mutant (*atpabp2*, or *4*, or *8*) leading to the dormancy exhibited by the double mutants. There also appears to be a hierarchy of penetrance where dormancy is invoked least in *atpabp4,8*, followed by *atpabp2,4*, and most severely in *atpabp2,8*. Despite the enhanced seed dormancy brought about by down-regulation of any 2 of the type II PABPs, these mutations did not interfere with the capacity of dormancy to be overcome through moist chilling (Fig. 6A; Supplementary Fig. S17) or, for *atpapb 2,8*, dry after-ripening (Fig. 6C). Our contention that the PABPs are involved in mitigating seed dormancy is justified given that freshly harvested seeds, failing to complete germination, did not do so due to permanent dysfunction brought about by the mutations but rather responded normally to 2 different stimuli known to alleviate seed dormancy in this species.

Following 3 day-accelerated aging, freshly harvested *atpabp2,8* seeds completed germination as well as without the stress and similar to WT (Fig. 6B). Such was not the case for freshly harvested *atpabp2,4* seeds which were devastated by accelerated aging while *atpabp4,8* seeds were intermediate between those of the other double mutant lines (Fig. 6B). Dormant seeds withstand abiotic stress to a greater degree than non-dormant seeds, an attribute correlated with persistence in soil seed banks of many, but not all, species (Gioria et al. 2020). Abiotic stress, including accelerated aging, is able to alleviate dormancy in many species (Blanche et al. 1990; Leinonen 1998). We suggest that the dormant *atpabp2,8* seeds withstood accelerated aging and, concomitantly, lost dormancy, during the 3 d stress, completing germination thereafter to the same extent as the non-dormant WT seeds. Indeed, upon dry after-ripening, relieving *atpabp2,8* dormancy, these seeds were more susceptible to accelerated aging than WT (Fig. 6D). The dormancy displayed by the *atpabp2,4* seeds (168 h before and after the red line Fig. 6A) was insufficient to protect them from accelerated aging (Fig. 6B). The reason for this discrepancy between dormant seeds of the 2 double mutant lines is unclear.

While our study demonstrates that ZmPIMT1 stabilizes ZmPABP2 activity to enhance seed vigor, a critical limitation is the lack of a maize *pabp2* mutant to directly validate this mechanism in maize. To circumvent this, we employed *Arabidopsis* atpabp mutants, which revealed that disruption of PABP2 orthologs (AtPABP2/4/8) reduces seed longevity and stress resilience (Fig. 6). Although PABP2 is conserved across plants, maize and *Arabidopsis* may differ in functional and spatial redundancy among PABP family members. Functional compensation by other PABPs in *Arabidopsis* (e.g. AtPABP4/8) might mask stronger phenotypes expected in maize. In addition, *Arabidopsis* seeds lack the dead starchy endosperm of maize, this would be 1 compartment in maize seeds where dysfunction of either ZmPIMT1 or ZmPABPs would pose less of a problem for seed longevity. Despite these limitations, the consistency of our findings-where both *ZmPIMT1* knockdown (Fig. 4) and AtPABP2 disruption (Fig. 6) reduce seed vigor-supports a conserved role for PABP2 in seed aging tolerance. Future studies could prioritize generating maize *pabp2* mutant (e.g. via CRISPR-Cas9) to confirm this mechanism in a crop-specific context.

### Implications of the association of ZmPIMT1 with ZmPABP2

PABPs are highly conserved in eukaryotes but appear absent from prokaryotes (Mangus et al. 2003). In plants, PABPs can be classified into 3 groups (Gallie and Liu 2014); these are typified by the *Arabidopsis PAB6* and *PAB7* genes (Class III), *PAB1*, *PAB3*, and *PAB5* genes (Class I), and *PAB2*, *PAB4*, and *PAB8* genes (Class II) (Gallie and Liu 2014; Supplementary Fig. S13). The 4 ZmPABPs which co-purified with ZmPIMT1 (Supplementary Table S2) are all Class II PABPs (Supplementary Fig. S13). Class II PABP genes are expressed throughout the plant (Supplementary Fig. S14) and have partially redundant functions (Gallie 2017). For example, these proteins function in mRNA translation via specific interactions with subunits of the initiation complex (Gallie 2014; Gallie 2018). They also play roles in plant virus life cycles by promoting specialized modes of translation initiation (Khan and Goss 2019; Khan et al. 2024).

PABPs bind to the poly(A) tail and regulate mRNA translation and stability. The RNA-seq and RNC-seq results showed that the mRNA stability and mRNA translation efficiency were reduced in the *zmpimt1* mutant (Fig. 7, G to I). During early seed imbibition, sugar metabolism, and mitochondrial energy metabolism are required to meet high cellular energy demands. Among the potential target genes, we found that the mRNA stability of *ALPHA-GALACTOSIDASE 2* (Zm00001d032605) and *SUCCINYL-COA LIGASE ADP-FORMING SUBUNIT BETA* (Zm00001d037006) was reduced (Supplementary Table S4). The mRNA translation efficiency of *BETA-AMYLASE* (Zm00001d019756) and *MITOCHONDRIAL SUCCINATE-SEMIALDEHYDE DEHYDROGENASE* (Zm00001d015406) was downregulated (Supplementary Table S4). These results suggest 1 means by which PABP2 regulates seed vigor is through regulating the mRNA stability and translation efficiency of genes encoding proteins responsible for energy metabolism.

Imbibing maize seeds experience a dramatic global upregulation of protein synthesis during the first 24 h of germination (Jimenez-Lopez et al. 2011). The interaction of ZmPIMT1 and Class II PABPs raises the possibility that ZmPIMT1 contributes to seed vigor in part by preserving and promoting the capabilities of maize PABPs to function in protein synthesis, thus allowing for efficient translation of stored mRNAs early in the germination process. PIMT1 can repair heat-treated *Arabidopsis* RNA helicase, PRH75 (Nayak et al. 2013) in a manner similar to the repair of ZmPABP2 by ZmPIMT1 shown here. We have detected at least 1 acetylation site, 3 carbamidomethylation sites, 21 Asn deamidation sites, and 10 oxidation modification sites extant in ZmPABP2 during seed storage (Supplementary Fig. S25). A comparison of the relative isoAsp abundance showed that ZmPABP2 was more susceptible to isoAsp formation in the *zmpimt1* mutant line than its null segregant (NS) control line (Supplementary Fig. S26). Interestingly, 5 of the 21 Asn deamidation sites lie within the third and fourth RNA recognition motifs (RRMs) of the protein (Supplementary Fig. S27). In animal PABPs, the first 2 RRM domains are responsible for poly(A)-specificity while the 3rd and 4th RRMs contribute to overall RNA binding activity. The effects of aging on RNA binding by AtPABP2 may reflect altered activities of these 2 domains, such that poly(A)-specific binding is retained, albeit with reduced affinity owing to the alterations of RRMs 3 and 4 (Supplementary Fig. S27). In any case, our results provide support for a role for ZmPIMT1 in restoring or maintaining active PABP throughout seed maturation and early germination.

## Materials and methods

### Plant material and growth conditions

Maize (*Zea mays* L.) *zmpimt1* mutant seeds (mu1028445) in W22 background were obtained from the Maize Genetics Cooperation Stock Center (https://maizegdb.org/data_center/stock) and were maintained in Yangling (Shaanxi Province), China. The homozygous *zmpimt1* mutant and its counterpart NS controls were obtained by self-pollination of a heterozygous mutant. The characterization of the *zmpimt1* mutants was performed by PCR using primers F1, R1, and Mu-TIR-specific primer TIR6 (Fig. 4 and Supplementary Table S1) (Settles et al. 2007).

The maize inbred line population, which was representative of the whole association-mapping panel composed of 368 diverse maize inbred lines (Fu et al. 2013), was planted in a field in Yangling (Shaanxi Province). Zhengdan 958 hybrid was obtained by crossing Zheng58 as the maternal parent and Chang7-2 as the male parent. Zhengdan 958 hybrids were self-crossed to obtain F2 generation isolated population, the isolated population was identified by using primers F4 and R4 (Supplementary Table S1) to obtain *ZmPIMT1*  ^Hap Z58^ and *ZmPIMT1*  ^Hap C7–2^ lines, respectively. The identified lines were continuously self-cross-bred to the F4 generation before testing seed vigor.

The mutant *Arabidopsis* lines were either the kind gift of Prof. Albrecht von Arnim, Biochemistry and Cellular and Molecular Biology, University of Tennessee, Knoxville (naturally aged WT and single mutant seeds; *atpabp2*, *atpabp4*, *atpabp8*) or purchased from the *Arabidopsis* Biological Resource Center (CS8174: *atpabp2* (At4G34110; SALK_026293); CS8175: *atpabp4* (At2G23350; SALK_113383); CS8176: *atpabp8* (At1G49760; SALK_022160); CS8177: *atpabp2,4*; CS8178: *atpabp4,8*; and CS8179: *atpabp2,8* derived from crosses of the above mentioned single mutants). The purchased single mutants are the same accessions as those gifted by Prof von Arnim. The exonic positions of the interrupting T-DNA were published previously (Dufresne et al. 2008). Purchased seeds were sown in pots and plants grown in a growth room to maturity under constant illumination (photosynthetic photon flux density 92.3 *µ*M•m^-2^•sec^−1^) at the same time, in the same container. Seeds were harvested on the same day and kept at 22 °C in paper envelopes. Germination assays, before and after accelerated aging, were conducted 1 week following harvest (freshly harvested seeds) and, for WT and *papb2,8* double mutant seeds, redone 42 d following harvest (dry after-ripened seeds).

*Nicotiana benthamiana* plants were grown under 16 h/8 h of light/dark at 22 °C as described by Su *et al*. (Su et al. 2021).

### RNA extraction and real-time RT-PCR

Total RNA from 0.05 g of maize embryos was extracted following a published protocol (Li et al. 2017). DNase-treated, total RNA (2 *μ*g) was used to synthesize cDNA using the Transcriptor First Strand cDNA Synthesis kit (Roche, Switzerland). The synthesized cDNA (20 *μ*L) was diluted into 0.6 mL for subsequent use in real-time RT-PCR. The real-time RT-PCR analysis of the *ZmPIMT1* and *2* transcript was performed using the primer pair, RT-*ZmPIMT1* or *2*-F and RT-*ZmPIMT1* or *2*-R. The expression of both *ZmPIMT1* and *2* was normalized to the mRNA amounts of *ZmGAPDH* in maize (using primers, RT-*ZmGAPDH*-F and RT-*ZmGAPDH*-R; for all 3 primer sets, Supplementary Table S1). Data analysis followed a published protocol (Guo et al. 2021).

### Vector construction

Please refer to the supporting information (Supplementary method S1).

### ZmPIMT1 and ZmPABP2 antibody preparation, protein extraction, and western blot

Please refer to the supporting information (Supplementary method S2).

### ZmPIMT1 expression genome-wide association (eGWAS) mapping and SNP annotation

A total of 2,965,911 SNPs (MAF ≥ 0.05; missing rate ≤ 0.2) were used in GWAS for different traits in association population of 368 maize inbred lines. To minimize false positives, a mixed linear model (MLM) analysis was performed by a genomic association tool TASSEL v5 program (Bradbury et al. 2007) with P3D commands and no compression. This analysis incorporated the kinship matrix (*K*) and the population structure (*Q*), where the first 2 principal components included as fixed effects and the matrix of simple matching coefficients used to build up the K matrix were both from a previous study (Fu et al. 2013). The threshold for significance was estimated to be approximately *P* = 3.37 × 10^−7^ (that is, 1/2,965,911) by the Bonferroni correction method (Audic and Claverie 1997). The pairwise *r^2^* was calculated between a significant SNP and the SNPs located within a 0.5-Mb window R package “snp.plotter”.

### McrBC-based DNA methylation assay and bisulphite analysis

McrBC-based DNA methylation assay and bisulphite analysis were conducted according to a previously published method with minor modification (Mao et al. 2015). Genomic DNA (gDNA) was extracted from 72 HAI embryos from Chang7-2 and Zheng58 1 seeds using CTAB method. Total of 1 *μ*g gDNA was digested for 16 h at 37 °C with 10 units of McrBC enzyme (Takara), followed by 20 min incubation at 65 °C to inactivate the enzyme. A parallel reaction, in which equal amounts of gDNA were placed in buffer without the enzyme, was used as a negative control. The gDNA was digested with 3 biological replicates with or without McrBC enzyme. The digested DNA was diluted 4 times, followed by qPCR (the primers sequence as shown in Supplementary Table S1). McrBC digestion at the *ZmPIMT1* gene was normalized to the reference gene maize *UBIQUITIN-2* and *ACTIN1* and then to the undigested control.

For locus-specific bisulphite sequencing analysis, total of 1 mg gDNA was treated with the EpiArt DNA Methylation Bisulfite Kit V2 (Vazyme, EM102). After bisulphite conversion, the treated DNA was amplified by PCR using 2 × EpiArt HS Taq Master Mix (Vazyme, EM202). The primers used in this assay were designed using MethPrimer 2.0 (http://www.urogene.org/methprimer/), all the primers sequence as shown in Supplementary Table S1. PCR amplified fragments were purified and cloned into TA/Blunt-Zero Cloning Kit (Vazyme, C601) for sequencing. At least 8 clones of each genotype were sequenced.

### Maize protoplast transformation and detection of luciferase activity

Preparation and transformation of maize protoplasts was performed following a published protocol (Gu et al. 2013).

### Accelerated aging (AA) treatment of seeds

The AA treatment for maize seeds was performed following a published method (Zhang et al. 2023). At least 150 maize seeds were incubated in a constant temperature and humidity incubator at 95% relative humidity at 45 °C for 4 d (*zmpimt1* mutant) or 7 d (*ZmPIMT1* overexpression lines). After AA treatment, the seeds were desiccated at room temperature for 24 h. For NAA (Without Accelerated Aging) treatment, at least 150 maize seeds were incubated in the room temperature as control. Seed germination was performed on wet filter paper in Petri dishes in a plastic box at 25 °C in darkness where the humidity was maintained with a damp towel. The number of seeds completing germination was counted every 12 h and the root length was measured after imbibition for 120 h.

The AA treatment for *Arabidopsis* seeds was performed following a published method with minor modification (Zhang et al. 2023). Accelerated aging of seeds was conducted in a plastic desiccator equipped with a computer fan bolted to the inside of the lid to provide air circulation. The lead for the fan exited through a hole in the lid that was sealed with putty and tape as were the bolt holes holding the fan on the inside of the lid. A stir bar placed in the bottom of the desiccator constantly agitated the saturated KCl suspension. The stir bar was activated by placing the desiccator on a stir plate inside the incubating oven held at 42 °C. The seeds were placed in open Eppendorf tubes in a plastic weigh boat on a perforated plastic shelf inside the desiccator that was covered with fine plastic screening to prevent splashes from the liquid below. Hygrothermochron iButtons (Embedded data systems, Inc., Lawrenceburg, KY, USA) were placed in the boats to capture temperature and relative humidity data throughout the accelerated aging.

Seeds were surface sterilized (2 min 70% EtOH, 20 min 5% hypochlorite, 0.02% Triton × 100, rinsed 5×in sterile ddH_2_O) before 3 replications (>70 seeds each) were plated on solid media (½ strength MS media, 2.5 mm MES pH 5.8, 1% w/v agarose). Plates were scanned every 12 h for a week. The completion of germination was calculated for each time point and these numbers averaged and the standard error calculated and graphed. A 2 tailed ANOVA (alpha = 0.05) was used to assess whether there was statistically significant difference among means and Dunnett's test was subsequently used with WT Col as the control and each of the *atpabp* single or double mutants compared to it.

Following the germination trial, plates were placed at 3 °C for 3 d before being incubated at 22 °C under constant illumination for a further week to assess seed dormancy. Any seeds that had failed to complete germination after this treatment were recovered from the media and placed in tetrazolium chloride solution (1% w/v 2,3,5-triphenyl-2H-tetrazolium chloride in 0.1 m potassium phosphate buffer, pH 7.0), and incubated at 37 °C for 2 d before being assessed for live (red) or dead (unchanged color) status (Verma et al. 2013). Seed dormancy of WT and *atpapb2,8* was assessed following 42 d dry after-ripening by plating 2 sets of 3 replications of >60 surface sterilized seeds each from both genotypes on solid half strength MS media. One set was moist chilled as described above and the other set was not before the 1-week germination assay.

### Phylogenetic analysis

We reconstructed the phylogenetic relationships of ZmPABP2 (Zm00001d005276) with 19 additional PABP homologs through sequence alignment using ClustalW. The evolutionary history was inferred via the Neighbor-Joining method (Saitou and Nei 1987), with bootstrap values calculated from 1,000 replicates. Branches supported by less than 50% of bootstrap replicates were collapsed to reflect phylogenetic uncertainty. Bootstrap percentages indicating the frequency of co-clustering taxa are displayed adjacent to the corresponding nodes. Evolutionary distances were calculated using the Poisson correction model, expressed as the number of amino acid substitutions per site. This analysis included 20 protein sequences, with all ambiguous positions (containing gaps or missing data) excluded from the final dataset. The aligned sequences comprised 337 conserved amino acid sites. Phylogenetic computations were performed using MEGA7 software (Kumar et al. 2016). The sequence alignment files are provided as Supplementary materials under the title “PABP Sequence Alignments (FASTA Files)”.

### Preparation of samples and liquid chromatography tandem mass spectrometry (LC–MS/MS) analysis

To identify the target proteins of ZmPIMT1 in maize embryos, co-immunoprecipitation (Co-IP), trypsin digestion of eluted proteins, and liquid chromatography-tandem mass spectrometry (LC-MS/MS) were performed following a previously published method (Zhang et al. 2023). Twenty W22 maize seeds were germinated at 25 °C for 16 h and then subjected to a 42 °C heat shock for 2 h. The separated embryos (approximately 0.3 g) were ground in liquid nitrogen and homogenized in 500 *μ*L extraction buffer (50 mm Tris–HCl, pH 8.0, 150 mm NaCl, 10 mm MgCl_2_, 5 mm EDTA pH 8.0, 1% Triton X-100, 10% glycerin, and 1 mm PMSF). The mixture was then centrifuged at 13,000 ×g for 20 min at 4 °C. Then, 200 *μ*L of extracted protein (approximately 2 mg) from maize embryos was used to enrich ZmPIMT1 interacting proteins. ZmPIMT1-specific antibodies were used, and the manual of the BeaverBeads Protein A/G Immunoprecipitation Kit (Beaver, Suzhou, Jiangsu, China) was followed to detect ZmPIMT1 interacting proteins. After Co-IP, approximately 30 *μ*g of the sample was digested with trypsin. The LC-MS/MS was performed according to a published protocol (Zhang et al. 2023). The MS/MS data were processed using PEAKS Studio 8.0 (Bioinformatics Solutions Inc.). Tandem mass spectra were blasted against UniProt *Zea mays* protein database (https://www.uniprot.org/proteomes/UP000007305).

To identify the isomerized Asn (also deamidated) and Asp in the ZmPABP2 protein published protocols were followed (Zhang et al. 2023). The LC-MS/MS data were processed using MaxQuant with the integrated Andromeda search engine (version 1.6.17.0), and peak lists were searched against the UniProt *Z. mays* protein database. The mass tolerance for precursor ions was set to 20 parts per million in the first search and 5 parts per million in the main search, and the mass tolerance for fragment ions was set to 0.02 Da. Carbamidomethylation of Cys was specified as a fixed modification. Deamidation of Asn and isomerization of Asp were specified as variable modifications.

### Subcellular localization, BiFC and luciferase complementation imaging (LCI) assay

Maize protoplast preparation, transformation, and culture were performed according to a previously published protocol (Gu et al. 2013). Subcellular localization and the BiFC assay followed a published protocol (Zhang et al. 2023). Protoplasts were examined 12 h after transfection using a spinning-disk confocal system (Revolution WD, Andor, UK) equipped with a 100×/1.44 N.A. oil immersion objective. The Argon laser was set to 30% power to detect YFP, mCherry, and chloroplast autofluorescence signals. Specifically, YFP was excited at 488 nm, mCherry at 561 nm, and chloroplast autofluorescence at 637 nm. All images were processed using ImageJ software. LCI assays were performed in *Nicotiana benthamiana* leaves using Agrobacterium infiltration, following a previously published method (Su et al. 2021; Zhang et al. 2023). Images of luminescence were captured by a low-light, cooled, charge-coupled device imaging apparatus (PlantView100, Guangzhou Biolight Biotechnology Co., Ltd.).

### Expression and purification of ZmPIMT1 and AtPABP2 for in vitro interaction

Please refer to the Supplementary supporting information (Supplementary method S3).

### Generation of cy5-labeled polyadenylated mRNA target

A 326 bp DNA template commencing with the *E. coli* T7 promoter (31 nucleotides (nt) plus 5 random nts 5' of the promoter) was designed from the terminus of the COVID19 virus (nucleotides 29,505 to 29,794), purchased from Integrated DNA Technologies (Coralville, Iowa, USA). For details of generation of the Cy5-labeled polyadenylated mRNA target please refer to the supporting information (Supplementary method S4).

### ZmPIMT1 activity assessment and AtPABP2 iso-asp formation verification

Please refer to the supporting information (Supplementary method S5)

### Assessment of ZmPIMT1 binding to poly(a) mRNA

Please refer to the supporting information (Supplementary method S6)

### Assessment of AtPABP2 binding to poly(a) mRNA with or without 3 h aging and with or without repair by ZmPIMT1

Due to the propensity of some AtPABP2 to denature when aged at 37 °C, 100 *µ*L of purified AtPABP2 (66 *µ*M) was incubated 3 h at 25 °C (aged). Thereafter, 6 (Table 1 or Supplementary Fig. S22 A-F), 65 *µ*L reactions were constructed with final AtPABP2 concentrations of 30.46 *µ*M in 100 mm HEPES pH 7.5. The reactions containing unaged (reactions Table 1 or Supplementary Fig. S22, A and B and C) or aged (reactions Table 1 or Supplementary Fig. S22, D and E and F) AtPABP2; and these were incubated for 2 h with the addition of water; 4.66 *µ*M ZmPIMT1, 270 *µ*M AdoMet or 4.66 *µ*M ZmPIMT1, 270 *µ*M AdoHcy. Following this treatment, 3 replications of 2-fold serial dilutions of each reaction were made by adding 20 *µ*L of each reaction in the first well of each row of 12 consecutive microtiter plate wells and transferring 10 *µ*L from it into the next well of the row which contained 10 *µ*L of 100 mm HEPES pH 7.5. The transfer and mixing by pipetting (20 times) was performed using a multichannel pipet. Ten µL from the last transfer (12th well) following mixing, was discarded. Ten µL of labeled poly(A) mRNA (final Cy5-labeled target poly(A) mRNA concentration of 89 nm) was added to each well and mixed by pipetting (20 times). The greatest concentration of AtPABP2 (ligand; well 1) was 15.2 *µ*M (ZmPIMT1 2.33 *µ*M when present; AdoMet/AdoHcy 135 *µ*M when present) and the 12th well was 7.42 nm AtPABP2 (ZmPIMT1 1.14 nm when present; AdoMet/AdoHcy 65.9 nm when present). Plates were centrifuged at 4,200 ×g for 5 min at 25 °C to remove bubbles, the plates sealed to guard against evaporative loss, and TRIC assays were performed using a Dianthus Pico with AtPABP2 as the ligand and poly(A) mRNA as the target.

### Binding curve processing using the Dianthus screening analysis software

Data were imported into *Dianthus* Screening Analysis software (v2.1.1) allowing assessment of binding affinities. Each data point was scrutinized for aggregation, outliers, fluorescence homogeneity, photobleaching anomalies, and X, Y, and Z scan anomalies. Those data that passed these assessments were used to construct binding curves from which normalized fluorescence values were calculated and these were used to establish the dissociation constant at each time point for each treatment. The raw Fnorm values for the points that had passed the screening analysis were extracted and used to generate proportion bound curves.

### mRNA sequencing and (ribosome-nascent chain complex) RNC-mRNA sequencing

Please refer to the supporting information (Supplementary method S7).

### Evolutionary analysis of the *ZmPIMT1* locus

Please refer to the supporting information (Supplementary method S8).

### Statistical analyses

The estimates of the dissociation constant for each treatment and assay duration were assessed for statistically significant differences using ANOVA (α = 0.05). The *K_D_*s of aged or unaged AtPABP2 for poly(A) mRNA were compared at each time point of the assay. The same comparison was made of aged or unaged AtPABP2 for poly(A) mRNA in the presence of ZmPIMT1 with AdoMet or with AdoHcy. The *K_D_*s of AtPABP2 that were in the presence of ZmPIMT1 with AdoMet or AdoHcy were compared with the *K_D_* of AtPABP2 without ZmPIMT1 (the control) using Dunnett's post hoc mean separation test for both aged and unaged AtPABP2 for each time point of the assay. To assess the variability of the *K_D_* estimates for aged AtPABP2, 2 of the 3 replications were resampled. Averages and standard errors of the *K_D_*s from 2 of 3 replications were generated to provide 3 estimates of the standard error from Rep 1 and 2; 2 and 3; and 1 and 3. These 3 standard error estimates were then subjected to ANOVA (α = 0.05). The statistical results are shown in Supplementary Data Set S1.

### Accession numbers

Sequence data from this article can be found in the GenBank under accession numbers: *ZmPIMT1* (Zm00001d049966), *ZmPIMT2* (Zm00001d003275), *ZmPABP2* (Zm00001d005276), *Ubi-2* (Zm00001d053834), *Actin1* (Zm00001d010159), *AtPABP2* (At4G34110), *AtPABP4* (At2G23350), and *AtPABP8* (At1G49760).

## Supplementary Material

koaf217_Supplementary_Data

## Data Availability

The data underlying this article are available in the article and in its online supplementary material.

## Supplementary data

The following materials are available in the online version of this article.

**Supplementary Figure S1.** PCR identification of the promoter type of *ZmPIMT1* in Zhengdan 958 RIL (method of generation outlined in Fig. 1H) population lines.

**Supplementary Figure S2.** The seed morphology of Zhengdan 958 RIL population.

**Supplementary Figure S3.** PCR identification of the promoter variation of *ZmPIMT1* in maize inbred lines.

**Supplementary Figure S4.** Sequence alignment of the *ZmPIMT1* promoters of Hap C7-2 and Hap Z58 genotypes.

**Supplementary Figure S5.** PCR identification of the haplotype of ZmPIMT1 in 20 inbred lines.

**Supplementary Figure S6.** The mRNA expression and protein accumulation of ZmPIMT1 during W22 seed maturation and seed imbibition.

**Supplementary Figure S7.** Identification of the *zmpimt1* knock down mutant line.

**Supplementary Figure S8.** Sequence alignment confirmed the mutator insertion site of *zmpimt* mutant.

**Supplementary Figure S9.** Molecular characterization of *ZmPIMT1* overexpressing lines.

**Supplementary Figure S10.**

**Supplementary Figure S11.** Specific peptides of ZmPIMT1 were identified in LC-MS/MS assay after Co-IP.

**Supplementary Figure S12.** ZmPABP2 was identified as one of the target proteins by LC-MS/MS.

**Supplementary Figure S13.** A phylogenetic tree of relevant PABPs.

**Supplementary Figure S14.** The tissue expression profiles of relevant PABPs in maize and *Arabidopsis*, as reported in ePlants (respectively, https://bar.utoronto.ca/efp_maize and https://bar.utoronto.ca/efp/).

**Supplementary Figure S15.** Schematic representation of the expression vectors that were used in the luciferase complementation imaging (LCI) assay.

**Supplementary Figure S16.** The seed vigor of *atpabp2*, *atpabp4 and atpabp8* single mutant seeds was decreased after natural aging.

**Supplementary Figure S17.** The completion of germination of WT and *atpapb2,8* was statistically indistinguishable with or without moist chilling after 42 d dry after ripening.

**Supplementary Figure S18.** Prokaryotic expression of AtPABP2 in bacteria.

**Supplementary Figure S19.** Comparison of specific activity of ZmPIMT1 substrates.

**Supplementary Figure S20.** ZmPIMT1 does not bind poly(A) mRNA.

**Supplementary Figure S21.** The raw well scans and TRIC traces of aged AtPABP2 after 1 and 4 h incubation.

**Supplementary Figure S22.** Detection of poly(A) mRNA binding affinity of AtPABP2.

**Supplementary Figure S23.** Circular plot of gene ontology (GO) enrichment analysis for differentially expressed genes (DEGs).

**Supplementary Figure S24.** The subcellular localization of ZmPIMT1.

**Supplementary Figure S25.** Identification of ZmPABP2 post-translational modification sites by Co-IP and by LS-MS/MS in *zmpimt1* seed.

**Supplementary Figure S26.** Comparison of the accumulation of isoAsp in ZmPABP2 between *zmpimt1* and its NS control under heat stress.

**Supplementary Figure S27.** Depiction of the alignment of the maize ZmPABP2 and *Arabidopsis* AtPABP2 proteins.

**Supplementary Table S1.** The list of primers used in this study.

**Supplementary Table S2.** The listed proteins were identified using Co-IP, tryptic digestion of eluted proteins, and subsequent LC-MS/MS.

**Supplementary Table S3.** Average (± standard errors) of the standard errors of the KDs for aged AtPABP2, following 16 h incubation, binding poly(A) RNA.

**Supplementary Table S4.** RNA sequencing (RNA-seq) and Ribosome Nascent-chain Complex sequencing (RNC-seq) analysis of gene expression in normal seeds (NS) and zmpimt1 mutant seeds following 6 days of accelerated aging treatment.

**Supplementary Data Set 1.** Summary of statistical analyses.

**Supplementary File 1.** PABP Sequence Alignments.

**Supplementary method 1.** Vector Construction.

**Supplementary method 2.** ZmPIMT1 and ZmPABP2 Antibody Preparation, Protein Extraction, and Western Blot.

**Supplementary method 3.** Expression and Purification of ZmPIMT1 and AtPABP2.

**Supplementary method 4.** Generation of Cy5-labeled Polyadenylated mRNA Target.

**Supplementary method 5.** ZmPIMT1 activity assessment and AtPABP2 iso-Asp formation verification.

**Supplementary method 6.** Assessment of ZmPIMT1 binding to poly(A) mRNA.

**Supplementary method 7.** mRNA Sequencing and Ribosome-Nascent Chain Complex (RNC)-Bound mRNA Sequencing.

**Supplementary method 8.** Evolutionary analysis of the ZmPIMT1 gene locus

## Dive Curated Terms

The following phenotypic, genotypic, and functional terms are of significance to the work described in this paper:

PIMT1 Gramene: AT3G48330
PIMT1 Araport: AT3G48330

PIMT2 Gramene: AT5G50240
PIMT2 Araport: AT5G50240

PAB2 Gramene: AT1G79210
PAB2 Araport: AT1G79210

PAB3 Gramene: AT1G22760
PAB3 Araport: AT1G22760

PAB4 Gramene: AT2G23350
PAB4 Araport: AT2G23350

PAB5 Gramene: AT1G71770
PAB5 Araport: AT1G71770

PAB7 Gramene: AT2G36660
PAB7 Araport: AT2G36660

PAB8 Gramene: AT1G49760
PAB8 Araport: AT1G49760

PAB1 Gramene: AT1G34140
PAB1 Araport: AT1G34140
