# Supplementary material for: Natural variation in the ZmPIMT1 promoter enhances seed aging tolerance by regulating PABP2 repair in maize
Source: Plant Cell. 2025 Sep 18;37(10):koaf217. doi: 10.1093/plcell/koaf217 (PMC12510314; doi:10.1093/plcell/koaf217)
Supplement: koaf217_Supplementary_Data [file koaf217_supplementary_data.zip › Supplementary methods S1 to S8.docx]

**Supplementary methods**

**Supplementary method S1: Vector Construction**

For the construction of the bacterial expression vectors, the full-length coding regions of *ZmPIMT1*, AtPABP2 (AT4G34110; a member of the class II family of PABP proteins [1]) and the truncated ZmPABP2 (AA^179^-AA^654^) were amplified and inserted into the pET-28a vector (Novagen) between *Bam*HI and *Hind*III sites (Primers shown in Supplementary Table S1).

For the construction of expression vectors used in maize transformation, the full-length coding region of *ZmPIMT1* was amplified using primers pTF-*ZmPIMT1*-F and pTF-*ZmPIMT1*-R (Supplementary Table S1) from the *ZmPIMT1* vector described above. The PCR products were purified and inserted between *Bam*HI and *Sac*I sites of the pTF101.1 plasmid under the control of the ubiquitin promoter [2].

For the construction of vectors used in *ZmPIMT1* subcellular localization experiments, the coding sequence of *ZmPIMT1*, ZmPABP2 and AtPABP2 without the stop codon were amplified using primers (shown in Supplementary Table S1) and inserted into the *Nhe*I and *Mlu*I sites of pGL3-YFP vector, respectively.

For the construction of vectors used in the bimolecular fluorescence complementation (BiFC) assay, the coding sequence of *ZmPIMT1* without the stop codon was amplified and inserted into the *Spe*I and *Xho*I sites of the pUC-SPYNE vector. The coding sequences of *ZmPABP2 or AtPABP2* without the stop codon were amplified using primers (shown in Supplementary Table S1) and inserted into the *Spe*I and *Xho*I sites of the pUC-SPYCE vector.

For the construction of vectors designed for the luciferase complementation imaging (LCI) assay in tobacco leaves. The coding sequence of *ZmPIMT1* without the stop codon was amplified, purified and inserted into the *Bam*HI and *Sal*I sites of the pCAMBIA1300-NLUC vector (Supplementary Figure S13). The coding sequence of *ZmPABP2* or *AtPABP2* without the stop codon was amplified, purified and ligated into the *Bam*HI and *Sal*I sites of the pCAMBIA1300- CLUC vector, respectively. All the primer sequences are listed in Supplementary Table S1.

For the construction of vectors used for the characterization of the *ZmPIMT1* promoter activity, the dual luciferase reporter vector, in which the *Renilla* (*Renilla reniformis*) *LUCIFERASE* (*Rluc*) reporter gene was driven by the promoter fragments of *ZmPIMT1* ^Hap^ ^C7-2^ and *ZmPIMT1* ^Hap^ ^Z58^, were constructed. The *ZmPIMT1* ^Hap^ ^C7-2^ and *ZmPIMT1* ^Hap Z58^ promoter fragments, as depicted in Supplementary Figure S4, were each amplified by PCR using gene-specific primers pPIMT1-F and pPIMT1-R with genomic DNA isolated from Chang7-2 and Zheng58 seedlings, respectively. The amplicons were purified and inserted into the *Sac*I and *Nhe*I sites of the dual luciferase expression vector [2].

**Supplementary method S2:** **ZmPIMT1 and ZmPABP2 Antibody Preparation, Protein Extraction, and Western Blot**

The ZmPIMT1-His6 fusion protein and truncated ZmPABP2-His6 fusion protein were expressed separately in the *E. coli* strain BL21 (DE3), and the expression was induced by IPTG. Each purified hexahistidine-tagged fusion protein was subsequently used to immunize individual rabbits (*Oryctolagus cuniculus*), following a previously established protocol [3].

Total protein extraction was conducted using a previously established protocol [4]. In brief, the prepared embryos (about 0.1 g) were ground in liquid nitrogen and homogenized in 200 μL extraction buffer (100 mM Tris-HCl pH 6.8, 1% SDS, 30% Sucrose, 1% Triton X-100 (v/v), 5 mM EDTA pH 8.0, 2% β-mercaptoethanol, 2 mM DTT, and 1 mM PMSF). The mixture was then centrifuged at 13,000 ×g for 20 min at 4 °C. The protein in the supernatants was quantified by the Bradford assay [5]. Western blot analysis of ZmPIMT1 protein accumulation in maize embryos, imbibing seed, and seedlings was performed following a published protocol [3].

**Supplementary method S3: Expression and Purification of ZmPIMT1 and AtPABP2**

pET28a plasmids carrying the *ZmPIMT1* or *AtPABP2* (AT4G34110) coding sequences were transformed into *E. coli* BL21(DE3) Rosetta and Arctic (DE3) RIL cell lines, respectively.

A single colony of the pET28a:*ZmPIMT1* cells was grown overnight at 37°C with agitation (250 rpm) in 3 mL LB with 50 µg•mL^-1^ kanamycin, 34 µg•mL^-1^ chloramphenicol, and 0.2% glucose. One-half ml of culture was used to inoculate 1 L of the same media for growth until the optical density (OD; 600 nm) of the culture reached 0.5 to 0.6. IPTG was added to the final concentration of 1 mM and the culture growth was continued for a further 3 h before centrifuging (~8000 ×g, 10 min, 10 °C). The pelleted cells were resuspended in 10 ml of 20 mM Tris, 0.5 M NaCl, pH 7, with 25 g/mL lysozyme before freezing. After multiple freeze-thaw (on ice) cycles, the lysed cells were treated overnight on ice with 1 µg/ml DNaseI. Cellular debris was removed by centrifuging for 20 min at 10 °C at ~20,000 ×g. The supernatant was gravity-fed through a pre-equilibrated (12 mM Tris, 0.3 M NaCl, pH 7), Ni-charged, Tris-carboxymethyl ethylene diamine silica-based resin column (Protino® Ni-TED 2000 column; Macherey-Nagel Inc., Bethlehem, PA, USA; the bed volume was 1 mL). After washing with 8 mL of equilibration buffer, protein was eluted in 1 ml fractions with 0.25 M imidazole in equilibration buffer; these were collected and flash frozen. Fractions with the greatest quantities of ZmPIMT1 were dialyzed in 6-8000 MWCO Spectra/Por® membrane (Spectrum Laboratories, Inc., Rancho Dominguez, CA, USA) against 1 L 0.1 M HEPES, 0.15 M NaCl pH 7.5, with two additional 1 L changes of buffer after 2 h for ~12 h stirring incubation. Protein concentrations were determined with the NanoDrop One micro-UV/visible spectrophotometer (ThermoFisher Scientific Inc., Wilmington, DE, USA), using the extinction coefficient from the ProtParam site (Gasteiger et al., 2005) at ExPasy after entering the tagged protein sequence.

A single colony of pET28-a:AtPABP2 cells was added to 20 mL Terrific broth (TB) with appropriate antibiotics and grown overnight at 37℃ with agitation (250 rpm). The culture was transferred to 1 L of TB in a baffled flask and grown at 37℃ without selection for 3 h. The culture was then shifted to ~11℃ for 10 min. After adding IPTG (final concentration 1 mM), growth was continued at 11℃ for a further 24 h. Cells were harvested (~8000 ×g, 10 min, 10℃) and resuspended in 20 mM Tris, 0.5 M NaCl, pH 7 with Sigma*Fast*™ Protease Inhibitor Cocktail (EDTA-free, S8830; Sigma-Aldrich®, Millipore Sigma, St. Louis, MO, USA) and subjected to multiple freeze-thaw (on ice) cycles. 50 µg DNaseI was added and the mixture incubated overnight on ice. Cellular debris was removed by centrifuging for 20 min at 10℃ at ~20,000 ×g. The resulting supernatant was gravity-fed twice through a pre-equilibrated Protino® Ni-TED 2000 column (Macherey-Nagel Inc.), as above. After washing with 8 ml of equilibration buffer, purified protein was eluted with 1 ml fractions of 0.25 M imidazole in equilibration buffer. Fractions with the greatest quantities of AtPABP2 were buffer exchanged into 12 mM Tris, 0.3 M NaCl, and 10 mM MgCl_2_ using a Microcon® YM-30 micron (Millipore Sigma). Protein concentrations were determined with the NanoDrop One micro-UV/visible spectrophotometer (Thermo Fisher Scientific Inc.) and quantified using the extinction coefficient calculated by ProtParam site at ExPasy.

**Supplementary method S4: Generation of Cy5-labeled Polyadenylated mRNA Target**

A 326 bp DNA template commencing with the *E. coli* T7 promoter (31 nucleotides (nt) plus five random nts 5' of the promoter) was designed from the terminus of the COVID19 virus (nucleotides 29505 to 29794), purchased from Integrated DNA Technologies (Coralville, Iowa, USA), and served as template in PCR reactions with a complementary 3'-primer containing 33 contiguous adenines (Primer 3, Supplementary Table S1). Amplification was on a 1/100 dilution of the purchased stock with Primer 1 and Primer 2 (Supplementary Table S1). Conditions were 95 °C 3 min; 40 cycles of 98 °C 1 min, 50 °C annealing 1 min; and 72 °C extension, 1 min. The template was polished for 10 min at 72 °C. Amplicons were produced using Phire™ Taq (New England Biolabs; Ipswich, MA, USA) and purified from 1% (w/v) agarose gels using a kit (Qiagen gel extraction kit, Qiagen, Munich, Germany). DNA yield was quantified (Nanodrop) and 500 ng of the poly(A)-containing template used in a 20 µL T7 transcription reaction (HiScribe, NEB; protocol for short templates) with Primer 1 and Primer 3 (Supplementary Table S1) to generate Cy5-labeled polyadenylated COVID19 poly(A) mRNA by incubating the reaction at 37 °C for 16 h. The reaction producing Cy5-labeled poly(A) COVID19 mRNA was altered from the company recommended protocol due to the lower than recommended concentration of Cy5-X-UTP (1 mM, ABP Bioscience, Beltsville, MD, USA). Instead of 5 µL of 10 mM labeled UTP, 12 µL of 1 mM Cy5-X-UTP was added along with 1 µL of unlabeled UTP (100 mM, from kit) in a 29 µL final reaction volume. All else was similar to the unlabeled reaction except for adjusting the input volumes to those recommended for a 29 rather than a 20 µL reaction. Following transcription, the reactions were treated with RNase-free DNase I (Ambion, Inc., Austin, TX, USA) for 30 min at 37 °C, and the DNase was deactivated according to the instructions with the kit. RNA was purified using a kit (Qiagen) and retrieved using two sequential 50 µL elutions in RNase-free water. Unlabeled RNA was quantified using a nanodrop at wavelengths permitting estimates of yield and purity. Labeled RNA was also quantified at 650 nM to estimate the degree of Cy5 labeling.

Dilutions of labeled mRNA (1/20 to 1/1000) were placed in microtiter plate wells (NanoTemper 384 well plate) and used to assess the signal intensity to bring it within range of the Dianthus Pico detector. Based on this estimate, labeled RNA was diluted with unlabeled and with 100 mM HEPES pH 7.5 to allow quantification of binding.

**Supplementary method S5: ZmPIMT1 activity assessment and AtPABP2 iso-Asp formation verification**

One µL of purified ZmPIMT1 (60.6 µM in 100 mM HEPES pH 7.5, 150 mM NaCl; 2 µM in reactions) was added to IsoAsp containing peptide (6 mM stock of Val-Tyr-Pro-(l-**isoAsp**)-His-Ala; 480 µM in reaction) or unaged or aged AtPABP2 (66 µM stock in 12 mM Tris pH 7.5, 300 mM NaCl, 10 mM MgCl_2_; 22 µM in reaction) or water (blank) in 100 mM HEPES reaction buffer pH 7.5 and a mixture of ^3^H-CH_3_(S-Adenosyl methionine; AdoMet) and unlabeled AdoMet (0.27627 nmole total in reaction with a specific activity of 1.96 x 10^6^ dpm/nmole) in 30 µL reactions. AtPABP2 was aged by incubating the protein at 37 °C for 3 h. Radioactivity released into the scintillant as MeOH was counted hourly using a liquid scintillation analyzer (Tri-Carb 2900TR, PerkinElmer, Waltham, MA, USA). Disintegrations per min were used to compare the amount of label released from isoAsp-containing peptide, aged- and unaged-AtPABP2 to the control (blank) using ANOVA followed by Dunnett’s test (α = 0.05; Statistical Analysis Systems, Cary, NC, USA).

**Supplementary method S6: Assessment of ZmPIMT1 binding to poly(A) mRNA**

Three replications of purified ZmPIMT1 were assessed, with or without the inclusion of 270 µM (final) of either AdoMet or S-adenosyl homocysteine (AdoHcy), all in 100 mM HEPES, pH 7.5. Serial dilutions of ZmPIMT1 were made in 12 consecutive microtiter plate wells in 100 mM HEPES pH 7.5 (see below). The greatest concentration of ZmPIMT1 (well 1) was 2.33 µM to the least (12^th^ well) of 0.57 nM. Cy-5 labeled poly(A) mRNA (133.5 nM) was added to each well. Temperature Related Intensity Change (TRIC) assays were performed in triplicate for ZmPIMT1 + AdoMet and ZmPIMT1 + AdoHyc using a Dianthus Pico (Nanotemper GmbH, Munich, Germany) with ZmPIMT1 as the ligand and poly(A) mRNA as the target. The potential ZmPIMT1 binding to poly(A) mRNA was monitored periodically over the course of 38 h, the planned duration of surveillance of ZmPIMT1 chaperoning of AtPABP2 poly(A) mRNA binding.

**Supplementary method S7: mRNA Sequencing and Ribosome-Nascent Chain Complex (RNC)-Bound mRNA Sequencing**

*zmpimt1* mutant and its NS control line seeds were treated with accelerated aging (AA) treatment (45℃, 95% relative humidity) for 6 d. After AA treatment, the seeds were desiccated at room temperature for 24 h. Then the embryos were separated after 12 h imbibition and immediately frozen in liquid nitrogen. About 0.1 g ground powder was dissolved in 400 µL of lysis buffer. The resuspended extracts were then immediately loaded onto a 1 M sucrose cushion, prepared in polysome buffer containing 0.1 U/ml Superase•In™ RNase inhibitor. Ribosomes were pelleted by centrifugation for 4 h at 204,000 ×g 70,000 rpm, 4℃ in a TLA-110 rotor. The liquid was removed and the pellet was resuspended in 570 µL 10 mM Tris (pH 7), followed by the immediate addition of 30 µL 20% SDS. The sample was heated to 65℃ and RNA was extracted using two rounds of acid phenol/chloroform followed by chloroform alone. RNA was precipitated from the aqueous phase by adding sodium acetate to a final concentration of 300 mM followed by at least one volume of isopropanol. Precipitation was carried out at -30℃ for 30 min and RNA was then pelleted by centrifugation for 30 min at 20,000 ×g at 4℃. The supernatant was discarded, the pellet was airdried, and the RNA was resuspended in 150 µL Tris (pH 7). The typical RNA yield was 10 to 20 µg. Strand-specific library construction and sequencing was carried out by Gene Denovo Biotechnology Co. (Guangzhou, China). Volcano plot was generated from the experimental data using TBtools and RStudio for data visualization. Circos plot of gene ontology (GO) enrichment was generated from the experimental data using Chiplot (<https://www.chiplot.online/#BioPlot>).

**Supplementary method S8: Evolutionary analysis of the *ZmPIMT1* gene locus**

Nucleotide diversity (*θ_π_*) and (Fixation index) *F_ST_* was calculated using VCF tools (v0.1.13) [6]. These population parameters were estimated using the sliding-window approach with 3,000-bp windows and 100-bp increments; the whole genome was divided into 6,445,493 regions. Permutation test was performed by R to estimate the significance of *F*_ST_ during domestication. A total of 100,000 samples were randomly selected to construct the empirical distribution, then the significance of *F*_ST_ statistics of each region was calculated under this distribution. To assess whether the loss of genomic diversity in maize could be explained by the demography of maize domestication alone, coalescent simulations were performed by Hudson’s Ms program [7], with parameters based on the demographic history of maize domestication inferred by Beissinger *et al*. [8], and 10 000 coalescent simulations were repeated. The applied genotype data was from a published study [9].

**References**

1. Belostotsky DA: **Unexpected complexity of poly(A)-binding protein gene families in flowering plants: three conserved lineages that are at least 200 million years old and possible auto- and cross-regulation.** *Genetics* 2003, **163:**311-319.

2. Zhang Y, Song X, Zhang W, Liu F, Wang C, Liu Y, Dirk LMA, Downie AB, Zhao T: **Maize PIMT2 repairs damaged 3-METHYLCROTONYL COA CARBOXYLASE in mitochondria, affecting seed vigor.** *Plant J* 2023, **115:**220-235.

3. Li T, Zhang YM, Wang D, Liu Y, Dirk LMA, Goodman J, Downie AB, Wang JM, Wang GY, Zhao TY: **Regulation of Seed Vigor by Manipulation of Raffinose Family Oligosaccharides in Maize and Arabidopsis thaliana.** *Molecular Plant* 2017, **10:**1540-1555.

4. Gu L, Zhang YM, Zhang MS, Li T, Dirk LMA, Downie B, Zhao TY: **ZmGOLS2, a target of transcription factor ZmDREB2A, offers similar protection against abiotic stress as ZmDREB2A.** *Plant Molecular Biology* 2016, **90:**157-170.

5. Bradford MM: **A rapid and sensitive method for the quantitation of microgram quantities of protein utilizing the principle of protein-dye binding.** *Anal Biochem* 1976, **72:**248-254.

6. Danecek P, Auton A, Abecasis G, Albers CA, Banks E, DePristo MA, Handsaker RE, Lunter G, Marth GT, Sherry ST, et al: **The variant call format and VCFtools.** *Bioinformatics* 2011, **27:**2156-2158.

7. Hudson RR: **Generating samples under a Wright-Fisher neutral model of genetic variation.** *Bioinformatics* 2002, **18:**337-338.

8. Beissinger TM, Wang L, Crosby K, Durvasula A, Hufford MB, Ross-Ibarra J: **Recent demography drives changes in linked selection across the maize genome.** *Nat Plants* 2016, **2:**16084.

9. Bukowski R, Guo X, Lu Y, Zou C, He B, Rong Z, Wang B, Xu D, Yang B, Xie C, et al: **Construction of the third-generation Zea mays haplotype map.** *Gigascience* 2018, **7:**1-12.
