# Supplementary material for: Natural variation in the ZmPIMT1 promoter enhances seed aging tolerance by regulating PABP2 repair in maize
Source: Plant Cell. 2025 Sep 18;37(10):koaf217. doi: 10.1093/plcell/koaf217 (PMC12510314; doi:10.1093/plcell/koaf217)

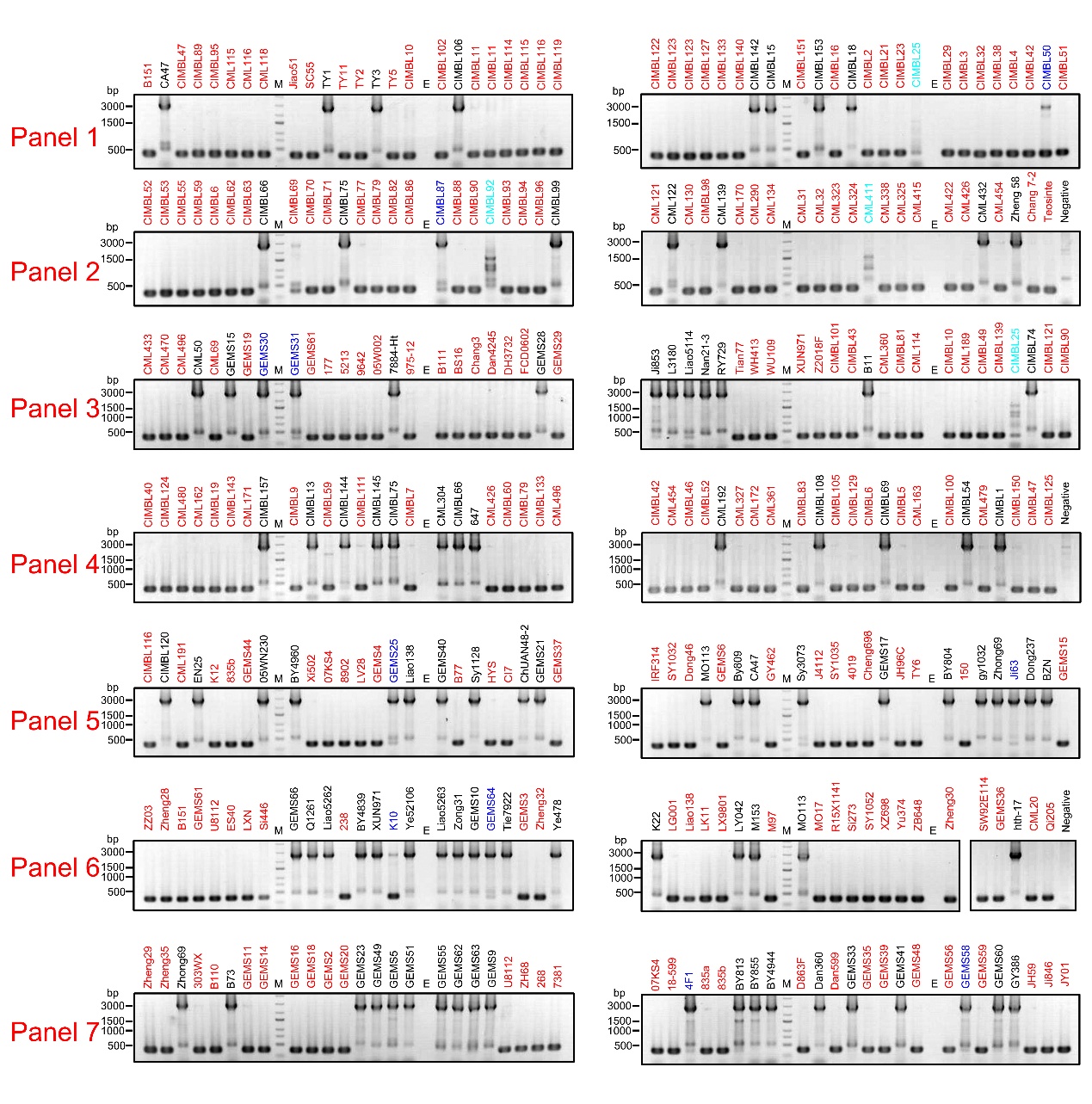


To be continued in the next page


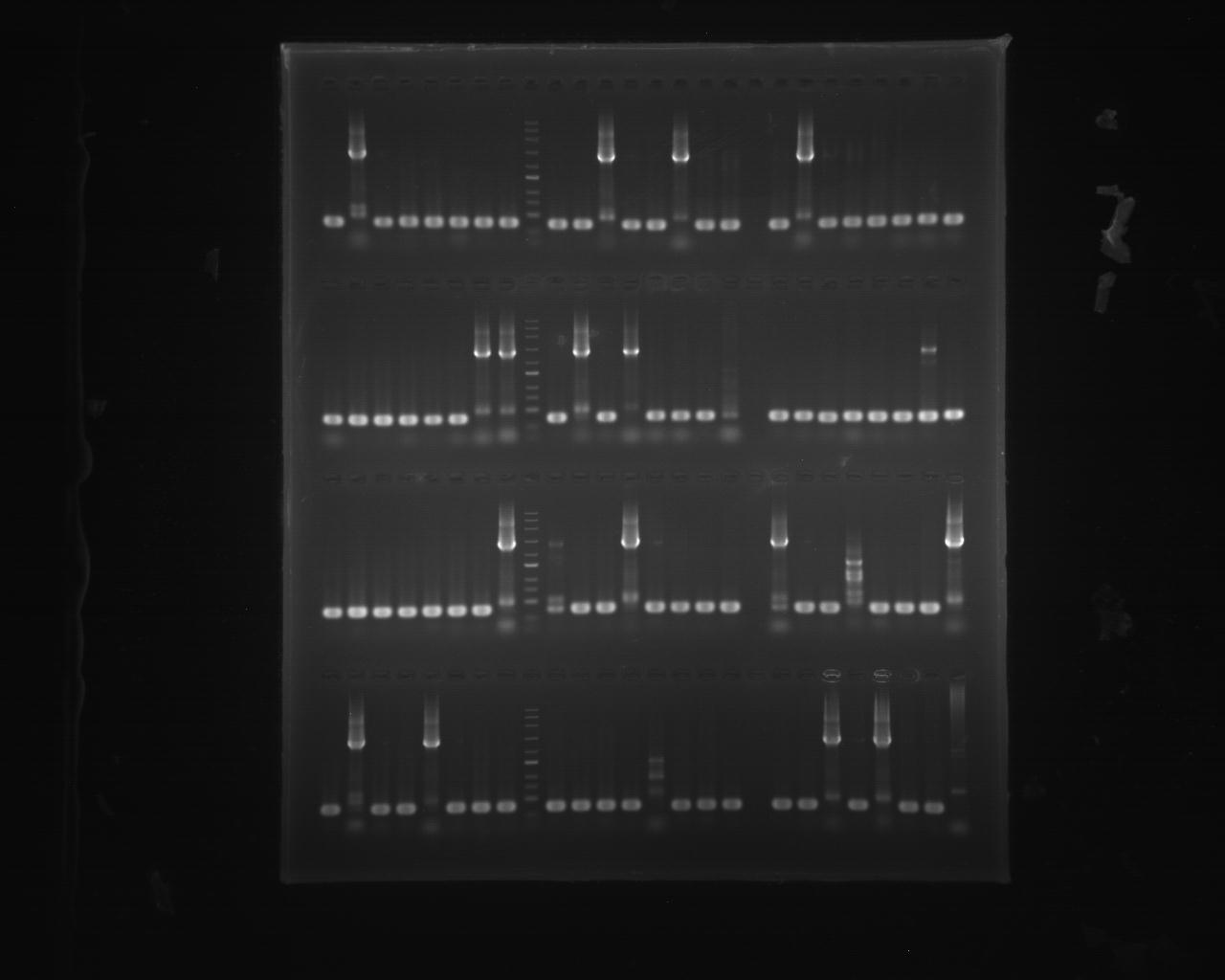


Panel 1 left

Panel 1 right

Panel 2 left

Panel 2 right


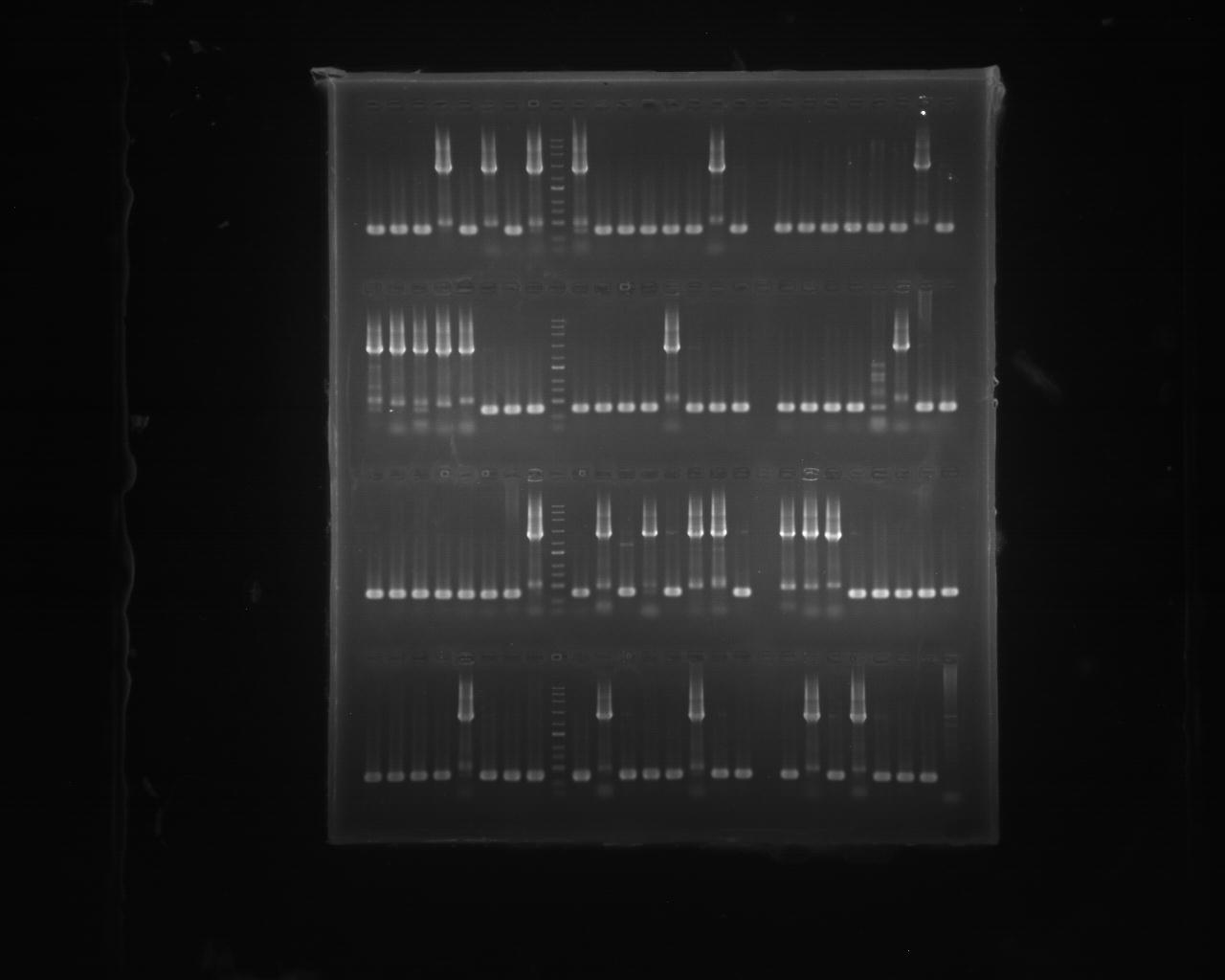


Panel 3 left

Panel 3 right

Panel 4 left

Panel 4 right


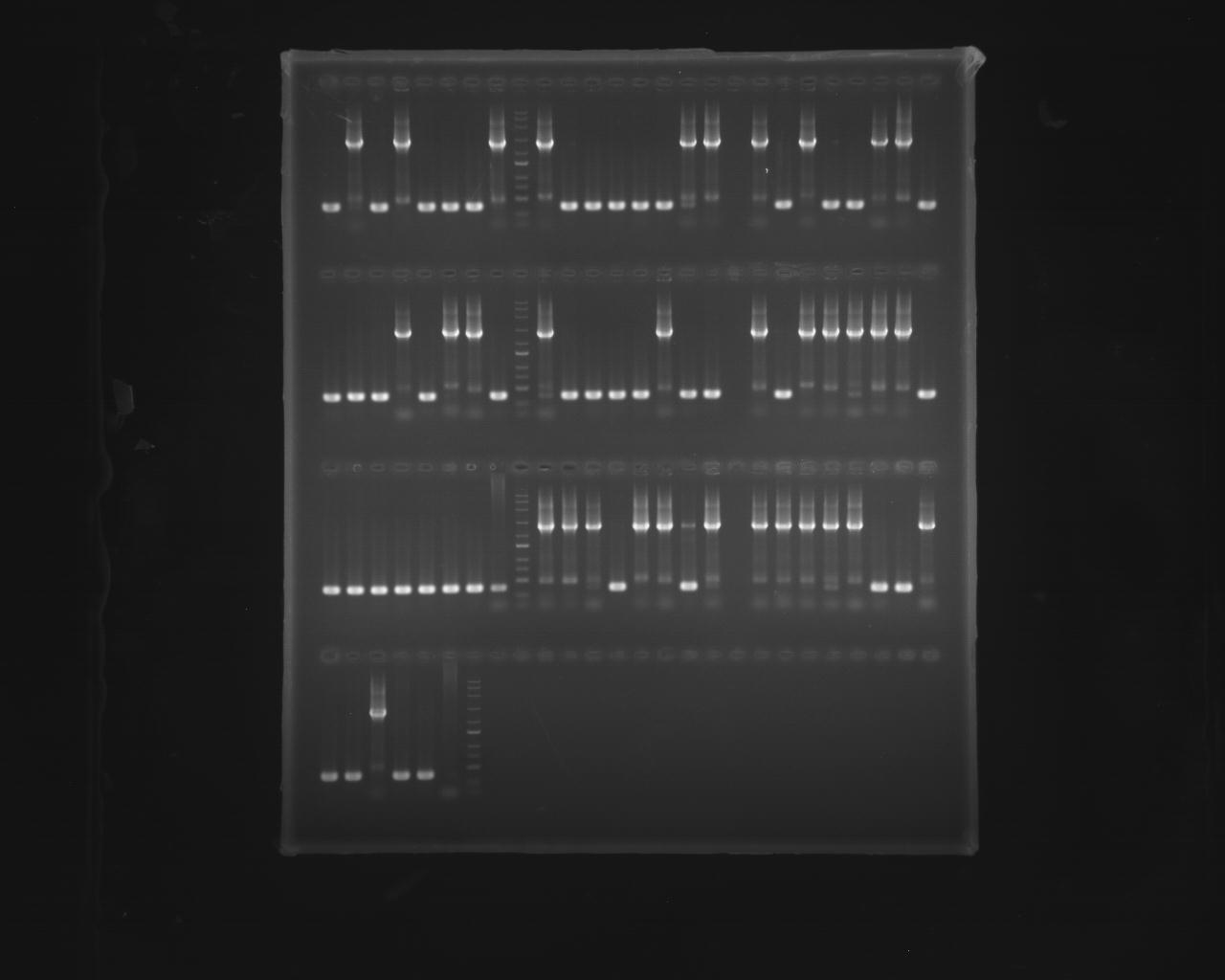


Panel 5 left

Panel 5 right

Panel 6 left

Panel 6 right


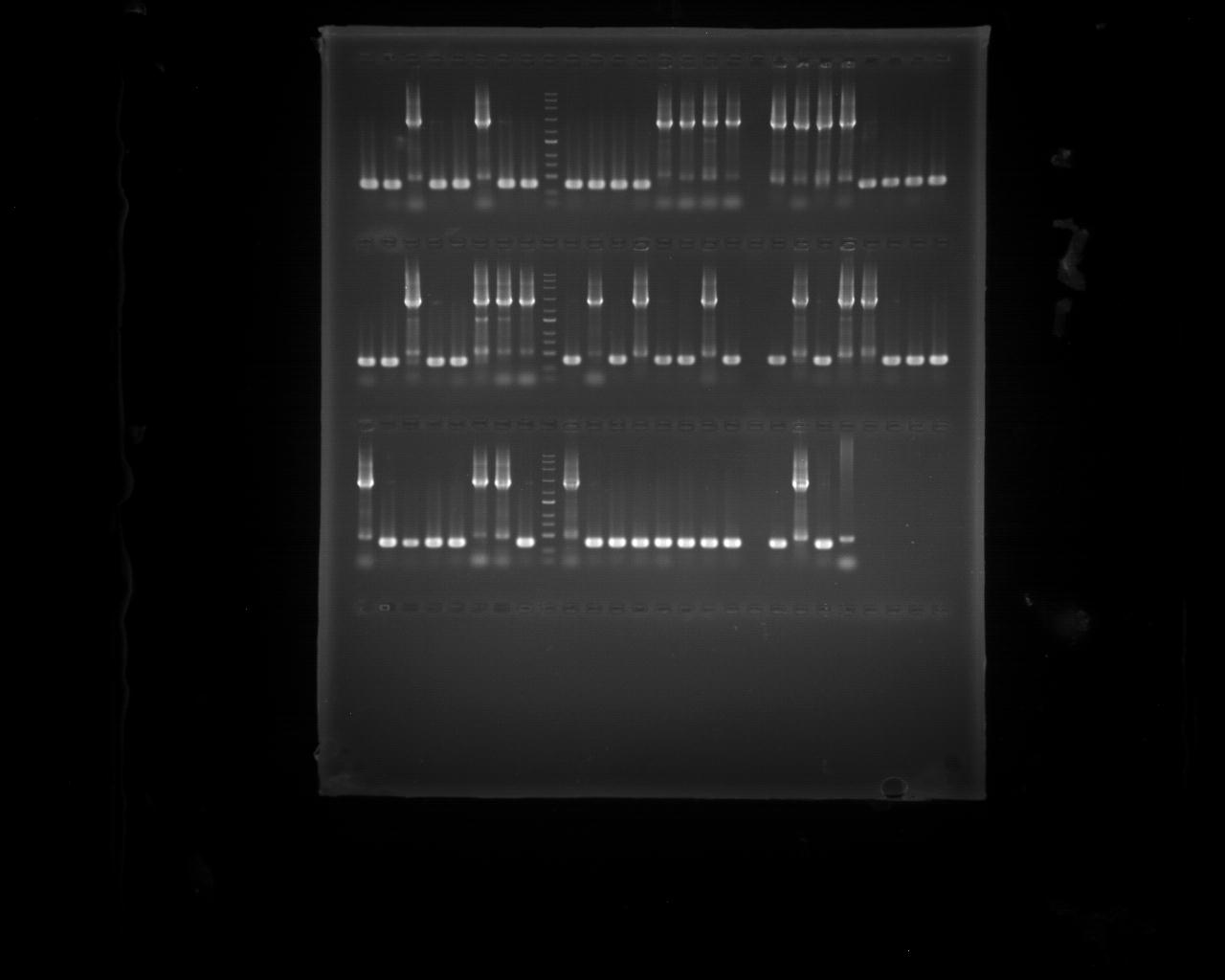


Panel 7 left

Panel 7 right

Panel 6 middle


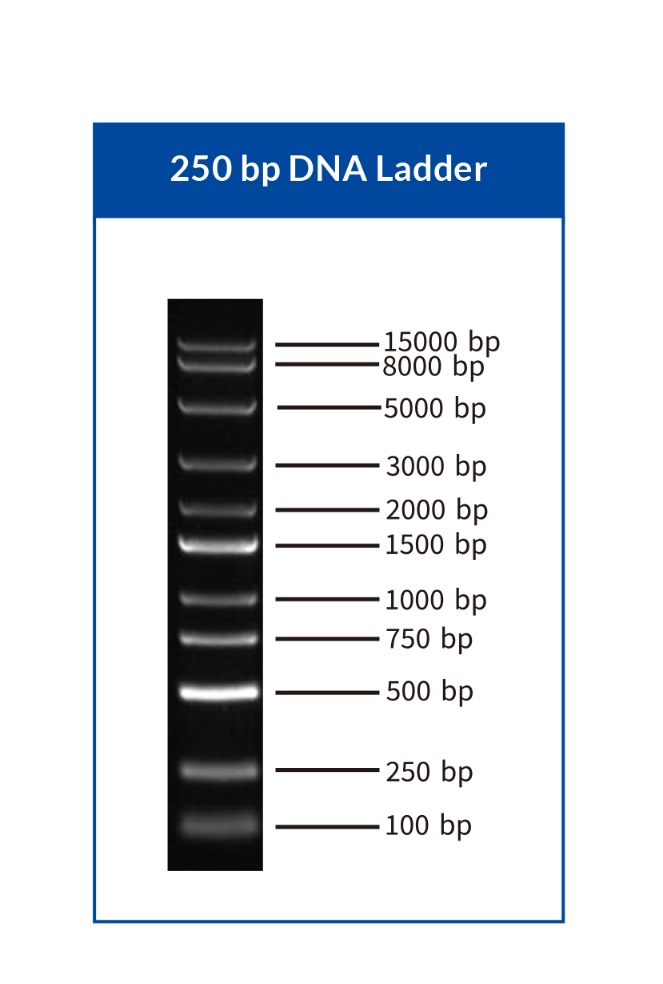

Supplement: koaf217_Supplementary_Data [file koaf217_supplementary_data.zip › Figure S3 source files/Figure S3 source files/Figure S3 source file description.docx]
