# Supplementary material for: Natural variation in the ZmPIMT1 promoter enhances seed aging tolerance by regulating PABP2 repair in maize
Source: Plant Cell. 2025 Sep 18;37(10):koaf217. doi: 10.1093/plcell/koaf217 (PMC12510314; doi:10.1093/plcell/koaf217)
Supplement: koaf217_Supplementary_Data [file koaf217_supplementary_data.zip › Supplementary Figures S1 to S27 revised.pdf]

Supplementary Figures

Natural Variation in the *ZmPIMT1* Promoter Enhances Seed Aging Tolerance by Regulating PABP2 Repair in Maize

Yumin Zhang[1], Lynnette M.A. Dirk[2], Jingliang Zheng[1], Jiahao Chai[1] Xianbo Song[1], Jie Cao[1], Hao Wang[1], Yan Liu[3], Yunjun Liu[3], Sihan Zhen[3], Junjie Fu[3], Guoji Wang[4], Shixiao Li[4], Arthur G. Hunt[5], A. Bruce Downie[2,*], Tianyong Zhao[1,*]

[1]State Key Laboratory of Crop Stress Resistance and High-Efficiency Production, College of Life Sciences, Northwest A&F University, Yangling, Shaanxi, 712100, China;

[2]Department of Horticulture, Seed Biology, Martin-Gatton College of Agriculture, Food and Environment, University of Kentucky, Lexington, KY 40546, USA;

[3]Institute of Crop Science, Chinese Academy of Agricultural Sciences, Beijing 100081, China;

[4]Gansu Wugu Seed Co., Ltd, Lanzhou, Gansu province, 730070, China;

[5]Department of Plant and Soil Sciences, Martin-Gatton College of Agriculture, Food and Environment, University of Kentucky, Lexington, KY 40546, USA.

***Corresponding authors:** A. Bruce Downie, adownie@uky.edu; Tianyong Zhao, tzhao2@nwafu.edu.cn.

Supplementary Figures

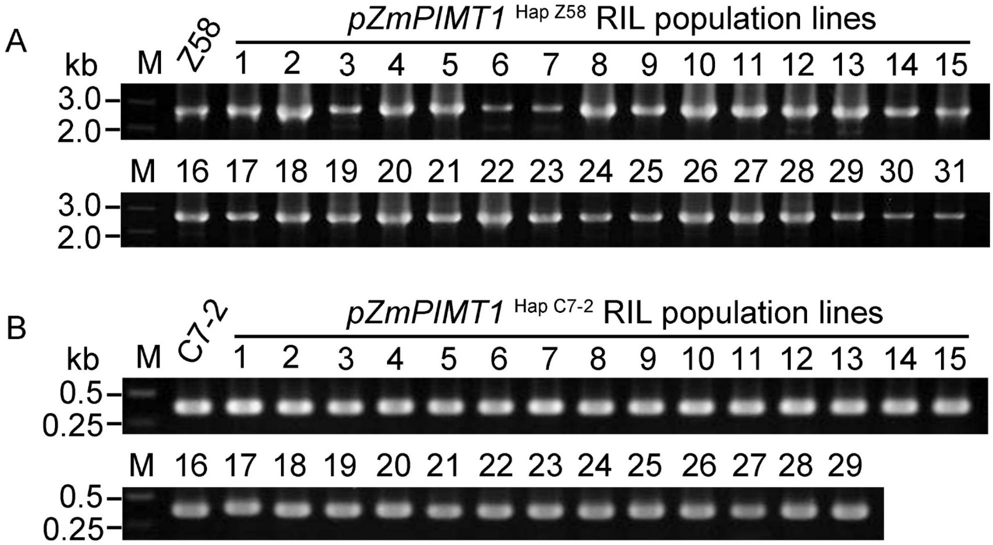

Supplementary Figure S1. PCR identification of the promoter type of *ZmPIMT1* in Zhengdan 958 RIL (method of generation outlined in Figure 1H) population lines. (Supports Figure 1).

(**A**) PCR identification of the Hap Z58 of *ZmPIMT1* in Zhengdan 958 RIL population lines. (**B**) PCR identification of Hap C7-2 of *ZmPIMT1* in Zhengdan 958 RIL population lines. RIL, Recombinant inbred line. Z58, Zheng58. C7-2, Chang7-2. Hap, Haplotype.

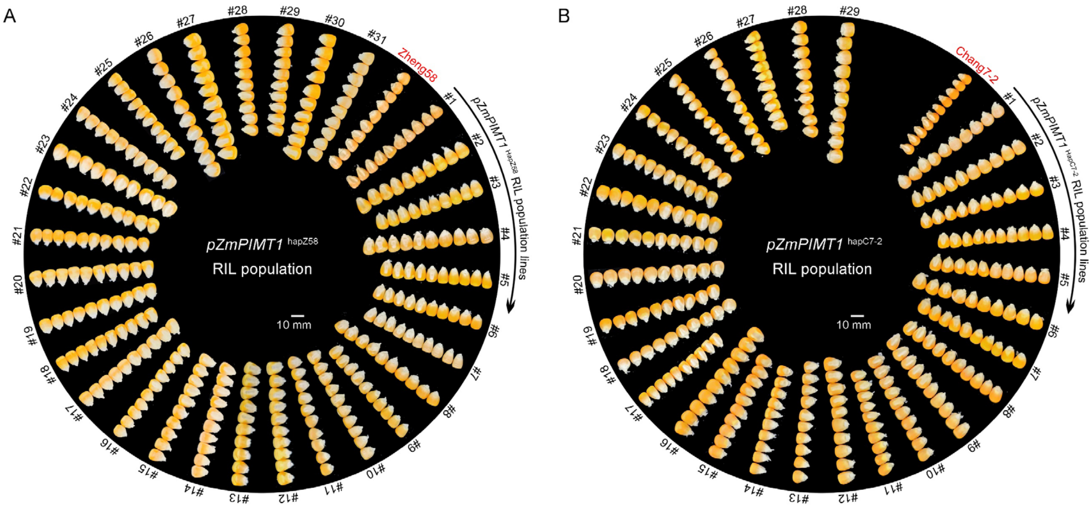

Supplementary Figure S2. The seed morphology of Zhengdan 958 RIL population. (Supports Figure 1).

(**A**) The seed morphology of *ZmPIMT1* $^{Hap\ Z58}$ RIL population. (**B**) The seed morphology of *ZmPIMT1* $^{Hap\ C7\text{-}2}$ RIL population. A total of 10 seeds, selected from the center of each cob, were photographed with 5 seed embryos facing up, 5 seed embryos facing down. Images were digitally extracted for comparison. Scale bar = 10 mm.

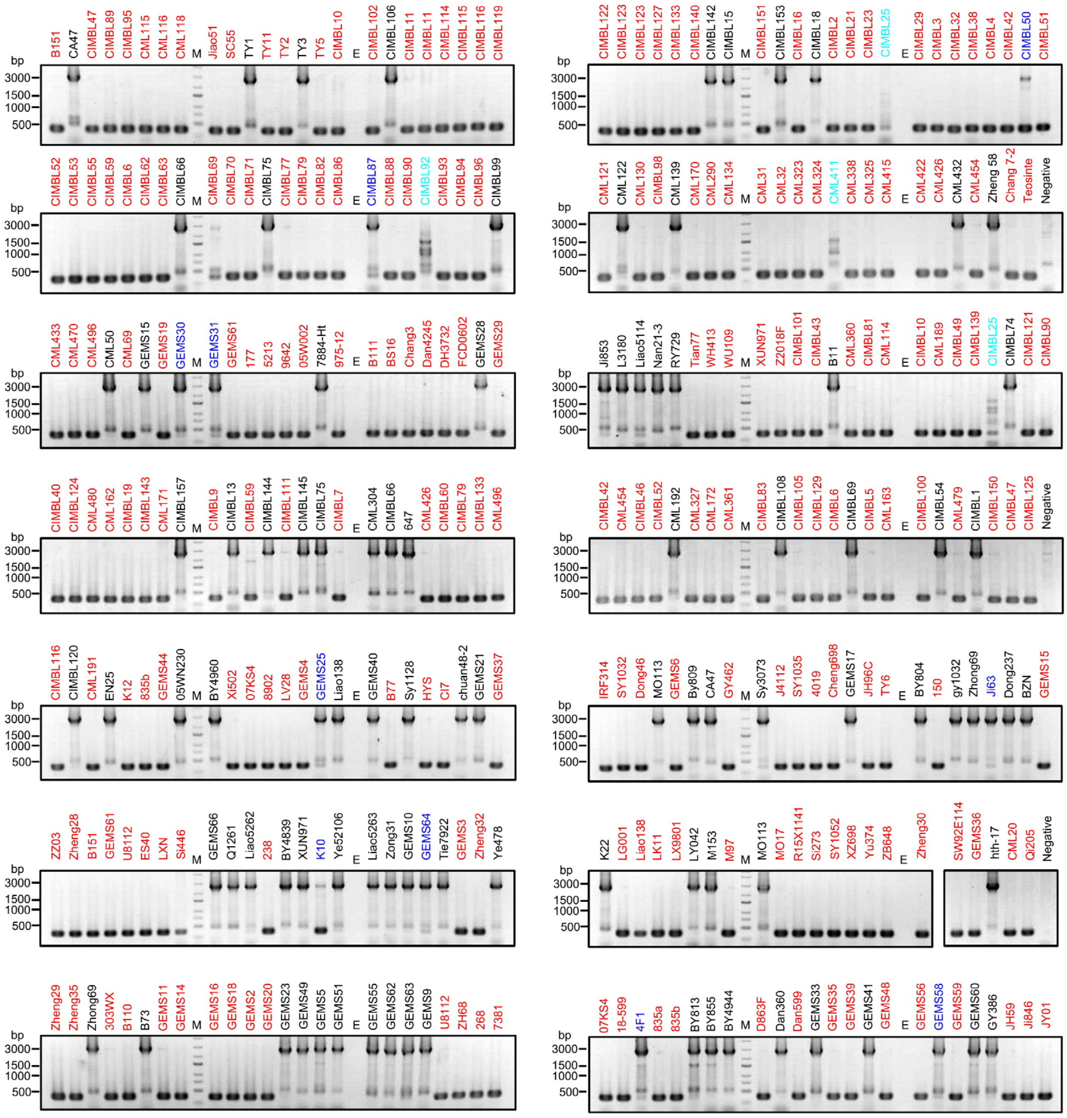

Supplementary Figure S3: PCR identification of the promoter variation of *ZmPIMT1* in maize inbred lines. (Supports Figure 2). (Primers, F4 and R4 sequences in Supplementary Table S1) were used with teosinte and the parental lines of the Zhengdan 958. The line names with red and black fonts represents the inbred line with the Hap C7-2 or Hap Z58, respectively. The line names in a blue font represents the inbred lines for which the haplotype cannot be distinguished. The line names in a cyan font represents the inbred lines for which the haplotype cannot be classified given neither size is present for the reaction. M: DNA marker, E: empty lane without loading PCR product.

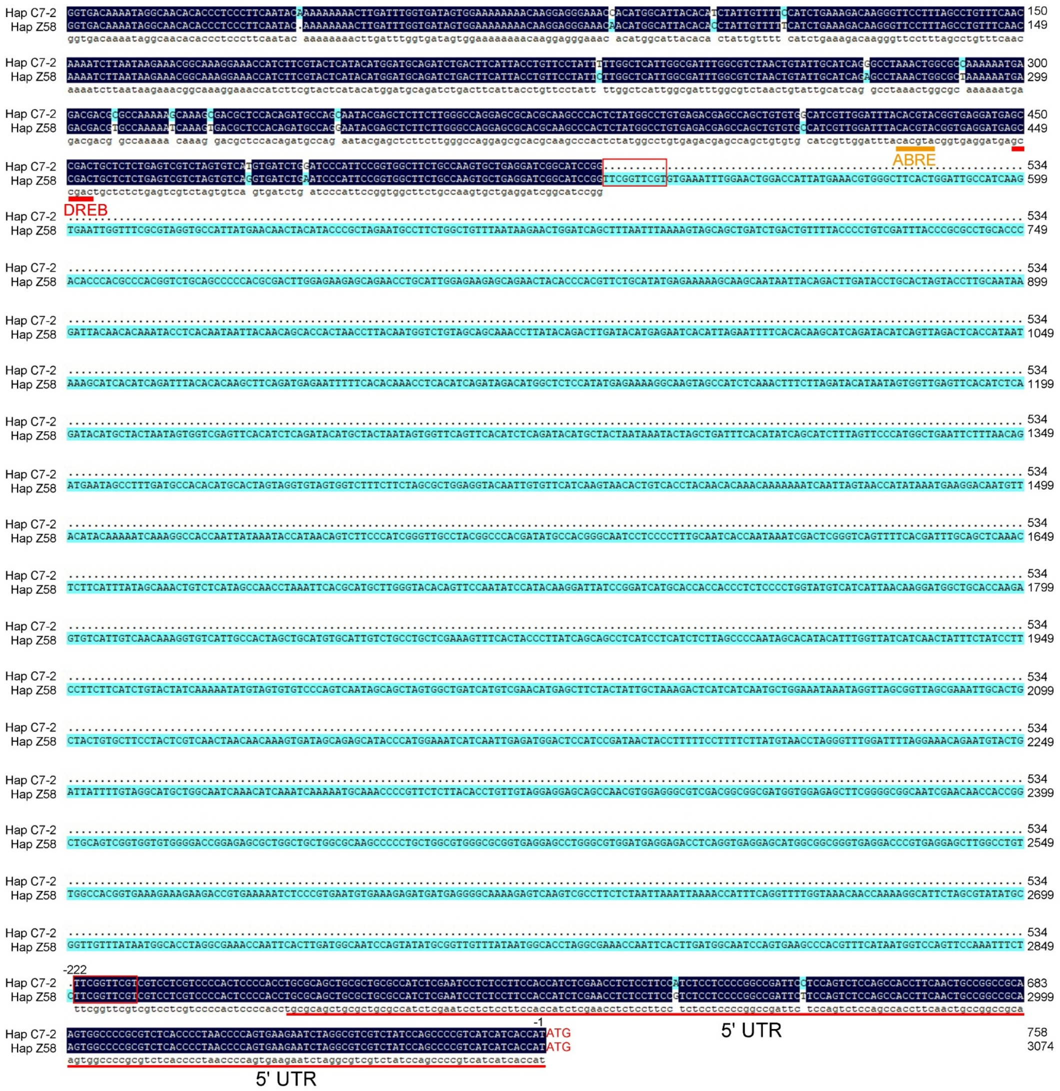

Supplementary Figure S4: Sequence alignment of the *ZmPIMT1* promoters of Hap C7-2 and Hap Z58 genotypes. (Supports Figure 2). The two red boxes indicate the 10 bp TIR (terminal inverted repeats) and the red line indicates the 5' UTR of *ZmPIMT1*. There are 15 polymorphisms (2 of which are in the 5' UTR) according to the BLAST sequence. The prediction of an ABRE element (orange) and a DREB element (red) is marked with different colors under the sequence. UTR, Untranslated Region. AREB, ABA-Responsive Element. DREB, Dehydration-Responsive Element. In the sequence alignment, identical residues are cyan. Blue indicates divergent residues or alignment gaps (e.g., in Hap C7-2).

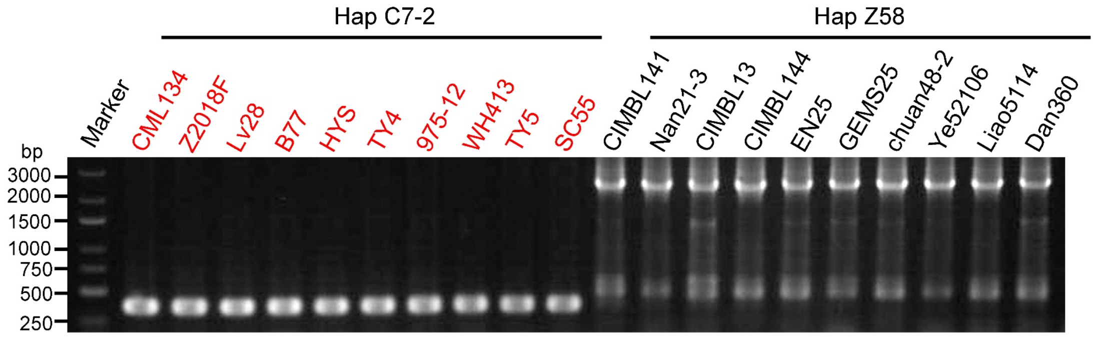

Supplementary Figure S5: PCR identification of the haplotype of *ZmPIMT1* in 20 inbred lines. (Supports Figure 2). The red font represents the inbred line with the Hap C7-2, and the black font represents the inbred line with the Hap Z58. (Primers, F4 and R4 sequences in Supplementary Table S1) were used. Z58, Zheng58. C7-2, Chang7-2. Hap, Haplotype.

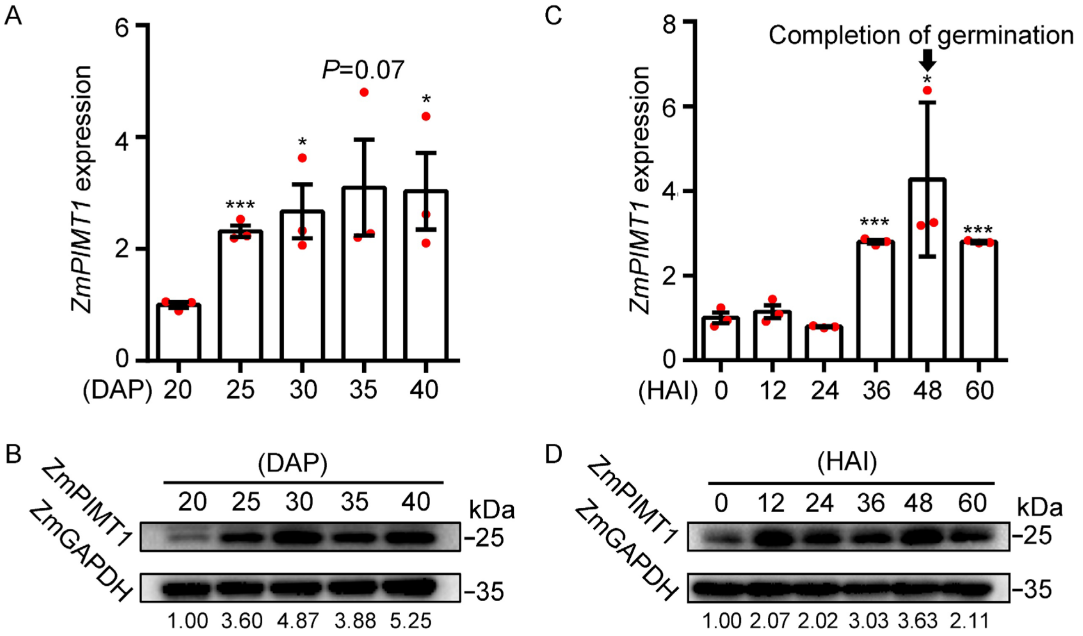

Supplementary Figure S6. The mRNA expression and protein accumulation of ZmPIMT1 during W22 seed maturation and seed imbibition. (Supports Figure 4).
(**A**) Real-time RT-PCR analysis of the *ZmPIMT1* mRNA accumulation in the embryo at 20, 25, 30, 35 and 40 d after pollination (DAP) during seed maturation. The *ZmPIMT1* mRNA amount was normalized to *ZmGAPDH* mRNA amounts in the respective tissues. Data are means ± SE (n = 3) tested with a Student's *t*-test (*denotes a $P < 0.05$, and ***denotes $P < 0.001$) as compared with the *ZmPIMT1* mRNA amount in the 20 DAP embryos. Primer sequences are in Supplementary Table S1.
(**B**) Western blot analysis of the ZmPIMT1 protein accumulation in maize embryos during seed maturation (DAP as in (A)). The western blot analysis of GAPDH protein (bottom panel) is used to demonstrate equal protein loading and to calculate the ZmPIMT1/ZmGAPDH signal intensity ratios (given below the bottom panel) The molecular mass (kDa) labels are from a lane with defined protein markers.

(**C**) Real-time RT-PCR analysis of the *ZmPIMT1* mRNA accumulation (as described in (A)) in the embryo at 0, 12, 24, 36 48 and 60 h after imbibition (HAI) during seed germination/seedling establishment. Data are means ± SE (n = 3)tested with a Student's *t*-test (*denotes a $P < 0.05$, and **denotes a $P < 0.01$) as compared with 0 HAI expression. The black arrow indicates seed had started to complete germination at 48 HAI.

(**D**) Western blot analysis of the ZmPIMT1 protein accumulation (as described in (B)) in maize embryos during seed germination/seedling establishment (HAI as in (C)).

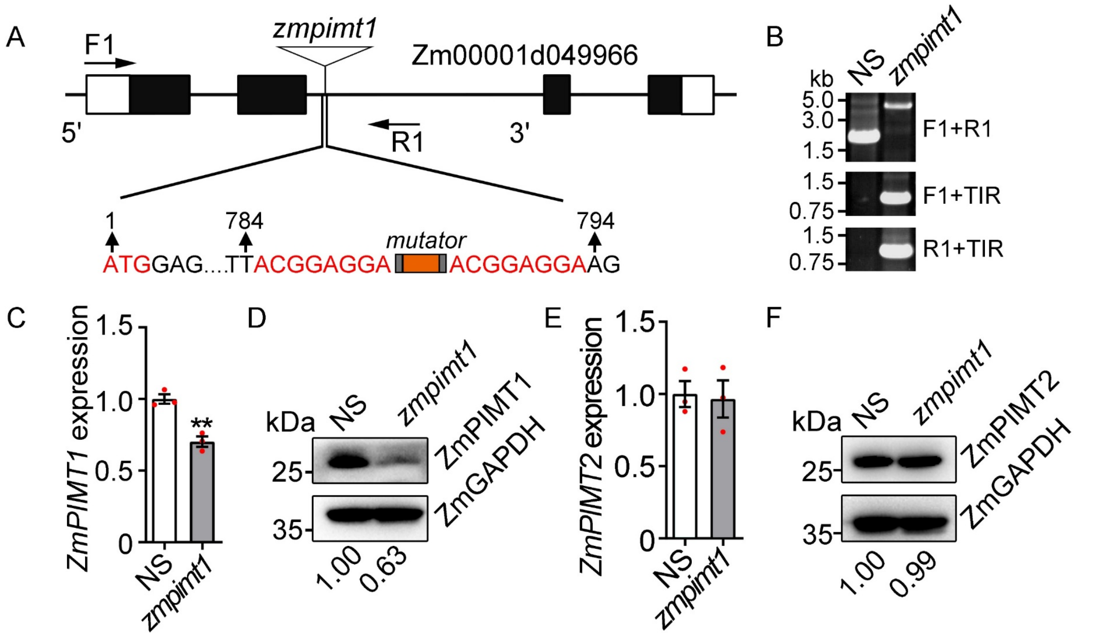

Supplementary Figure S7. Identification of the *zmpimt1* knock down mutant line. (Supports Figure 4).

(**A**) Structure of the *ZmPIMT1* gene and mutator insertion of the *zmpimt1* mutant. Exons are shown as black boxes and introns as lines. UTRs are shown as white boxes. The mutator insertion site shown above is further delineated below showing the typical 8 bp duplication. PCR gene-specific primer and the mutator terminal inverted repeat (TIR) primer binding sites are indicated and sequences are in Supplementary Table S1. The red “ATG” denotes the start codon, and the red “ACGGAGGA” sequence represents the 9-bp duplication caused by the insertion of the mutator transposon.

(**B**) PCR genotyping of the *zmpimt1* mutant and its null segregant (NS) plant. Using primers F1 and R1 (depicted in A), a 4.5-kb and a 1.8-kb DNA fragment was amplified from the *zmpimt1* mutant and NS plant, respectively, indicating that the mutator had inserted between the binding sites of these primers. TIR, terminal inverted repeat.

(**C, E**) Real-time RT-PCR analysis of the *ZmPIMT1* (**C**) and *ZmPIMT2* (**E**) expression in the 12 HAI embryo of NS and *zmpimt1* mutant. The expression of *ZmPIMT1* or *ZmPIMT2* was normalized to *ZmGAPDH* expression. Data are the means ± SE (n = 3) and the significance was determined using a Student's *t*-test (**$P < 0.01$).

(**D, F**) Western blot analysis of the ZmPIMT1 (**D**) and *ZmPIMT*2 (**F**) protein

accumulation in the NS and *zmpimt1* mutant 12 HAI embryo. The ZmGAPDH protein was used as a measure of equal protein loading and to calculate the ZmPIMT1/ZmGAPDH or ZmPIMT2/ZmGAPDH signal intensity ratios (given below the bottom panel).

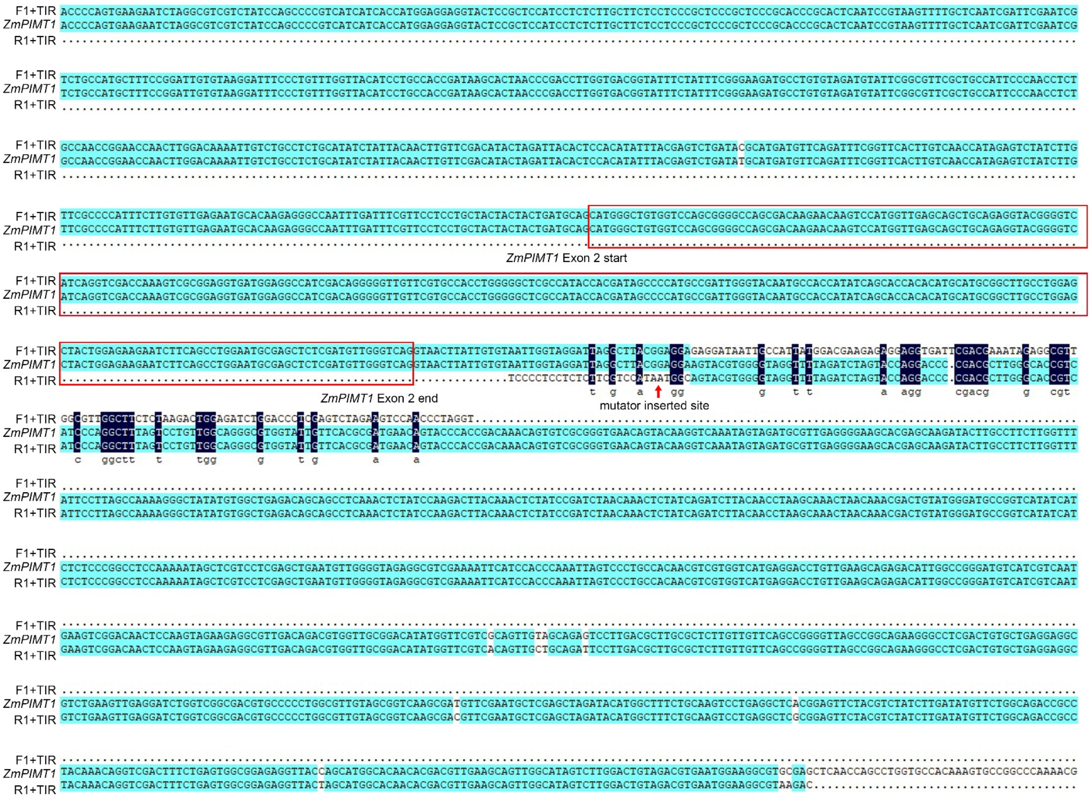

Supplementary Figure S8. Sequence alignment confirmed the mutator insertion site of *zmpimt* mutant. (Supports Figure 4). The red box indicates the second exon of *ZmPIMT1* and the red arrow indicates the mutator insertion site. In the sequence alignment, identical residues are cyan. Blue indicates divergent residues or alignment gaps (e.g., in F1+TIR).

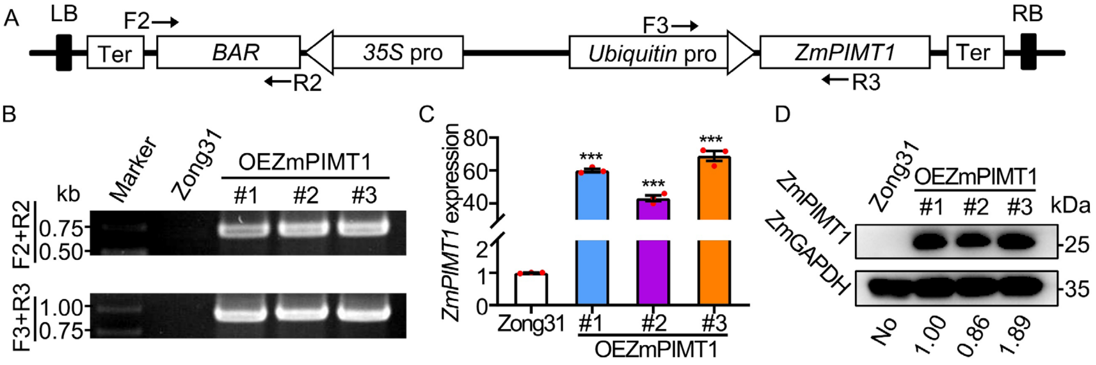

Supplementary Figure S9. Molecular characterization of *ZmPIMT1* overexpressing lines. (Supports Figure 4).

(**A**) Schematic representation of *ZmPIMT1* expression vector for maize transformation. The 35S promoter (*35S* pro) and *Ubiquitin* promoter (*Ubiquitin* pro) were used to direct the coding region of the Bialaphos resistance gene (*BAR*) and *ZmPIMT1*, respectively,

with a terminator (Ter) for both prior to the border sequences (RB, right border; and LB, left border). The binding position of PCR primers (F2, R2, F3 and R3; sequence in Supplementary Table S1) used in (B) is indicated.

(**B**) PCR confirmation of *ZmPIMT1*-transformed maize plants (OEZmPIMT1#1, #2 or #3) and the parental, non-transformed maize (Zong31) using primers as displayed in (A).

(**C**) Real-time RT-PCR analysis of the *ZmPIMT1* mRNA accumulation in 24 h after imbibition (HAI) maize embryos between transgenic maize lines and the non-transformed line, Zong31. The *ZmPIMT1* mRNA amount was normalized to that of *ZmGAPDH*. Data were the means ± SE (n = 3). Three asterisks denote a $P < 0.001$, as compared with the *ZmPIMT1* mRNA accumulation in the nontransformed line Zong31, as determined using Student's *t*-test.

(**D**) Western blot analysis of the ZmPIMT1 and ZmGAPDH protein accumulation (top and bottom panels, respectively) in the non-transformed Zong31 and transgenic 24 HAI maize embryos. The ZmGAPDH protein was used as a measure of equal protein loading and to calculate the ZmPIMT2/ZmGAPDH signal intensity ratios (given below the bottom panel).

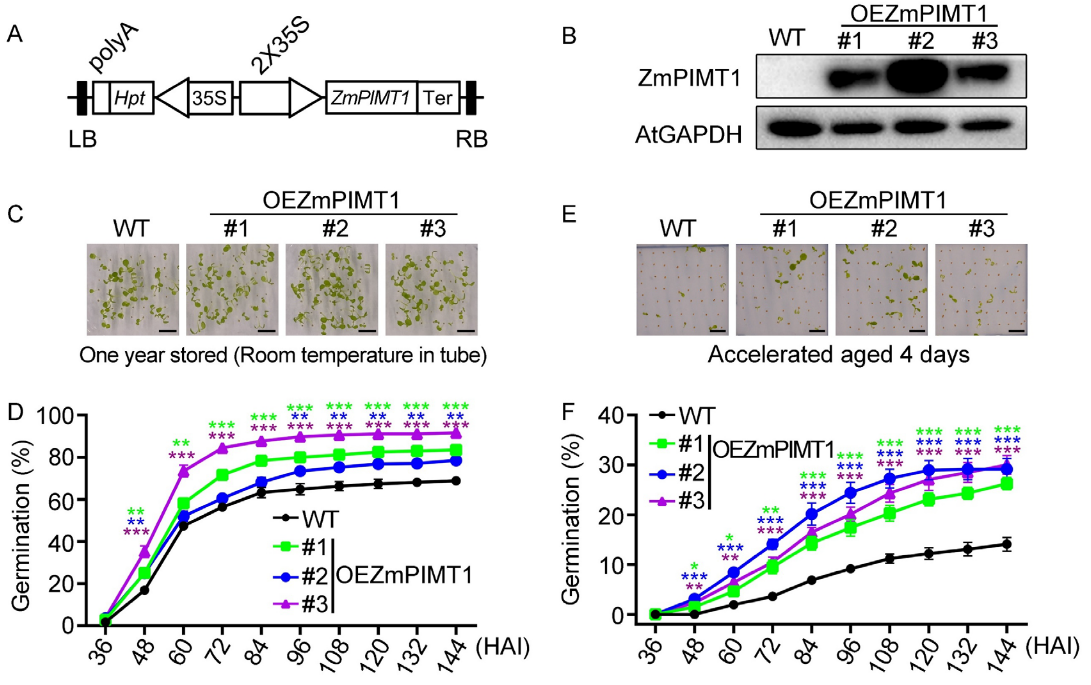

Supplementary Figure S10. Overexpression of *ZmPIMT1* in *Arabidopsis* increases seed vigor. (Supports Figure 4).

(**A**) Schematic representation of the expression vectors of *ZmPIMT1* used for *Arabidopsis* transformation. LB and RB represent a left and right border sequence, respectively. A single or double 35S promoter sequence is denoted with *35S* and *2x35S*, respectively. There is a terminator sequence (Ter) and a poly A signal sequence (polyA) as well as the hygromycin phosphotransferase gene (*Hpt*).

(**B**) Western blot analysis of ZmPIMT1 (top panel) and GAPDH (bottom panel) protein

levels in *Arabidopsis* leaves in the various transgenic *Arabidopsis* plants and the control plants. WT are untransformed Col-0 *Arabidopsis* plants; and OEZmPIMT1 #1, #2 or #3 are the three ZmPIMT1-overexpressing *Arabidopsis* plants.

(**C** and **E**) Representative photographs of the seeds/seedlings after 144 h of imbibition from the defined genotypes of *Arabidopsis* plants that were stored for one year at room temperature in a 10 mL tube (**C**) or after 4 d accelerated aging of the seed (AA4) (**E**). The black scale bar represents 5 mm.

(**D** and **F**) Comparison of seed germination percentages with time (h after imbibition (HAI)) between WT and the defined genotypes of *Arabidopsis thaliana* that were stored for one year (**D**) or following AA4 (**F**) as newly harvested seed. There were eight replicates for each treatment and there were 50 seeds for each replicate. A Student's *t* test was conducted; and *, **, and *** denote a *P-value of* < 0.05, < 0.01, and < 0.001, respectively relative to WT within the same aging treatment. Note in **F** that the Y-axis scale is maximized to only 40%.

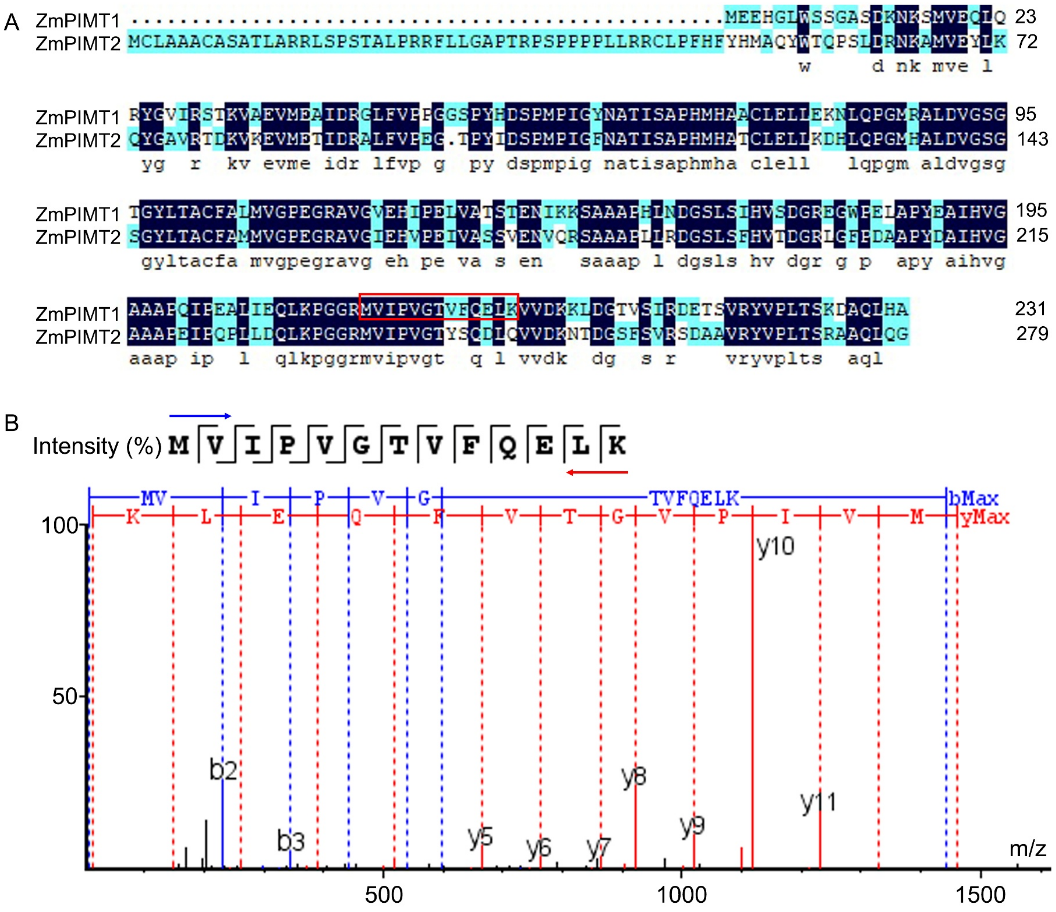

Supplementary Figure S11. Specific peptides of ZmPIMT1 were identified in LC-MS/MS assay after Co-IP. (Supports Figure 5).

(**A**) Protein sequence alignment between ZmPIMT1 and ZmPIMT2 with red box indicating the specific peptides of ZmPIMT1, which were identified by the LC-MS/MS assay after Co-IP. Identical and divergent residues are colored cyan and blue, respectively.

(**B**) Electrospray ionization mass spectra of ZmPIMT1 identified peptide, MVIPVGTVFQELK. The proteins were PAGE purified and in-gel digested with trypsin, and the resulting peptides were extracted and analyzed by EASY-nLC 1000 UPLC (Thermo Fisher) and Orbitrap Fusion Lumos (Thermo Fisher). The fragmentation occurs at peptide bonds to generate b ions (N-terminal fragments) and y ions (C-terminal fragments) at specific m/z ratios and intensities that provide information regarding amino acid sequence.

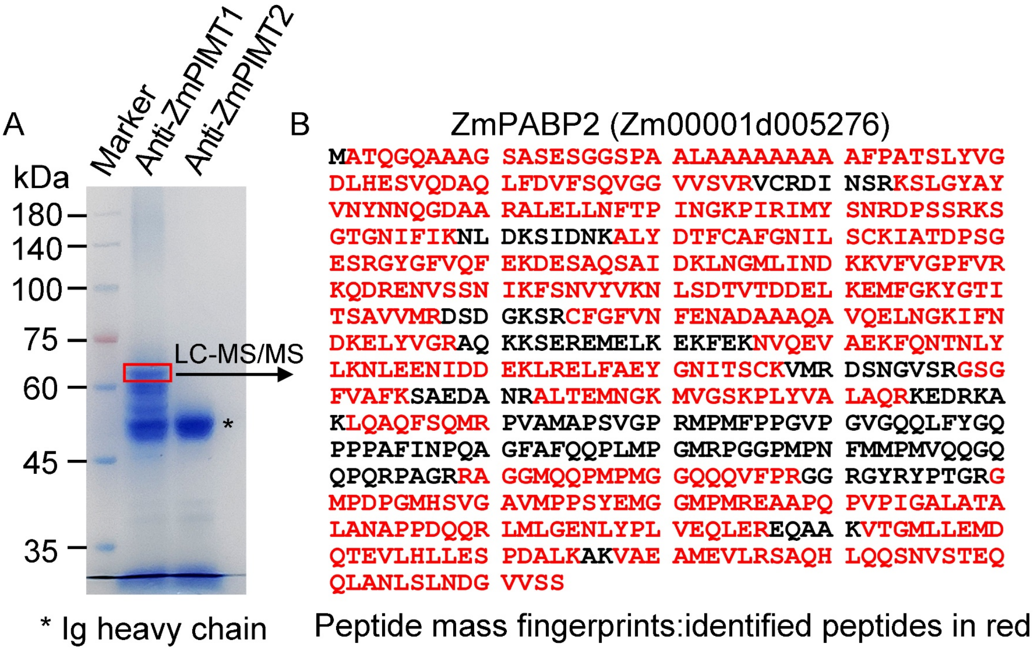

Supplementary Figure S12. ZmPABP2 was identified as one of the target proteins by LC-MS/MS. (Supports Figure 5).

(**A**) ZmPABP2 was identified as a ZmPIMT1 interacting protein by co-immunoprecipitation (Co-IP), trypsin digest, and mass spectrometry LC-MS/MS analysis. Coomassie-blue staining of the SDS-PAGE showed multiple proteins binding to ZmPIMT1; and the immunoglobulin heavy chain from the respective antibodies (denoted with an asterisk). Anti-ZmPIMT2 antibody was used as Co-IP control of a similar embryo protein extract.

(**B**) The protein indicated by a red box (as shown in A) was identified as ZmPABP2 protein by mass spectrometry. The amino acid coverage of the identified ZmPABP2 sequence is shown in red.

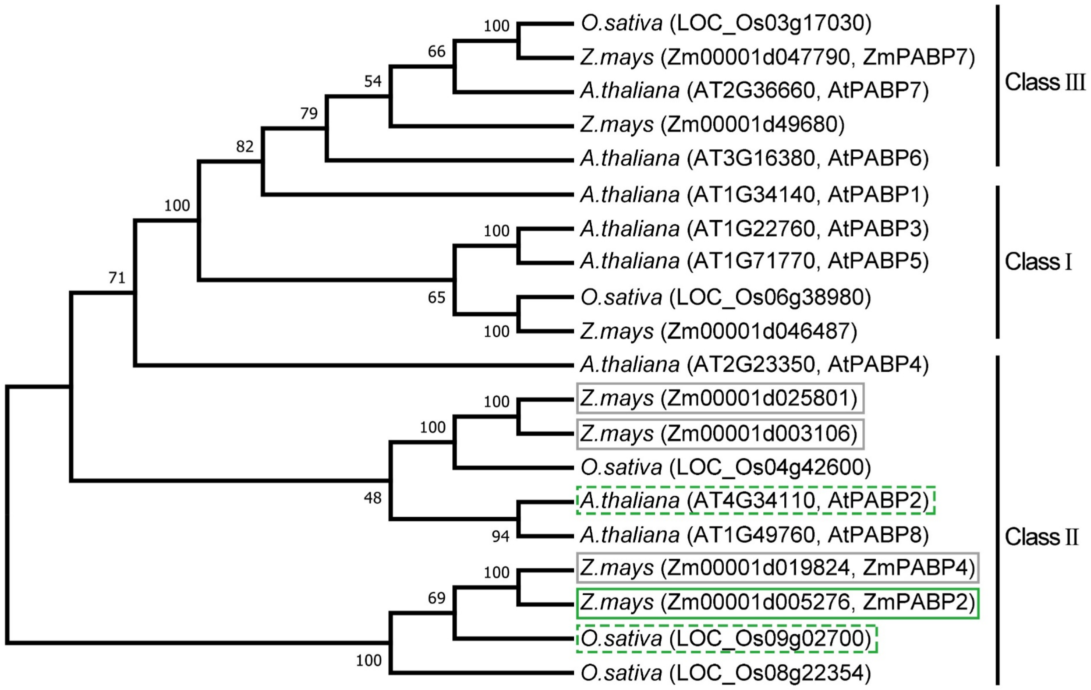

Supplementary Figure S13. A phylogenetic tree of relevant PABPs. (Supports Figure 5).

Sequences of PABPs in *Zea mays* (solid, green box outlining the ortholog studied here; solid gray box, the three other PIMT1 targets identified by Co-IP), *Arabidopsis thaliana* (dashed, green box outlining the ortholog studied here) and *Oryza sativa* (dashed, green box outlining the closest rice ortholog) were used. The gene ID and gene name (if named) were given in the tree. The evolutionary history was inferred using the Neighbor-Joining method. The percentage of replicate trees in which the associated taxa clustered together in the bootstrap test (1000 replicates) are shown next to the branches. The evolutionary distances were computed using the JTT matrix-based method and are in the units of the number of amino acid substitutions per site. Evolutionary analyses were conducted in MEGA7.

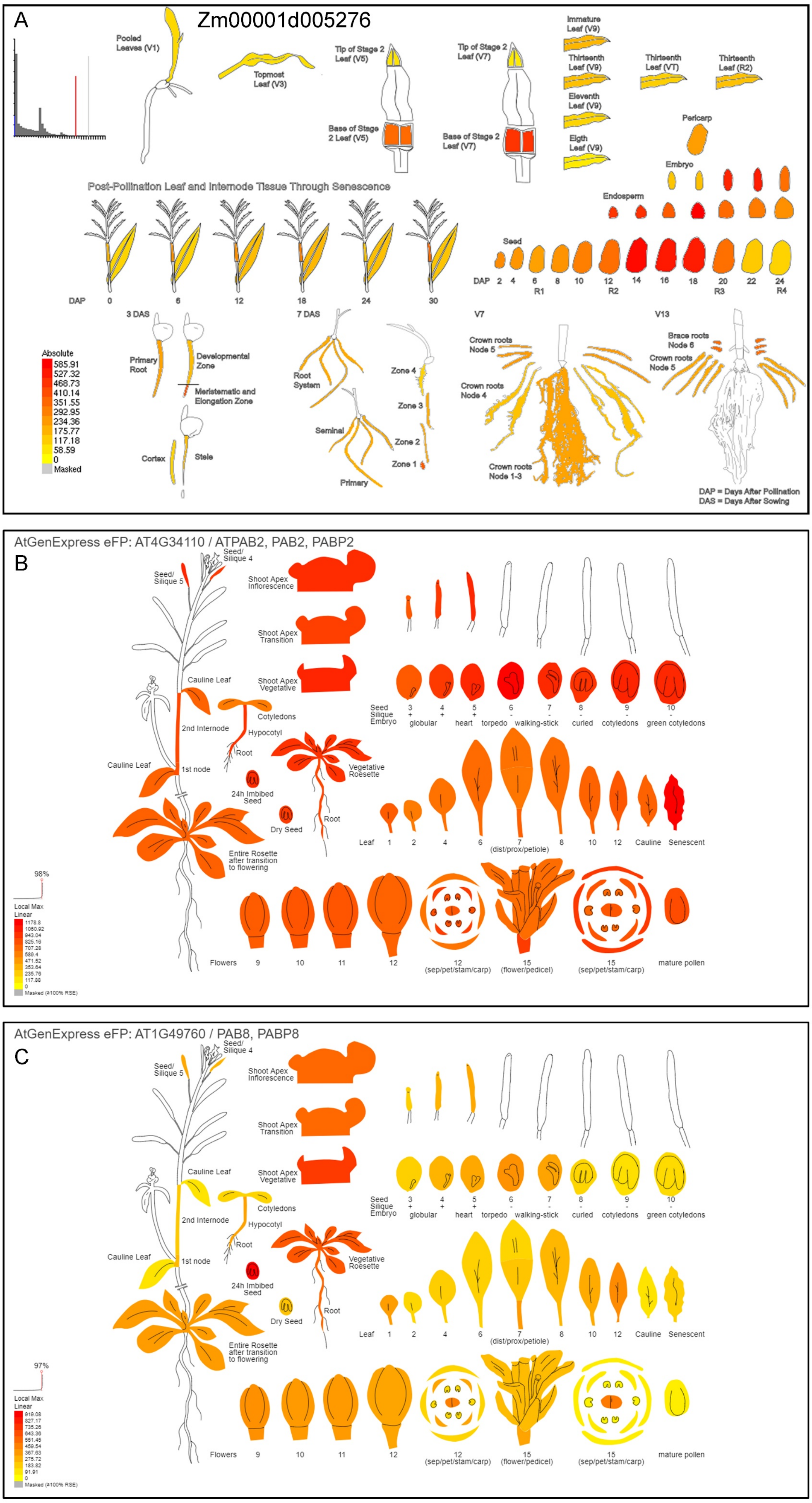
A
Zm00001d005276
Pooled Leaves (V1)
Topmost Leaf (V3)
Tip of Stage 2 Leaf (V5)
Base of Stage 2 Leaf (V5)
Tip of Stage 2 Leaf (V7)
Base of Stage 2 Leaf (V7)
Immature Leaf (V9)
Thirteenth Leaf (V9)
Thirteenth Leaf (VT)
Thirteenth Leaf (R2)
Eleventh Leaf (V9)
Eigth Leaf (V9)
Pericarp
Embryo
Endosperm
Seed
Post-Pollination Leaf and Internode Tissue Through Senescence
DAP 0 6 12 18 24 30
DAP 2 4 6 8 10 12 14 16 18 20 22 24
R1 R2 R3 R4
3 DAS
Primary Root
Developmental Zone
Meristematic and Elongation Zone
Cortex
Stele
7 DAS
Root System
Seminal
Primary
Zone 4
Zone 3
Zone 2
Zone 1
V7
Crown roots Node 5
Crown roots Node 4
Crown roots Node 1-3
V13
Brace roots Node 6
Crown roots Node 5
Absolute
585.91
527.32
468.73
410.14
351.55
292.95
234.36
175.77
117.18
58.59
0
Masked
DAP = Days After Pollination
DAS = Days After Sowing
AtGenExpress eFP: AT4G34110 / ATPAB2, PAB2, PABP2
B
Seed/ Silique 4
Seed/ Silique 5
Shoot Apex Inflorescence
Shoot Apex Transition
Shoot Apex Vegetative
Cauline Leaf
Cotyledons
2nd Internode
Hypocotyl
Root
Cauline Leaf
1st node
Vegetative Roesette
24h Imbibed Seed
Dry Seed
Root
Entire Rosette after transition to flowering
Seed Silique Embryo
3 + globular
4 + heart
5 + torpedo
6 - walking-stick
7 - curled
8 - cotyledons
9 - green cotyledons
10 -
Leaf 1 2 4 6 7 8 10 12 Cauline Senescent
(dist/prox/petiole)
98%
Local Max
Linear
1178.8
1060.92
943.04
825.16
707.28
589.4
471.52
353.64
235.76
117.88
0
Masked (≥100% RSE)
Flowers 9 10 11 12
12 (sep/pet/stam/carp)
15 (flower/pedicel)
15 (sep/pet/stam/carp)
mature pollen
AtGenExpress eFP: AT1G49760 / PAB8, PABP8
C
Seed/ Silique 4
Seed/ Silique 5
Shoot Apex Inflorescence
Shoot Apex Transition
Shoot Apex Vegetative
Cauline Leaf
Cotyledons
2nd Internode
Hypocotyl
Root
Cauline Leaf
1st node
Vegetative Roesette
24h Imbibed Seed
Dry Seed
Root
Entire Rosette after transition to flowering
Seed Silique Embryo
3 + globular
4 + heart
5 + torpedo
6 - walking-stick
7 - curled
8 - cotyledons
9 - green cotyledons
10 -
Leaf 1 2 4 6 7 8 10 12 Cauline Senescent
(dist/prox/petiole)
97%
Local Max
Linear
919.08
827.17
735.26
643.36
551.45
459.54
367.63
275.72
183.82
91.91
0
Masked (≥100% RSE)
Flowers 9 10 11 12
12 (sep/pet/stam/carp)
15 (flower/pedicel)
15 (sep/pet/stam/carp)
mature pollen

Supplementary Figure S14. The tissue expression profiles of relevant PABPs in maize and *Arabidopsis*, as reported in ePlants (respectively, https://bar.utoronto.ca/efp_maize and https://bar.utoronto.ca/efp/). (Supports Figure 5).

(**A**) ZmPABP2 tissue expression profiling in *Zea mays*; (**B**) AtPABP2 tissue expression profiling in *Arabidopsis thaliana*; (**C**) AtPABP8 tissue expression profiling in *Arabidopsis thaliana*.

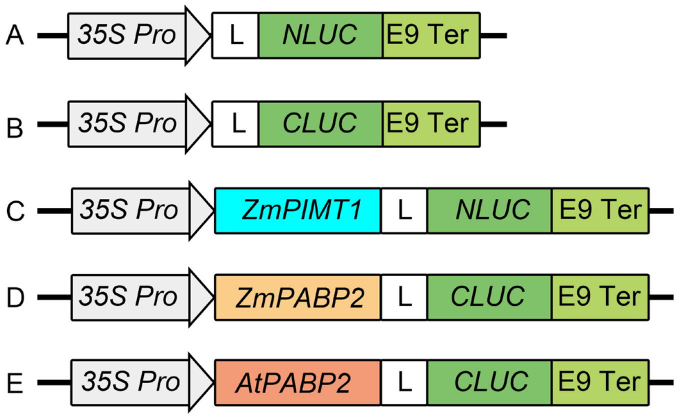

Supplementary Figure S15. Schematic representation of the expression vectors that were used in the luciferase complementation imaging (LCI) assay. (Supports Figure 5).

(**A**) The NLUC control expression vector permits a linker (MEDAKNIKKGPAPFYPLEDGTAGEQLHKA) and the N-terminal portion (1-416) of luciferase to accumulate in any leaf tissue in which the complete binary vector is introduced as DNA. (**B**) The CLUC control expression vector permits a linker (MEDAKNIKKGPAPFYPLEDGTAGEQLHKA) and the C-terminal portion of luciferase (398-550) to accumulate in any leaf tissue in which the complete binary vector is introduced as DNA. (**C**) The *ZmPIMT1:NLUC* expression vector permits the fusion of the ZmPIMT1 to a linker and NLUC (as described in (A)). (**D**) The *ZmPABP2:CLUC* permits the fusion of ZmPABP2 to a linker and CLUC (as described in (B)). (**E**) The *AtPABP2:CLUC* expression vector permits the fusion of AtPABP2 with CLUC(m) (as described in (B)). The abbreviations are as follows: 35S Pro, 35S cauliflower virus promoter; L, linker sequence; and E9 Ter, terminator sequence from PCAMBIA1300-CLUC vector.

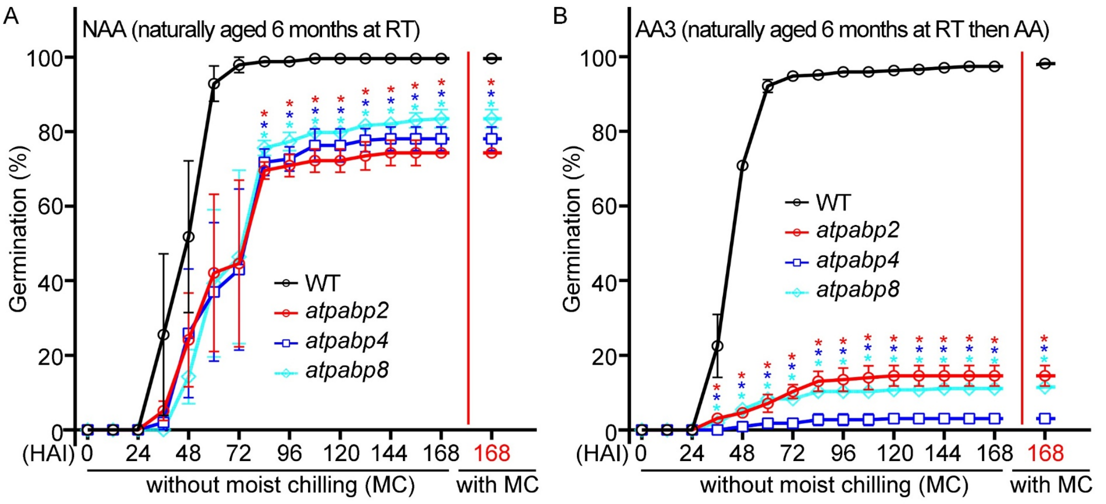

Supplementary Figure S16. The seed vigor of *atpabp2*, *atpabp4 and atpabp8* single mutant seeds was decreased after natural aging. (Supports Figure 6).

(**A**) Seed germination percentages of WT, *atpabp2*, *atpabp4 and atpabp8* single mutant *Arabidopsis thaliana* that were naturally aged six months at room temperature (RT) with no accelerated aging treatment (NAA). The completion of germination was calculated for every 12 h for a week and these numbers averaged and the standard error calculated and graphed. The values on the right side of the red line represent the germination percentages of the same seeds after 168 hours, followed by an additional 3 days of moist chilling and a week of incubation at room temperature. A two tailed ANOVA (alpha = 0.05) was used to assess whether there were statistically significant differences among means and Dunnett's test was subsequently used with WT Col as the control and each of the *atpabp* mutants compared to it. There were three replicates for each treatment and 80 seeds for each replicate. Those averages that differ significantly from the WT are denoted using a colored asterisk.

(**B**) A portion of the seeds from the same seed lots as in (A) were treated over saturated KCl (75 % RH) at 42 °C for 3 d (AA3) and then desiccated at room temperature for 24 h. The seed germination was then calculated as in **A**.

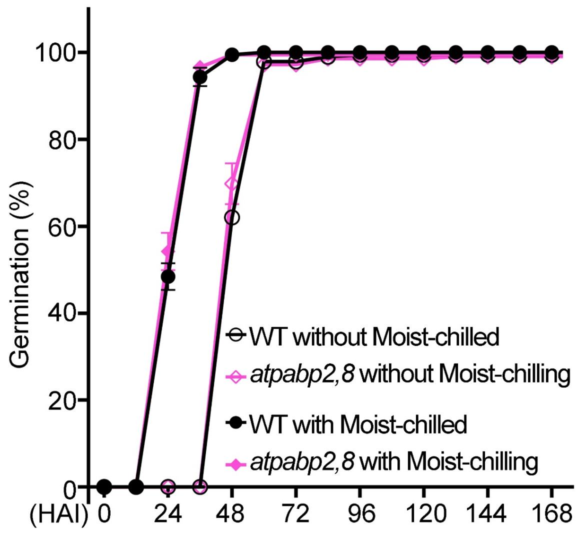

Supplementary Figure S17. The completion of germination of WT and *atpapb2,8* was statistically indistinguishable with or without moist chilling after 42 d dry after ripening. (Supports Figure 6). The completion of germination of WT and *atpabp2,8* seeds, dry after ripened for 42 days at RT to alleviate dormancy, with or without 3 d of moist chilling were scanned every 12 h for a week, there were three replicates for each treatment and 80 seeds for each replicate. The average and the standard error were calculated and graphed.

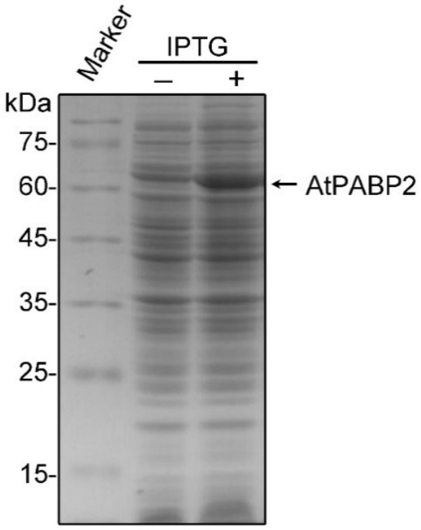

Supplementary Figure S18. Prokaryotic expression of AtPABP2 in bacteria. (Supports Figure 7). The total protein of bacteria *E. coli* (Rosetta gami2, DE3) cells that were un-induced (-) or induced (+) with IPTG, respectively. The arrow indicates the expressed AtPABP2 protein.

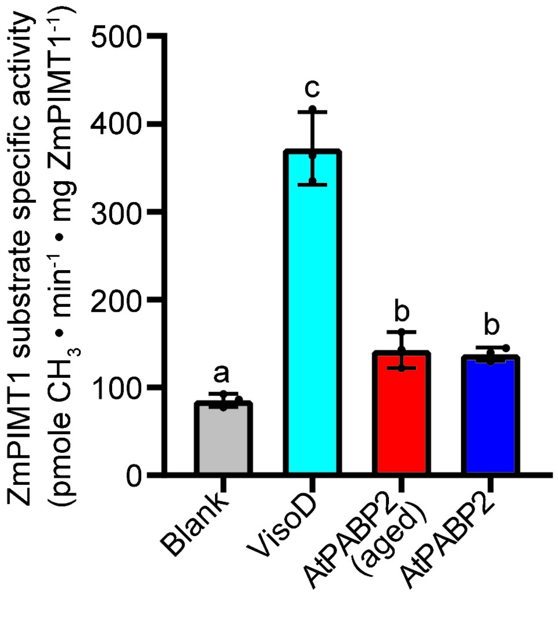

Supplementary Figure S19: **Comparison of specific activity of ZmPIMT1 substrates. (Supports Figure 7).** ZmPIMT1 is capable of methylating the isoAsp-

containing peptide VYP-(isoD)-HA (denoted as VisoD), as well as isoAsp-containing AtPABP2, either aged (3 h at 37 °C) or unaged, relative to the substrate blank. Specific activity (SA), generated from the methylation of isoAsp and its subsequent release as tritiated methanol upon the formation of succinimide, was monitored following a 2 h vapor diffusion assay. Different lowercase letters denote statistically significant differences (alpha = 0.05) in SA between the isoAsp substrates and the blank control, as determined by one-tailed Dunnett's test following ANOVA. Data are presented as means ± SD (n = 3).

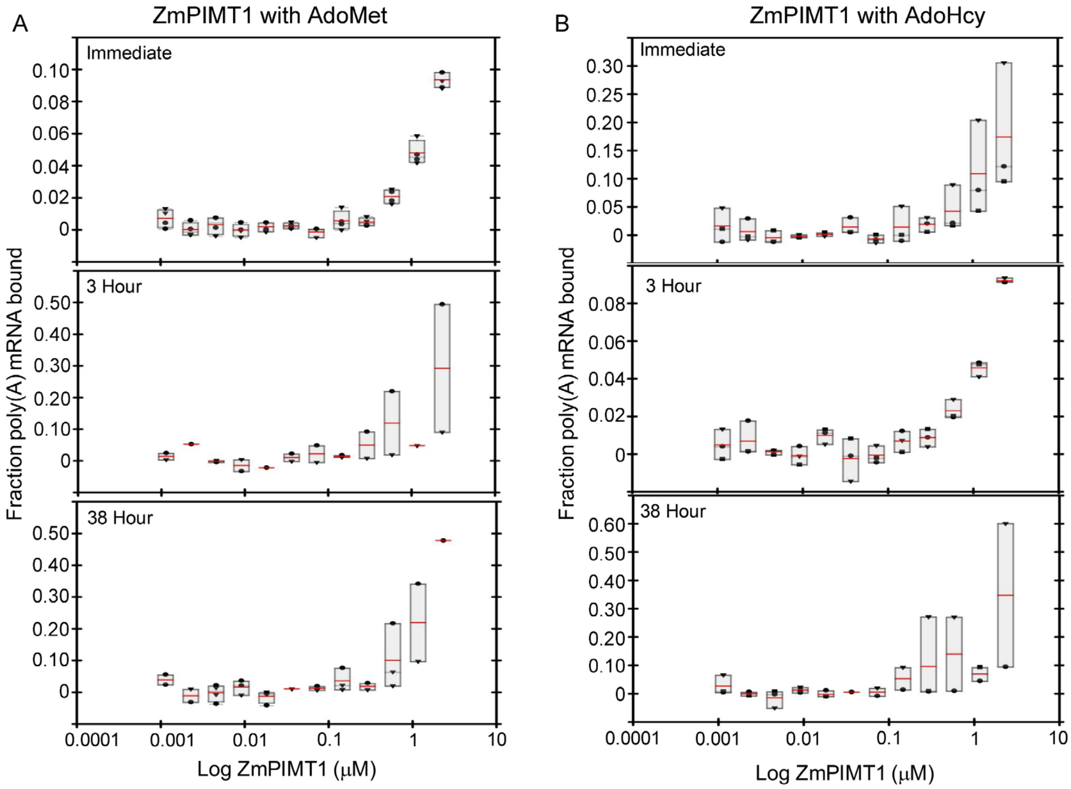

Supplementary Figure S20: **ZmPIMT1 does not bind poly(A) mRNA. (Supports Figure 7).**

There were no significant dissociation constants (*K_D*) generated at any point during the incubation of ZmPIMT1 and fluorescently labeled, poly(A) mRNA in the presence of **(A)** AdoMet or **(B)** AdoHcy. Time points presented were: immediately upon assay set up; 3 h and; 38 h after the commencement of the assay, at which point the assay was terminated. Upon analysis, all assays were flagged by Dianthus screening analysis software as providing insufficient saturation to provide a *K_D*. The highest concentration of ZmPIMT1 in these binding assays (2.2 μM) was the amount used to repair AtPABP2 in the AtPABP:poly(A) mRNA binding assays. In the box plot, the box's lower and upper edges represent the minimum and maximum values of the dataset, respectively, with an internal red line marking the mean (n=3).

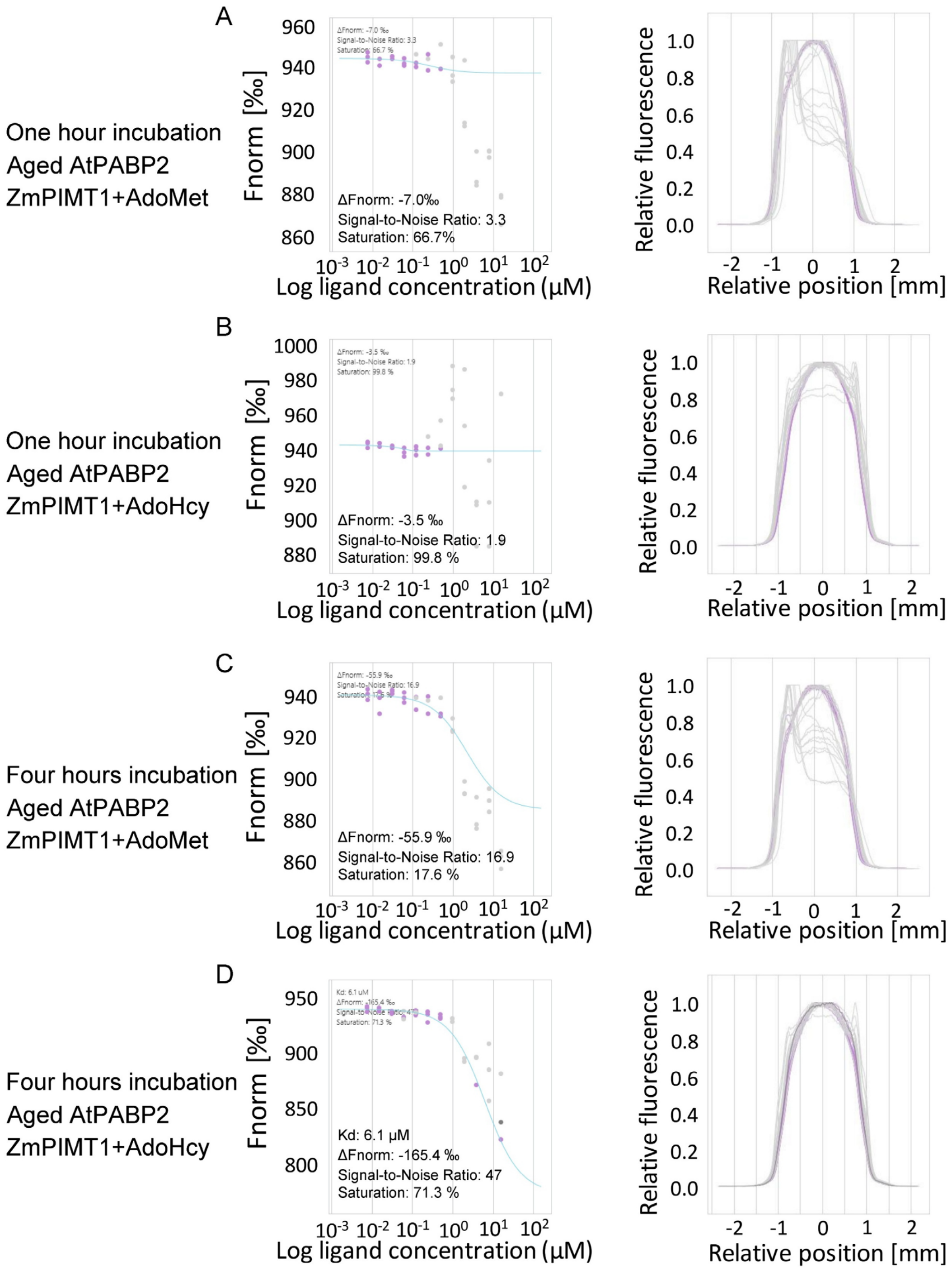

Supplementary Figure S21: The raw well scans and TRIC traces of aged AtPABP2 after 1 and 4 h incubation. (Supports Figure 7).

There were no legitimate dissociation constants (K_D) generated during incubation for 1 h of aged AtPABP2 in the presence of poly(A) mRNA and ZmPIMT1 (**A**) with AdoMet or (**B**) with AdoHcy. The same is true after 4 h of incubation (**C**) with AdoMet or (**D**) with AdoHcy. Upon analysis, all assays were flagged by Dianthus screening analysis software (DI.SA) as suffering from one or more of the following issues: insufficient saturation, insufficient ΔFnorm, a signal-to-noise ratio that was too low, and/or aggregation, that prevented estimation of a valid K_D. The highest concentration of

ZmPIMT1 in these binding assays (2.2 μM) was the amount used to repair AtPABP2 in the AtPABP2: poly(A) mRNA binding assays. The data points and fluorescence curves in grey denote unusable data for one, or several, of the reasons mentioned above. Dark grey data points were flagged as outliers by DI.SA. Data points and fluorescence curves in purple are acceptable. Relative fluorescence curves (measured across the width of the well) where the fluorescence maximum should be centered in the well (at 0 mm).

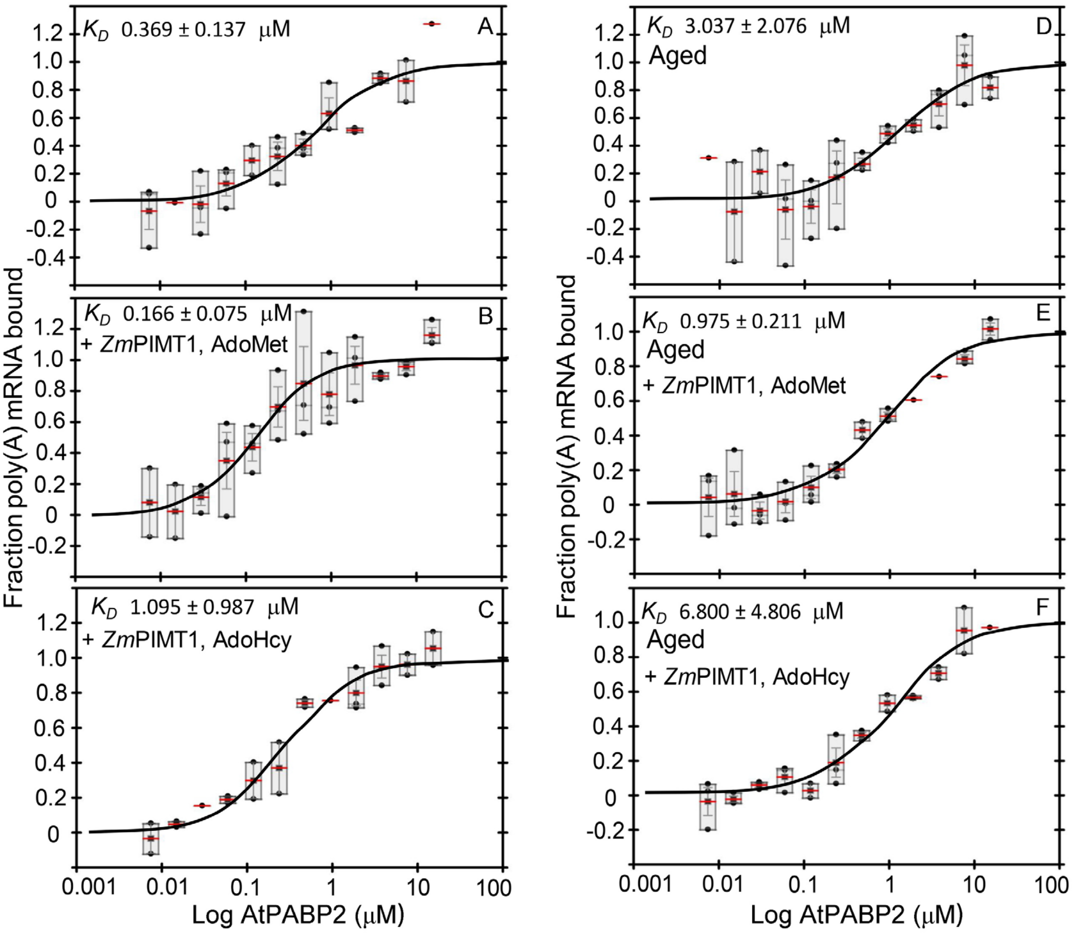

Supplementary Figure S22: Detection of poly(A) mRNA binding affinity of AtPABP2. (Supports Figure 7).

AtPABP2 binds poly(A) mRNA (**A**) without or (**B**) with constant maintenance by ZmPIMT1 in the presence of AdoMet. (**C**) Even when the activity of ZmPIMT1 is inhibited by the presence of AdoHcy, the K_D of AtPABP2 for poly(A) mRNA is uninfluenced. Aged AtPABP2 tends to have K_D for poly(A) mRNA that are greater than that for unaged AtPABP2. (**D**) Aged AtPABP2 incubated with poly(A) mRNA for 22 h. (**E**) Aged AtPABP2 incubated with poly(A) mRNA for 22 h in the presence of ZmPIMT1 and AdoMet. (**F**) Aged AtPABP2 incubated with poly(A) mRNA for 22 h in the presence of ZmPIMT1 and AdoHcy. The estimate is the average K_D from the three replications and the standard error of the mean. The K_D of unaged AtPABP2 for poly(A) mRNA, in the presence of ZmPIMT1 and AdoMet, is statistically significantly less (ANOVA, $\alpha = 0.05$) than the K_D of aged AtPABP2 also in the presence of ZmPIMT1

and AdoMet. In the box plot, the box's lower and upper edges represent the minimum and maximum values of the dataset, respectively, with an internal red line marking the mean. The whiskers extending from the mean represent the stand error bars (n=3).

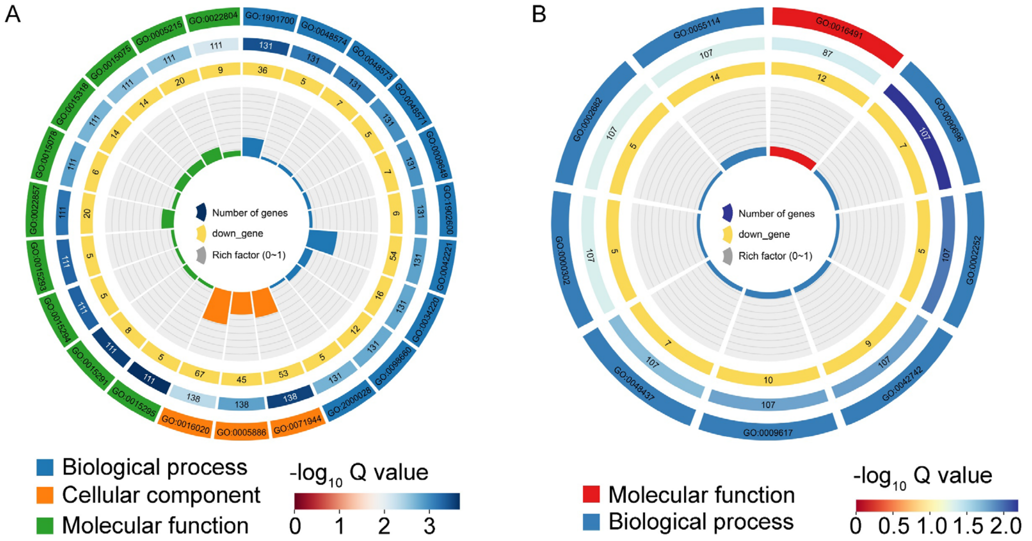

Supplementary Figure S23. Circular plot of gene ontology (GO) enrichment analysis for differentially expressed genes (DEGs). (Supports Figure 7).

(A) GO terms enriched for transcriptome DEGs. Outermost ring: Displays the significantly enriched GO terms. The numbers outside indicate the scale of DEG counts. Colors represent different ontologies. Second ring: Shows the total number of genes and associated Q-values. Third ring: Represents the number of down-regulated DEGs, with distinct colors indicating regulation direction. Innermost ring: Indicates the enrichment factor value for each GO term. Significant GO terms are categorized as follows, Biological Process: response to oxygen-containing compound (GO:1901700), long-day photoperiodism and flowering (GO:0048574, GO:0048571), photoperiodism (GO:0009648), proton transmembrane transport (GO:1902600), response to chemical (GO:0042221), monoatomic ion transmembrane transport (GO:0034220); Cellular Component: cell periphery (GO:0071944), plasma membrane (GO:0005886), membrane (GO:0016020). Molecular Function: proton symporter activity (GO:0015295), secondary active transmembrane transporter activity (GO:0015291), monoatomic cation symporter activity (GO:0015294), symporter activity (GO:0015293), transmembrane transporter activity (GO:0022857), proton transmembrane transporter activity (GO:0015078), inorganic molecular entity transmembrane transporter activity (GO:0015318), monoatomic ion transmembrane transporter activity (GO:0015075).

(B) GO terms enriched for translation efficiency DEGs. Outermost ring: Displays the significantly enriched GO terms. The numbers outside indicate the scale of DEG counts. Colors represent different ontologies. Second ring: Shows the total number of genes and associated Q-values. Third ring: Represents the number of down-regulated DEGs, with distinct colors indicating regulation direction. Innermost ring: Indicates the enrichment factor value for each GO term.

Significant GO sub-terms are displayed in the outermost ring and categorized as follows: Molecular Function: oxidoreductase activity (GO:0016491); Biological Process: post-embryonic plant organ development (GO:0090696); immune effector process (GO:0002252); defense response to bacterium (GO:0042742); response to bacterium (GO:0009617); floral organ development (GO:0048437); response to reactive oxygen species (GO:0000302); regulation of immune system process (GO:0002682); oxidation-reduction process (GO:0055114).

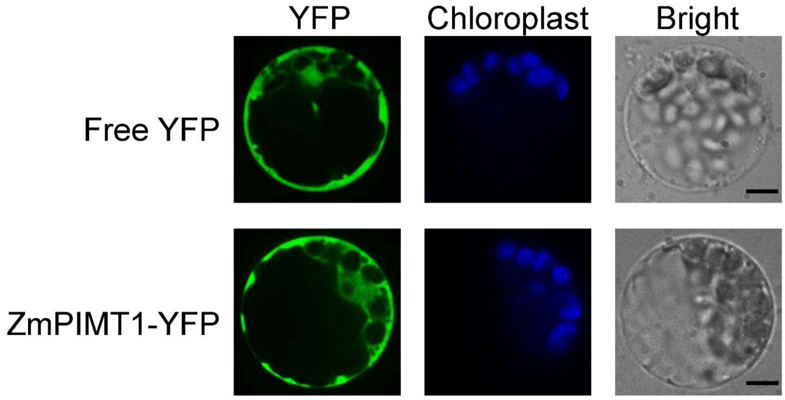

Supplementary Figure S24: The subcellular localization of ZmPIMT1. YFP and the ZmPIMT1-YFP fusion were visualized after transient expression in maize leaf protoplasts. YFP fluorescence and chloroplast autofluorescence were detected by confocal microscopy. The scale bar in the bright field image is 10 μm. **(Supports Figure 5).**

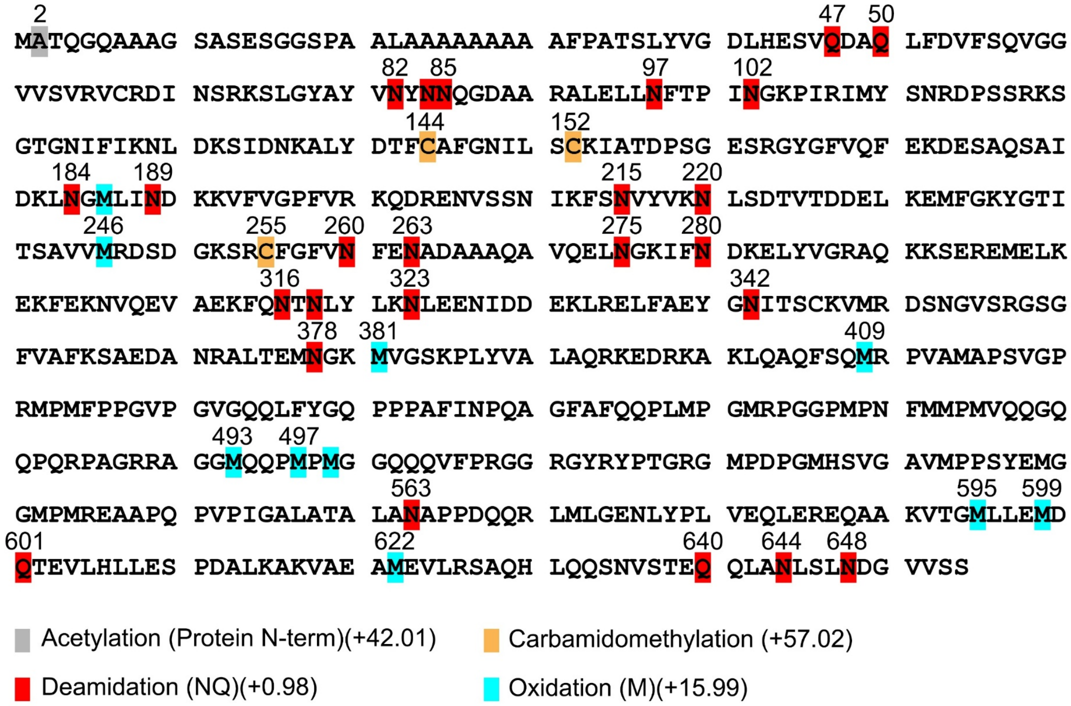

Supplementary Figure S25. Identification of ZmPABP2 post-translational modification sites by Co-IP and by LS-MS/MS in *zmpimt1* seed. (Supports Figure 7). The sites of acetylation (gray), carbamidomethylation (orange), deamidation (red), and oxidation (blue) are shown on the ZmPABP2 sequence.

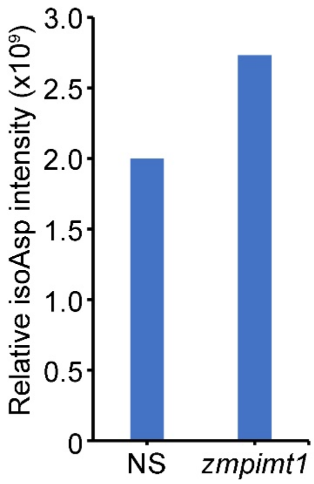

Supplementary Figure S26. Comparison of the accumulation of isoAsp in ZmPABP2 between *zmpimt1* and its NS control under heat stress. (Supports Figure 7). Relative isoAsp intensity of ZmPABP2 was compared between *zmpimt1* seeds and its null segregant (NS) control. Seeds were germinated at 25 °C for 16 h and then subjected to a 2 h heat shock at 42 °C prior to analysis. IsoAsp levels were quantified by liquid chromatography tandem mass spectrometry (LC-MS/MS) using samples of separated embryos (0.3 g).

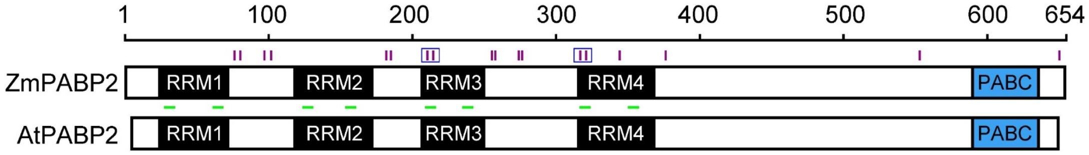

Supplementary Figure S27. Depiction of the alignment of the maize ZmPABP2 and *Arabidopsis* AtPABP2 proteins. (Supports Figure 7).

Magenta tick marks above the ZmPABP2 sequence indicate deamidated asparagine residues (see Supplementary Figure S25). RNA recognition motifs (RRMs) are shaded in black. Within each RRM, the conserved RNP1 and RNP2 motifs, which mediate RNA binding, are highlighted with green lines. RNP, ribonucleoprotein. The C-terminal PABC domain, a peptide-binding domain conserved in poly(A)-binding proteins, is shaded in blue.
