# Supplementary figures and images for: Natural variation in the ZmPIMT1 promoter enhances seed aging tolerance by regulating PABP2 repair in maize
Source: Plant Cell. 2025 Sep 18;37(10):koaf217. doi: 10.1093/plcell/koaf217 (PMC12510314; doi:10.1093/plcell/koaf217)

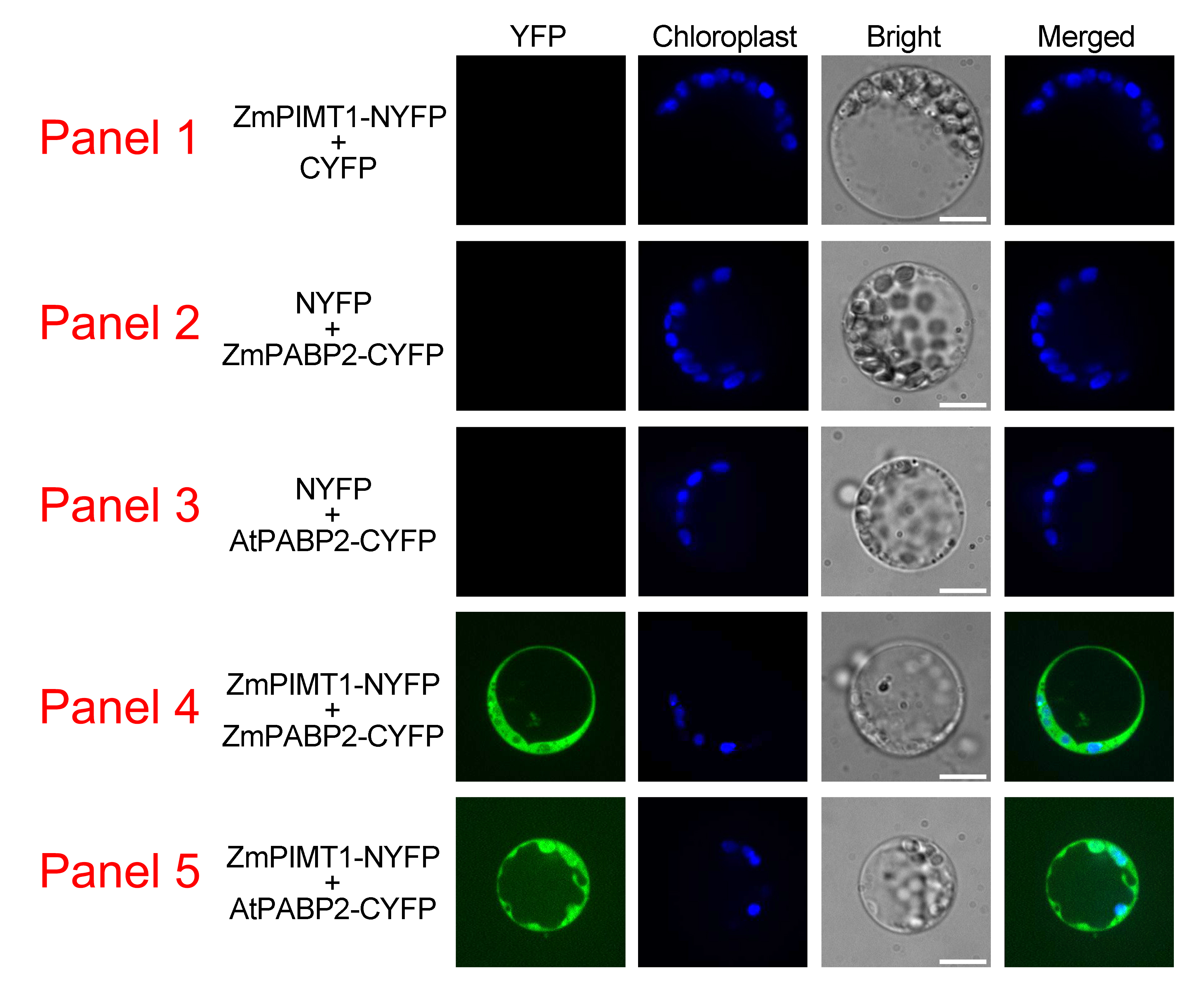

Supplement: koaf217_Supplementary_Data [file koaf217_supplementary_data.zip › Figure 5C source files/Figure 5C source files/Figure 5C-descripiton.jpg]

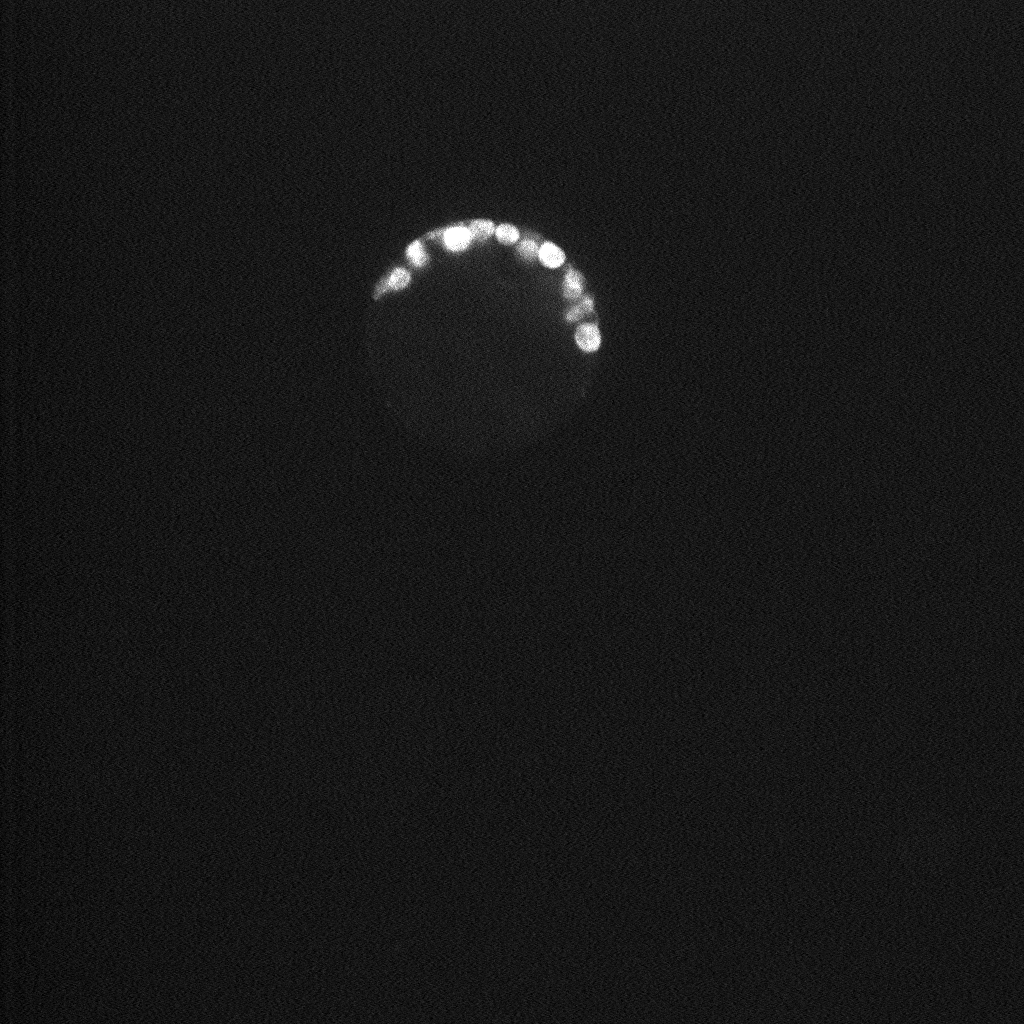

Supplement: koaf217_Supplementary_Data [file koaf217_supplementary_data.zip › Figure 5C source files/Figure 5C source files/Figure 5C-panel 1-ZmPIMT1-NYFP+CYFP source files/SPYN-ZmPIMT1+SPYC-10_w0000.tif]

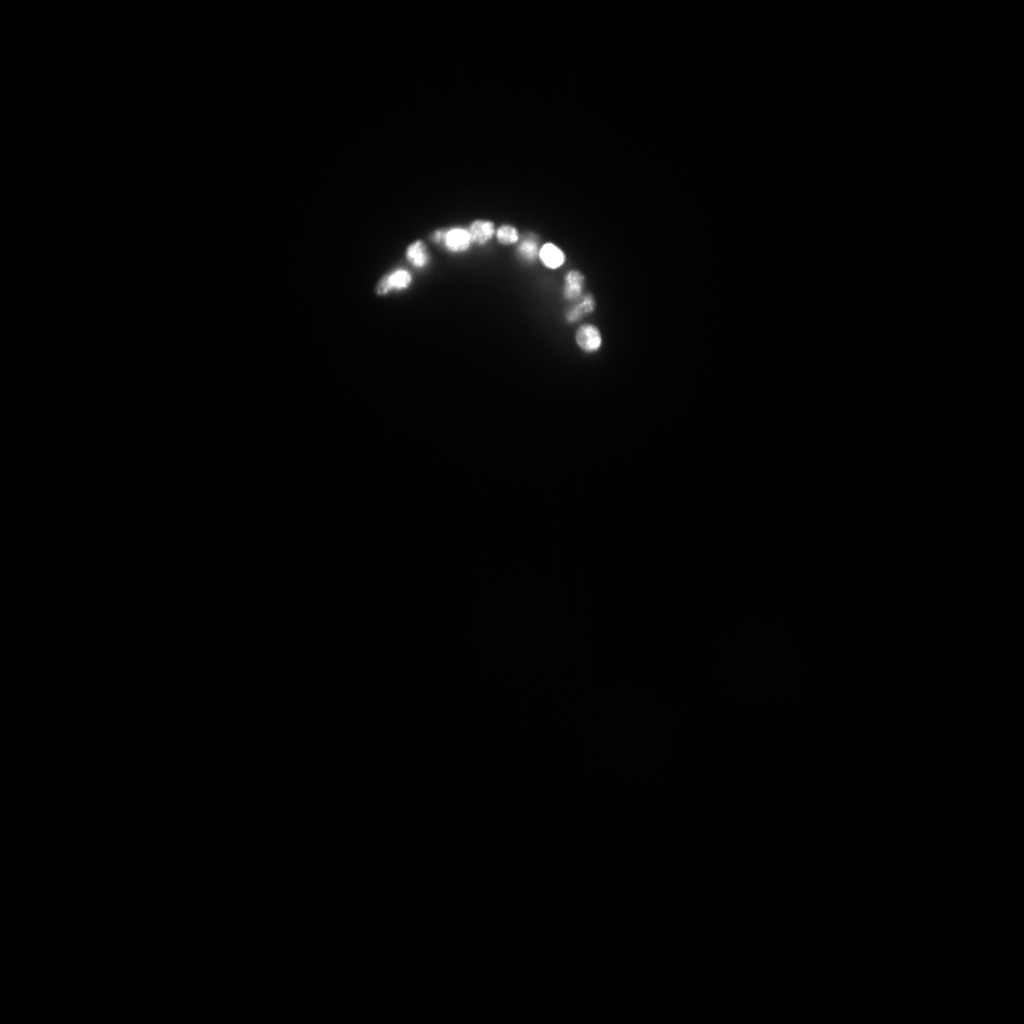

Supplement: koaf217_Supplementary_Data [file koaf217_supplementary_data.zip › Figure 5C source files/Figure 5C source files/Figure 5C-panel 1-ZmPIMT1-NYFP+CYFP source files/SPYN-ZmPIMT1+SPYC-10_w0001.tif]

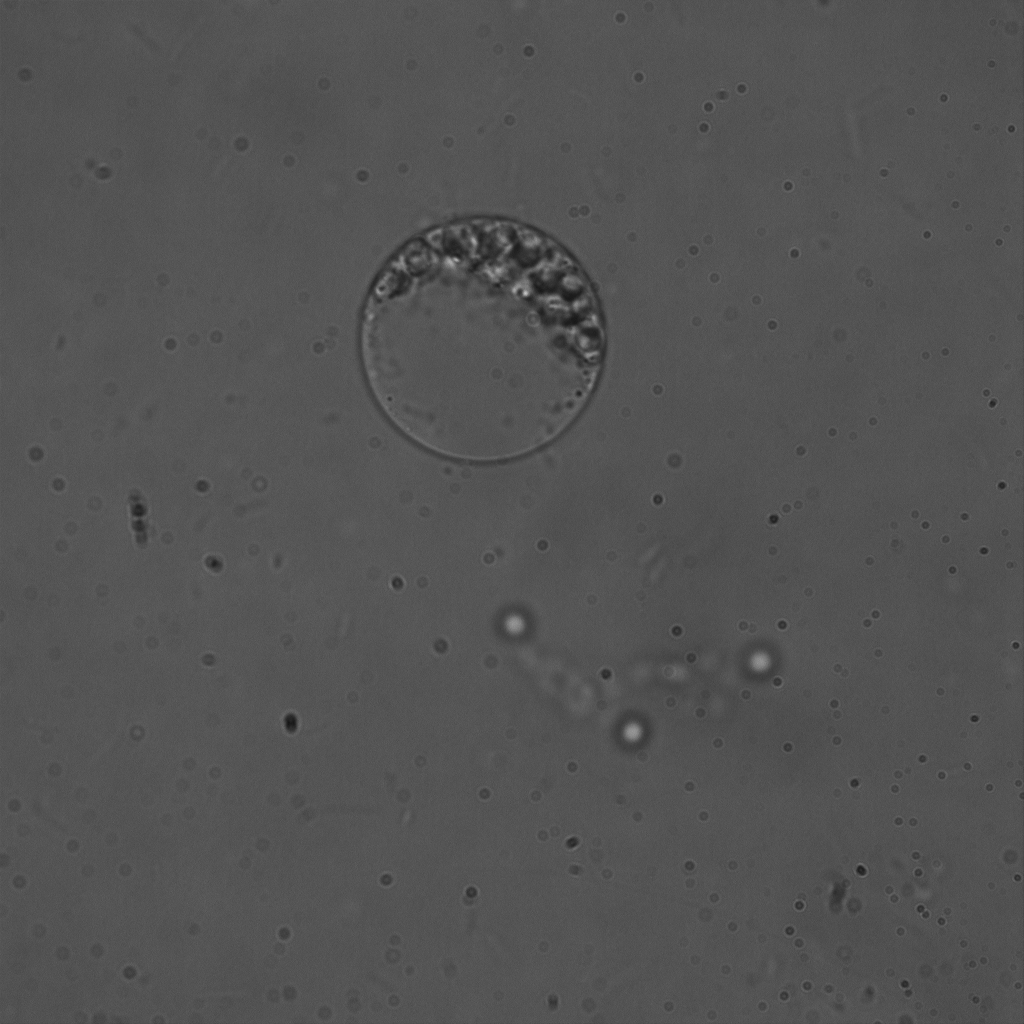

Supplement: koaf217_Supplementary_Data [file koaf217_supplementary_data.zip › Figure 5C source files/Figure 5C source files/Figure 5C-panel 1-ZmPIMT1-NYFP+CYFP source files/SPYN-ZmPIMT1+SPYC-10_w0002.tif]

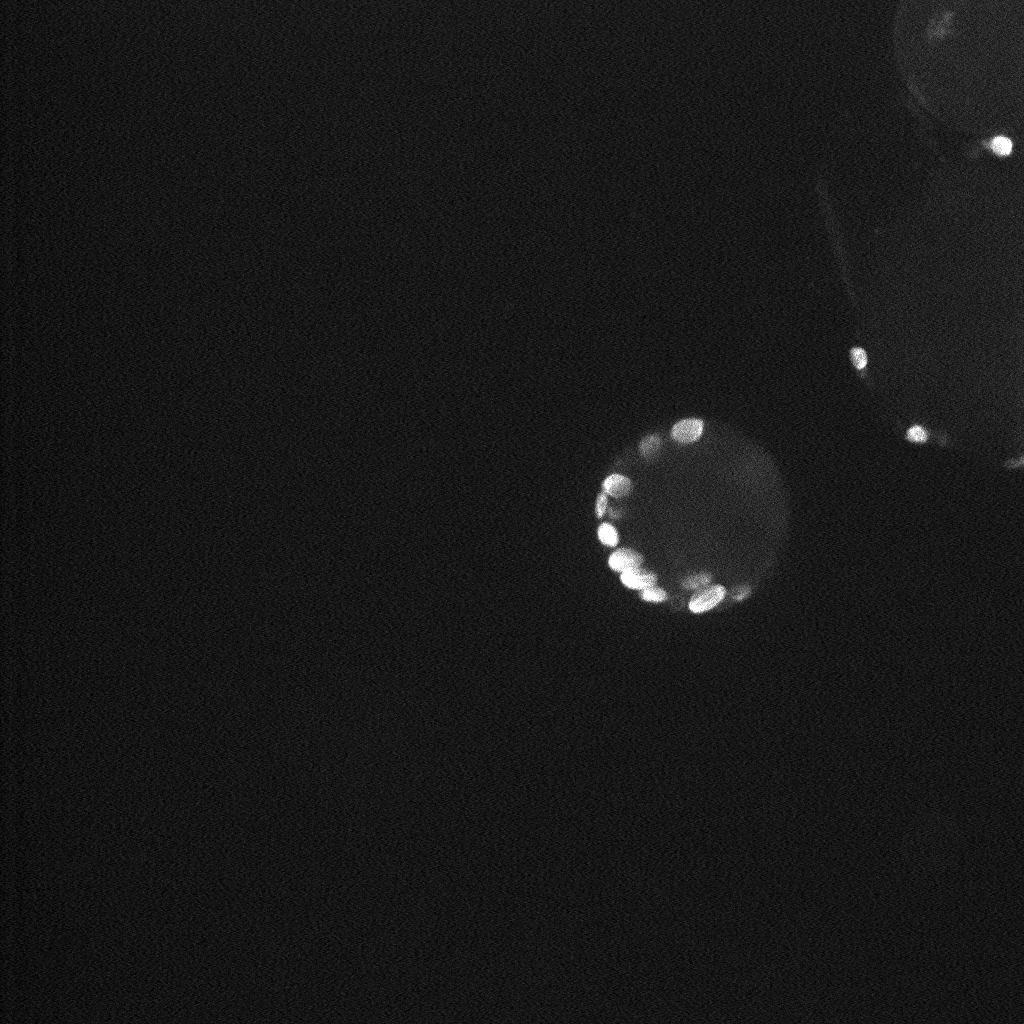

Supplement: koaf217_Supplementary_Data [file koaf217_supplementary_data.zip › Figure 5C source files/Figure 5C source files/Figure 5C-panel 2-NYFP+ZmPABP2-CYFP source files/SPYN+SPYC-ZmPAB2-6_w0000.tif]

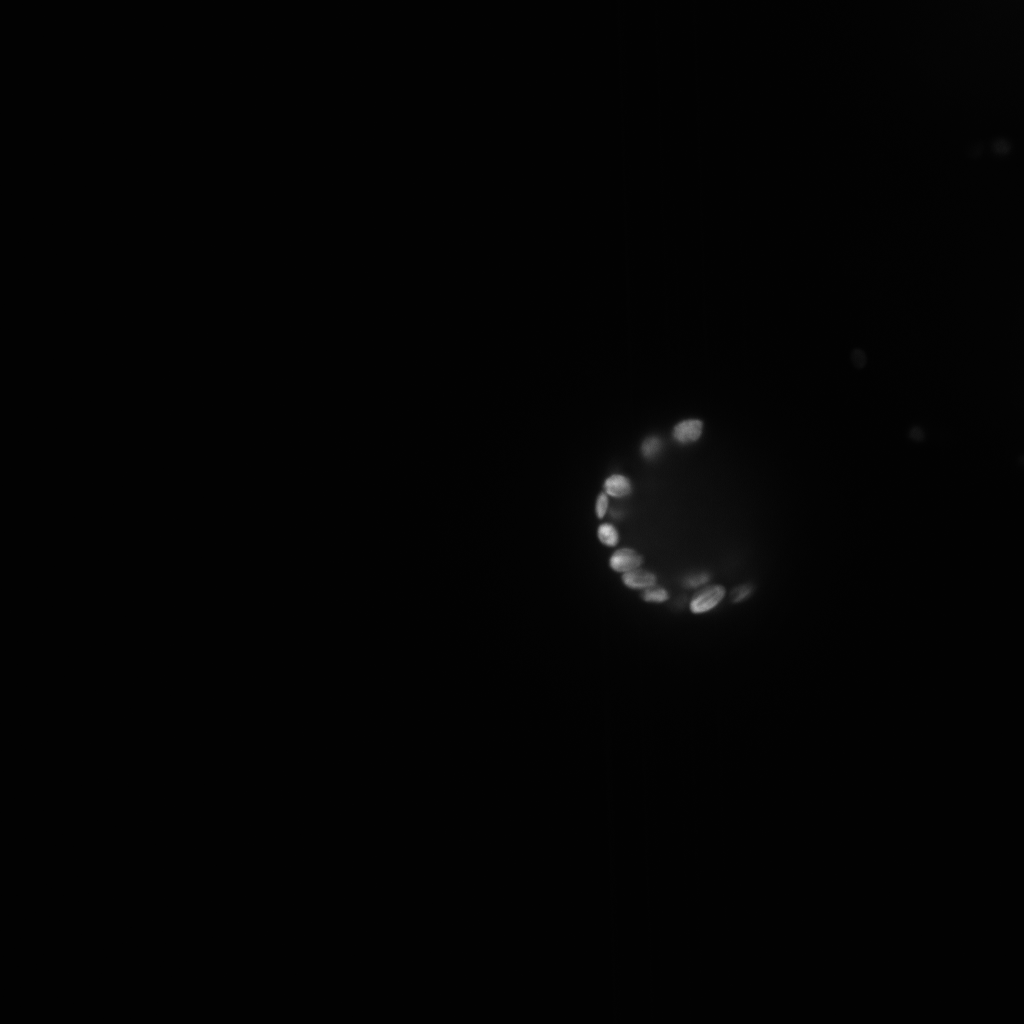

Supplement: koaf217_Supplementary_Data [file koaf217_supplementary_data.zip › Figure 5C source files/Figure 5C source files/Figure 5C-panel 2-NYFP+ZmPABP2-CYFP source files/SPYN+SPYC-ZmPAB2-6_w0001.tif]

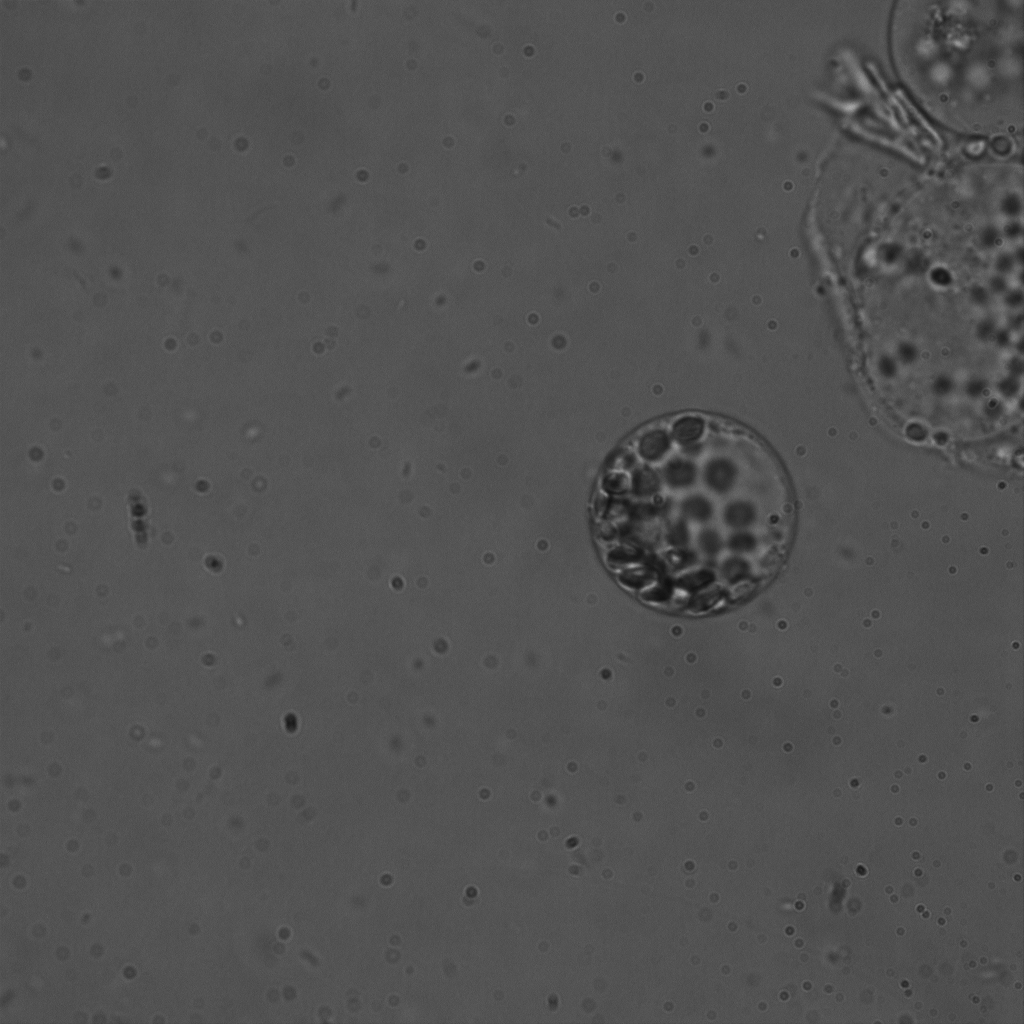

Supplement: koaf217_Supplementary_Data [file koaf217_supplementary_data.zip › Figure 5C source files/Figure 5C source files/Figure 5C-panel 2-NYFP+ZmPABP2-CYFP source files/SPYN+SPYC-ZmPAB2-6_w0002.tif]

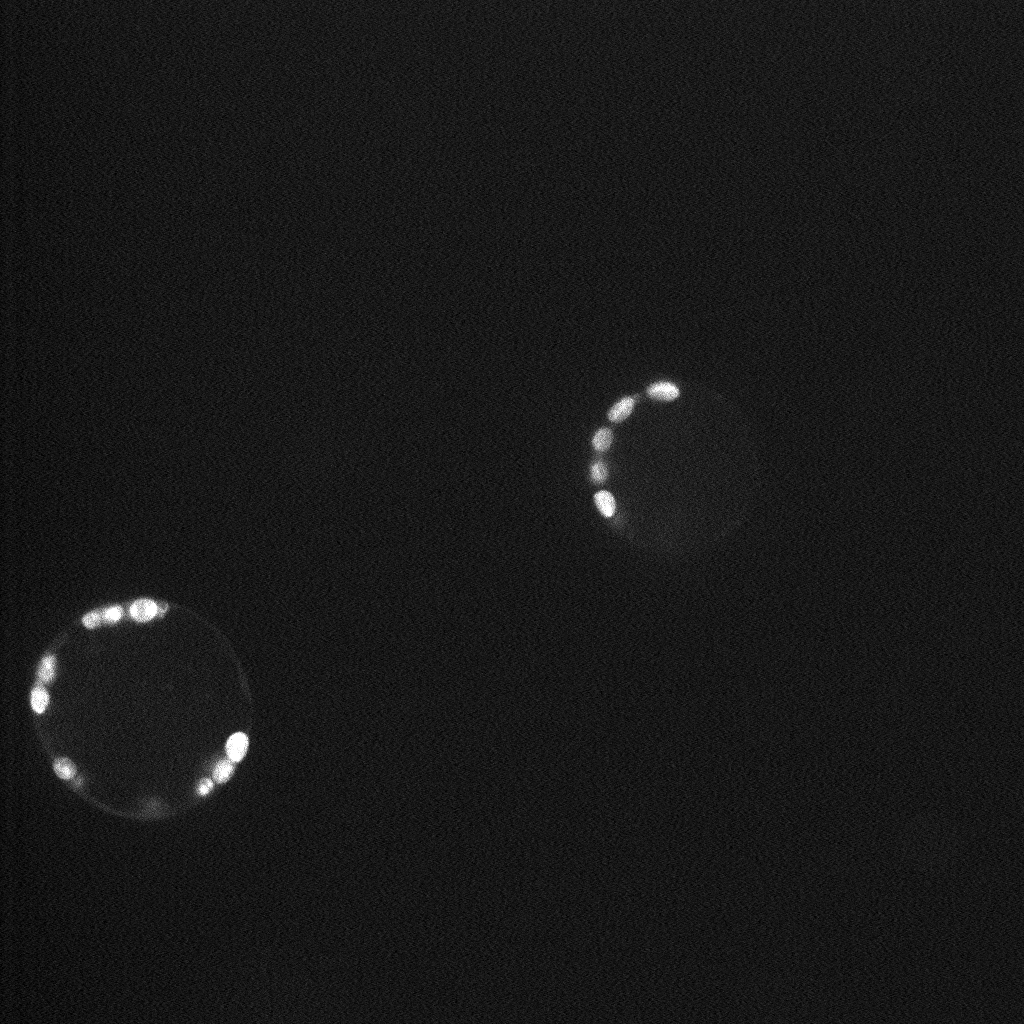

Supplement: koaf217_Supplementary_Data [file koaf217_supplementary_data.zip › Figure 5C source files/Figure 5C source files/Figure 5C-panel 3-NYFP+AtPABP2-CYFP source files/SPYN+SPYC-AtPAB2-9_w0000.tif]

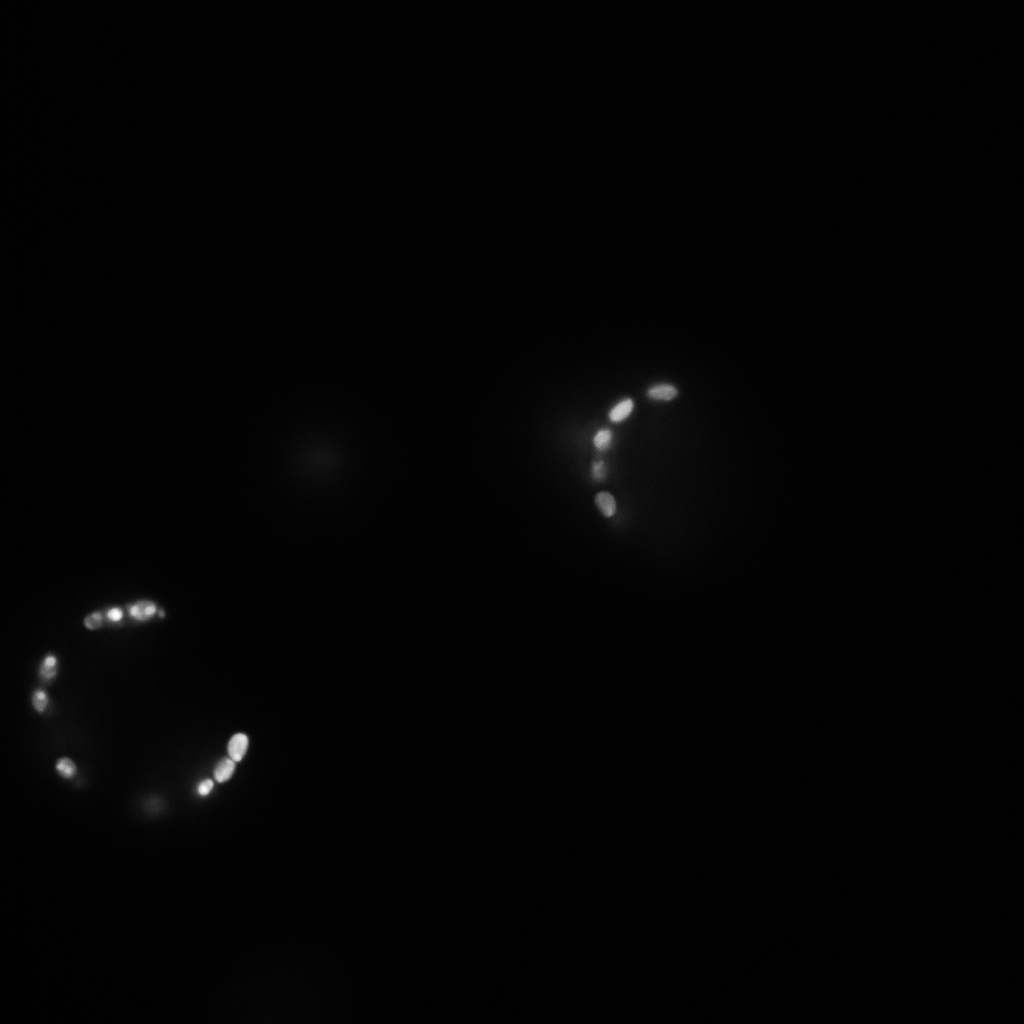

Supplement: koaf217_Supplementary_Data [file koaf217_supplementary_data.zip › Figure 5C source files/Figure 5C source files/Figure 5C-panel 3-NYFP+AtPABP2-CYFP source files/SPYN+SPYC-AtPAB2-9_w0001.tif]

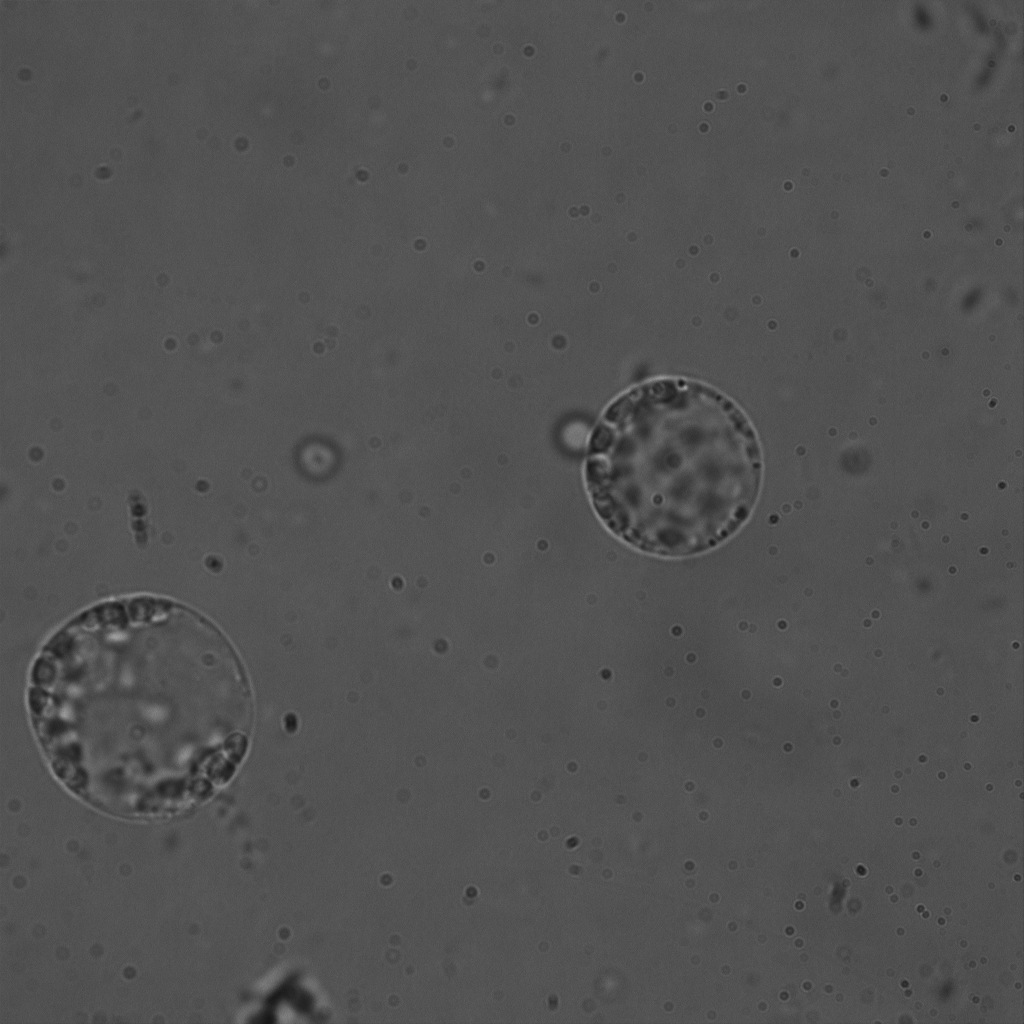

Supplement: koaf217_Supplementary_Data [file koaf217_supplementary_data.zip › Figure 5C source files/Figure 5C source files/Figure 5C-panel 3-NYFP+AtPABP2-CYFP source files/SPYN+SPYC-AtPAB2-9_w0002.tif]

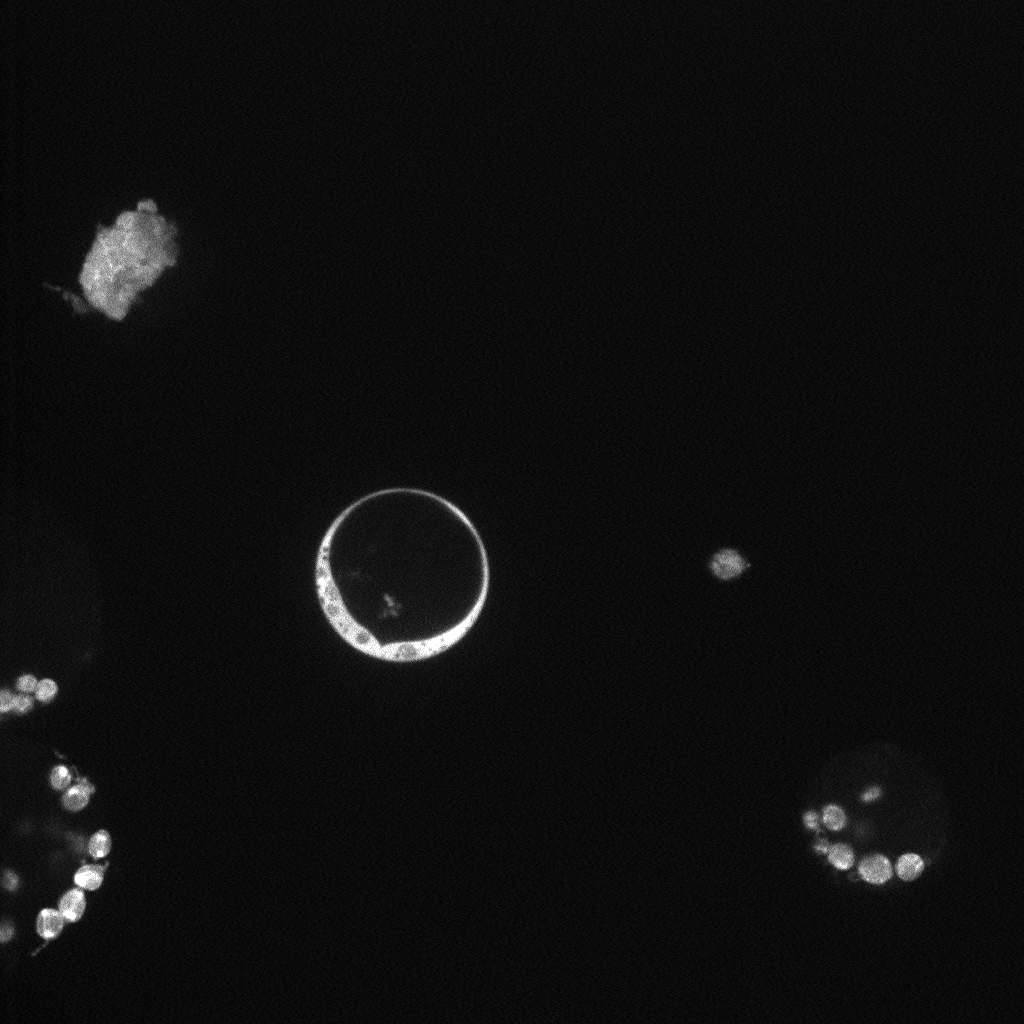

Supplement: koaf217_Supplementary_Data [file koaf217_supplementary_data.zip › Figure 5C source files/Figure 5C source files/Figure 5C-panel 4-ZmPIMT1-NYFP+ZmPABP2-CYFP source files/SPYN-ZmPIMT1+SPYC-ZmPAB2-10_w0000.tif]

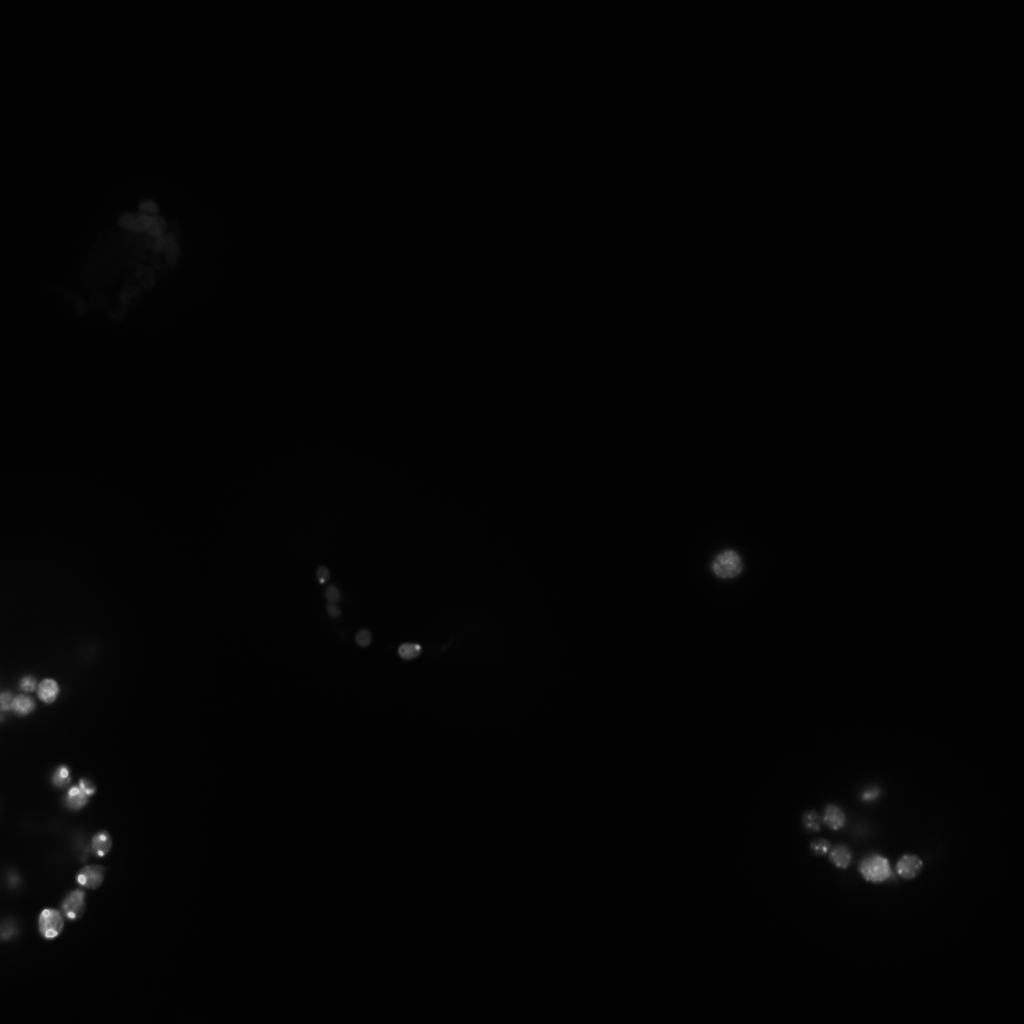

Supplement: koaf217_Supplementary_Data [file koaf217_supplementary_data.zip › Figure 5C source files/Figure 5C source files/Figure 5C-panel 4-ZmPIMT1-NYFP+ZmPABP2-CYFP source files/SPYN-ZmPIMT1+SPYC-ZmPAB2-10_w0001.tif]

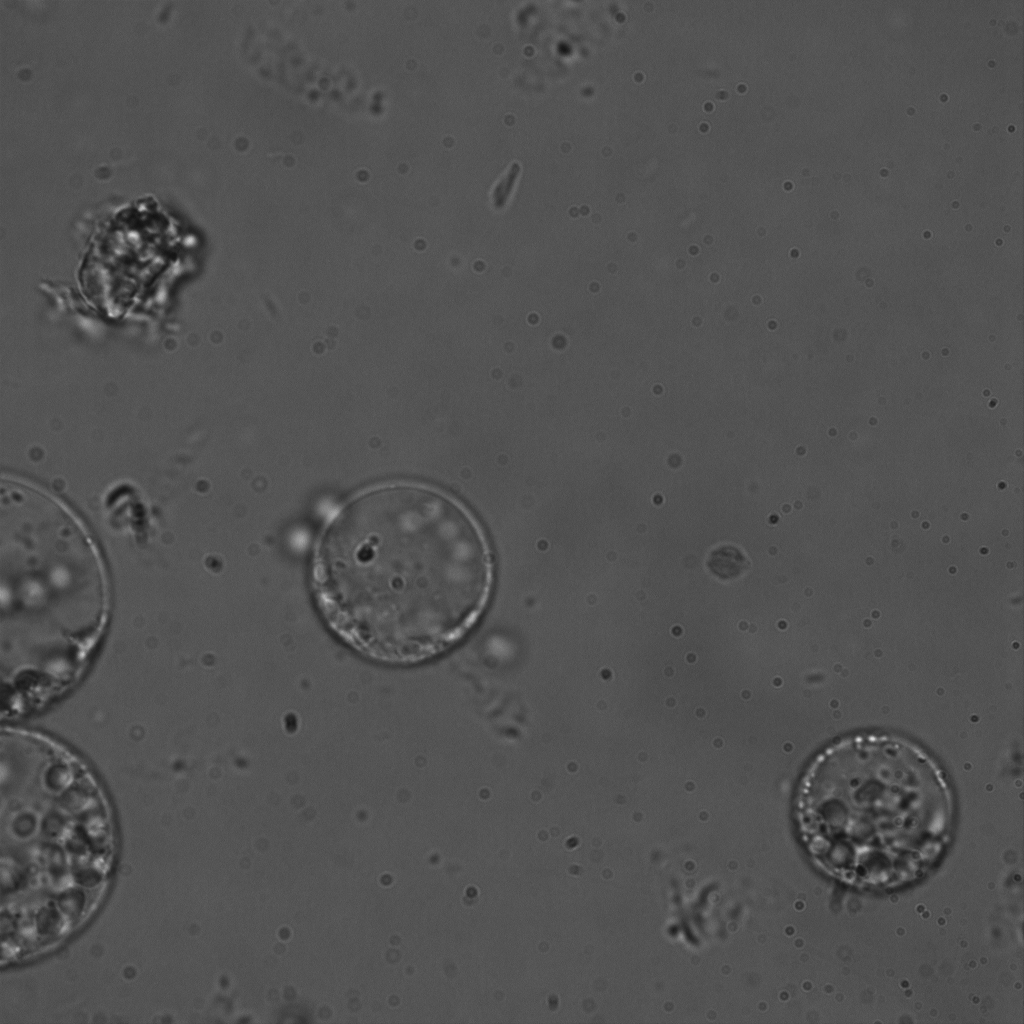

Supplement: koaf217_Supplementary_Data [file koaf217_supplementary_data.zip › Figure 5C source files/Figure 5C source files/Figure 5C-panel 4-ZmPIMT1-NYFP+ZmPABP2-CYFP source files/SPYN-ZmPIMT1+SPYC-ZmPAB2-10_w0002.tif]

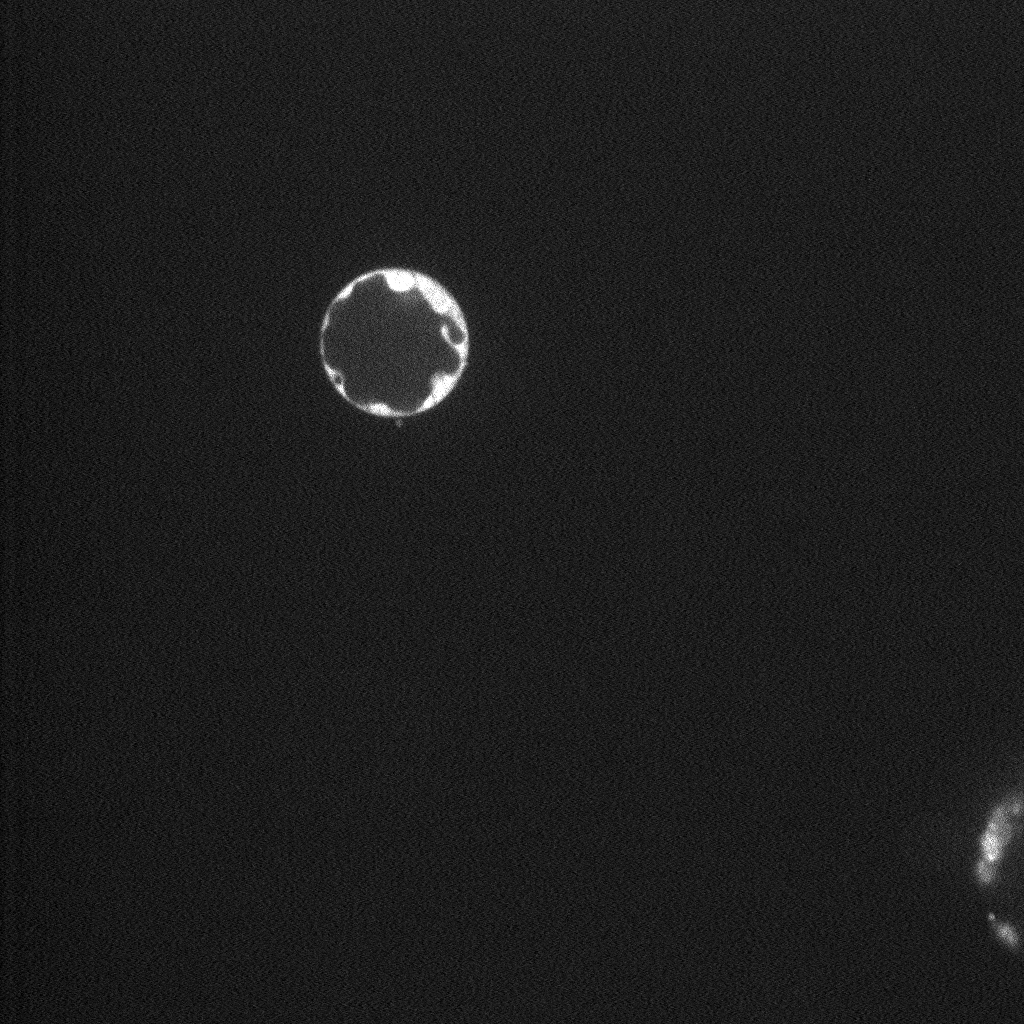

Supplement: koaf217_Supplementary_Data [file koaf217_supplementary_data.zip › Figure 5C source files/Figure 5C source files/Figure 5C-panel 5-ZmPIMT1-NYFP+AtPABP2-CYFP source files/SPYN-ZmPIMT1+SPYC-AtPAB2-9_w0000.tif]

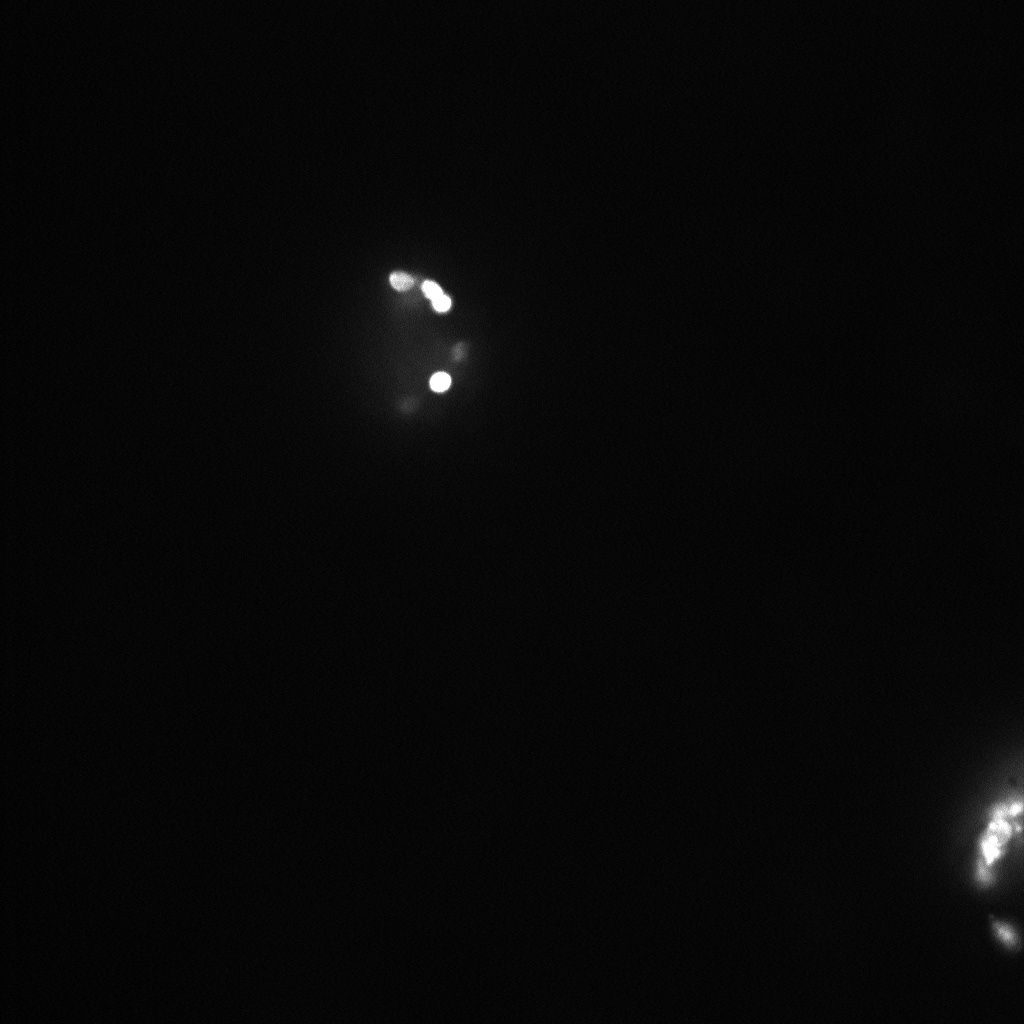

Supplement: koaf217_Supplementary_Data [file koaf217_supplementary_data.zip › Figure 5C source files/Figure 5C source files/Figure 5C-panel 5-ZmPIMT1-NYFP+AtPABP2-CYFP source files/SPYN-ZmPIMT1+SPYC-AtPAB2-9_w0001.tif]

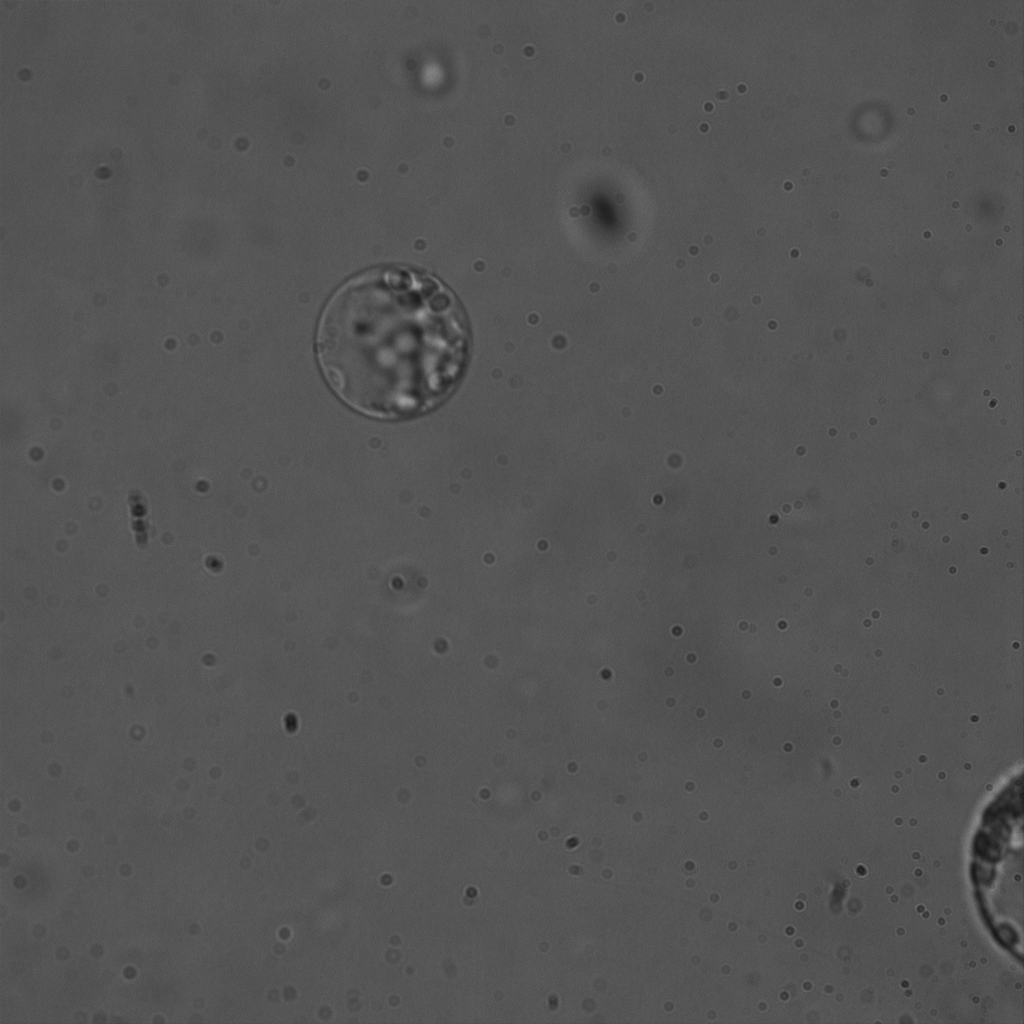

Supplement: koaf217_Supplementary_Data [file koaf217_supplementary_data.zip › Figure 5C source files/Figure 5C source files/Figure 5C-panel 5-ZmPIMT1-NYFP+AtPABP2-CYFP source files/SPYN-ZmPIMT1+SPYC-AtPAB2-9_w0002.tif]

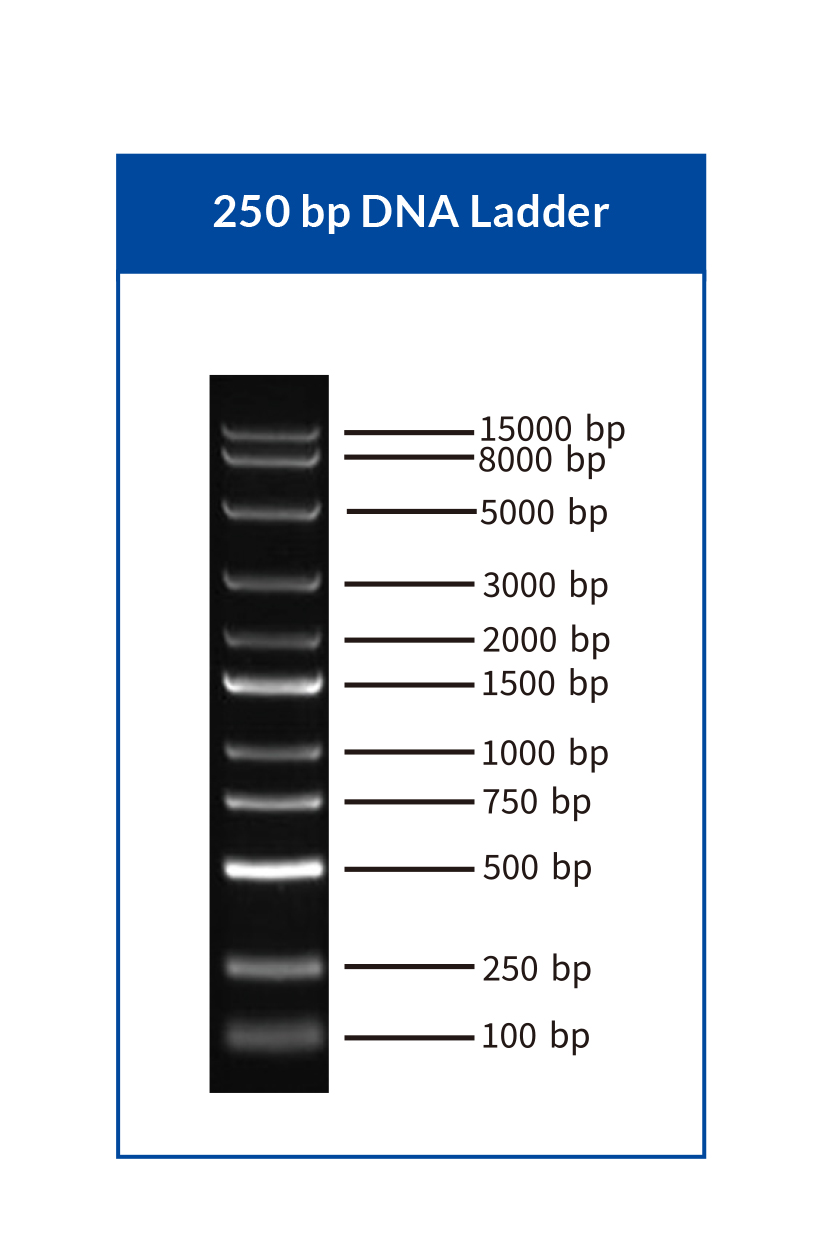

Supplement: koaf217_Supplementary_Data [file koaf217_supplementary_data.zip › Figure S3 source files/Figure S3 source files/DNA ladder.jpg]

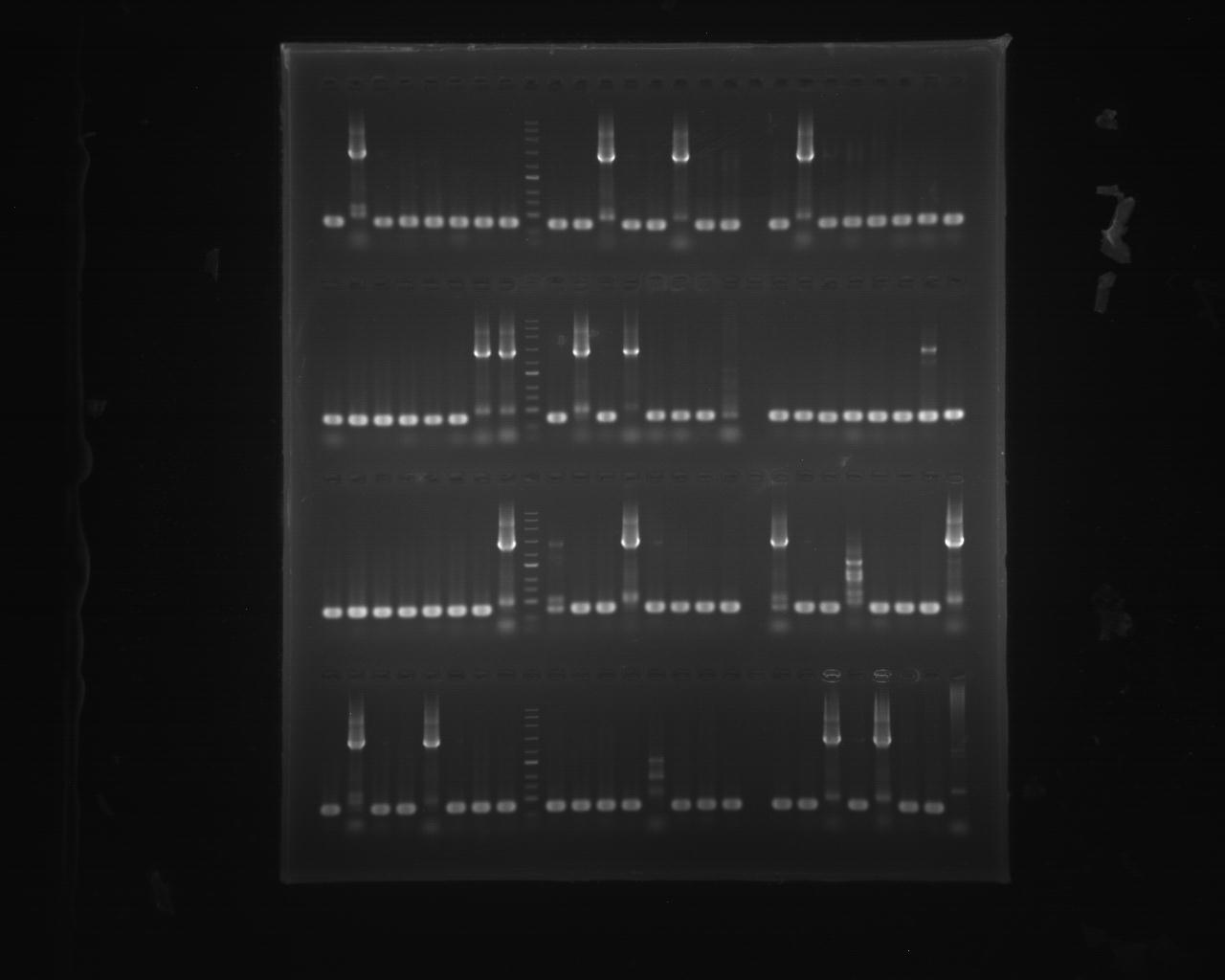

Supplement: koaf217_Supplementary_Data [file koaf217_supplementary_data.zip › Figure S3 source files/Figure S3 source files/Figure S3 panel 1 and 2 sorce image.jpg]

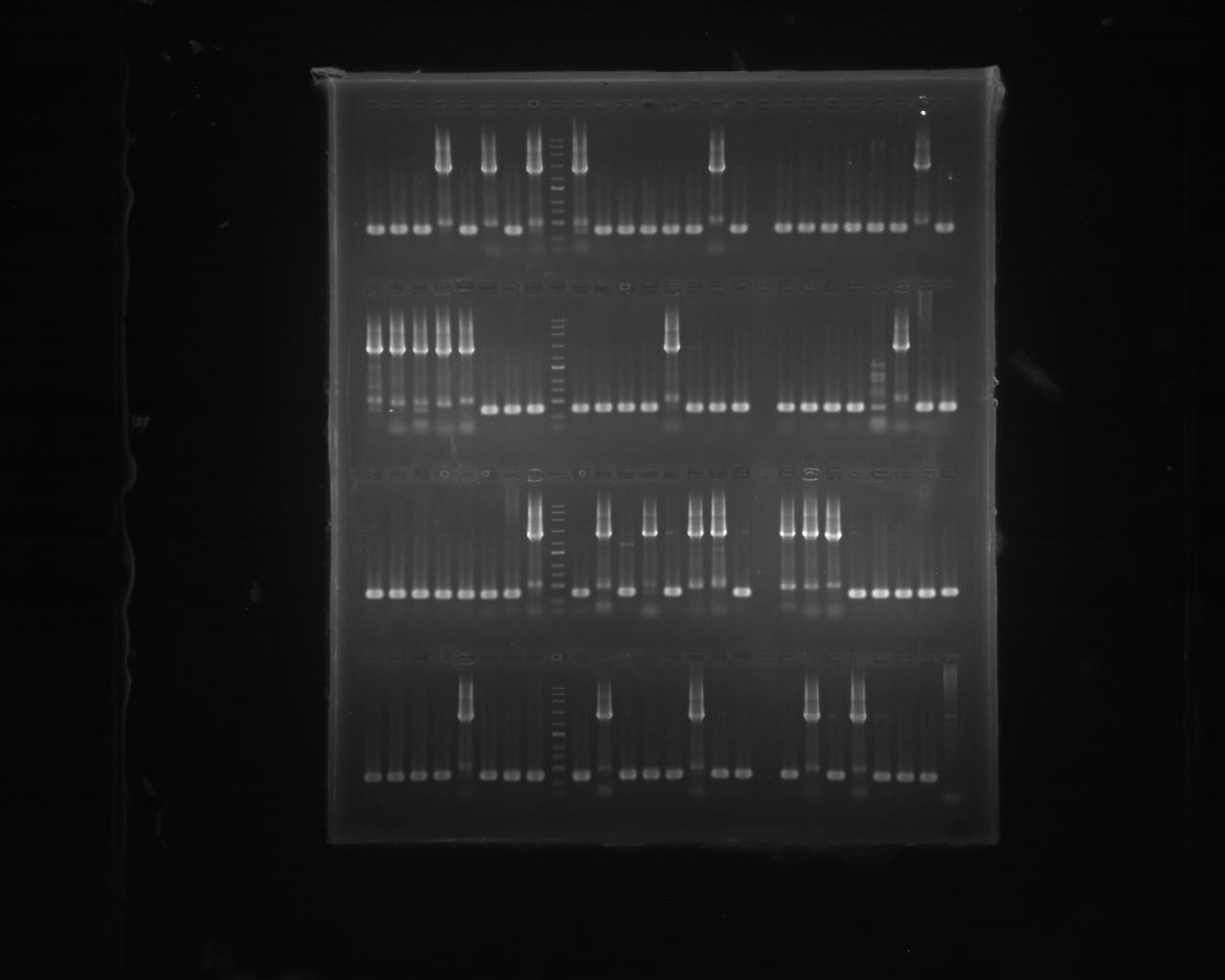

Supplement: koaf217_Supplementary_Data [file koaf217_supplementary_data.zip › Figure S3 source files/Figure S3 source files/Figure S3 panel 3 and 4 sorce image.jpg]

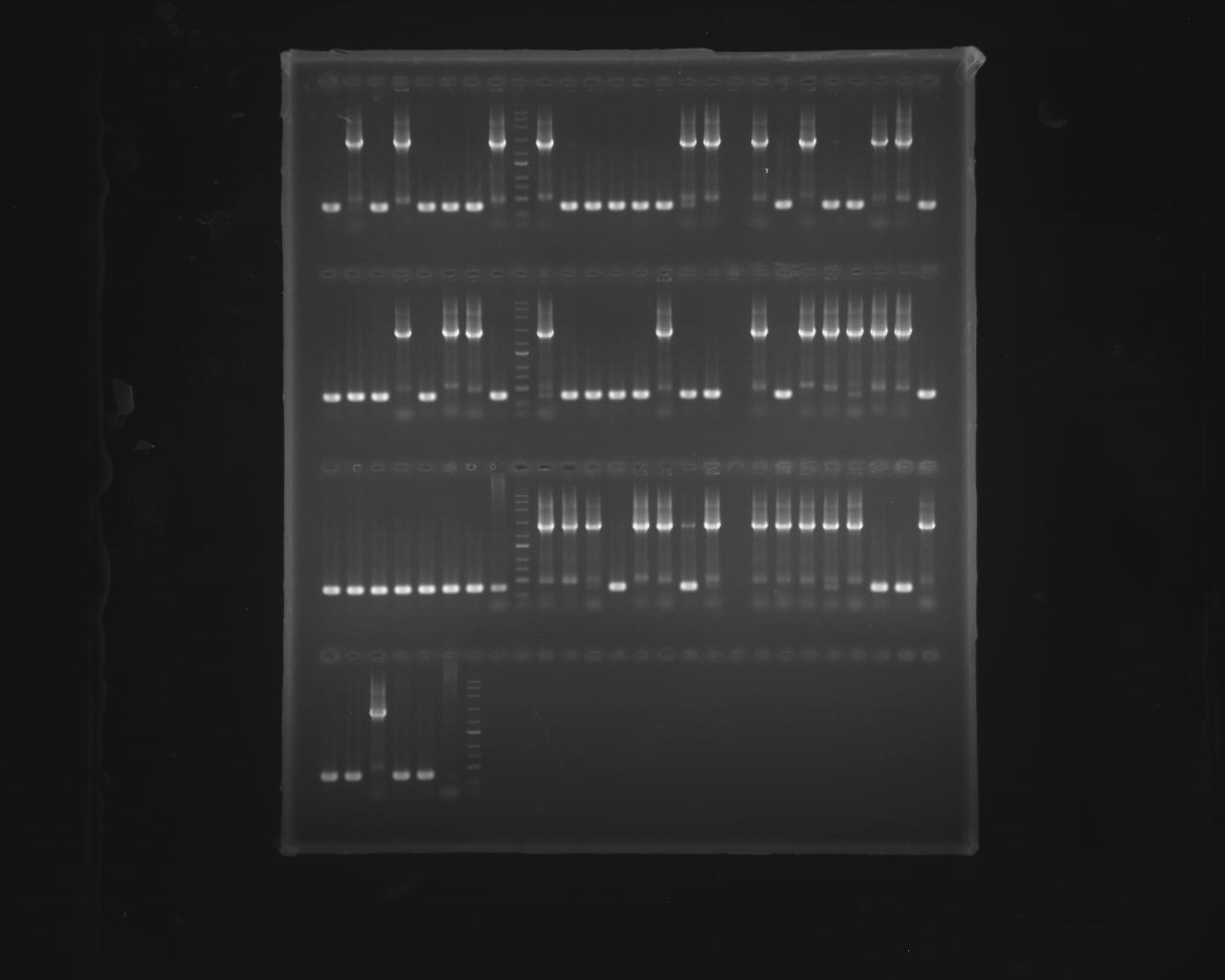

Supplement: koaf217_Supplementary_Data [file koaf217_supplementary_data.zip › Figure S3 source files/Figure S3 source files/Figure S3 panel 5 and 6 sorce image.jpg]

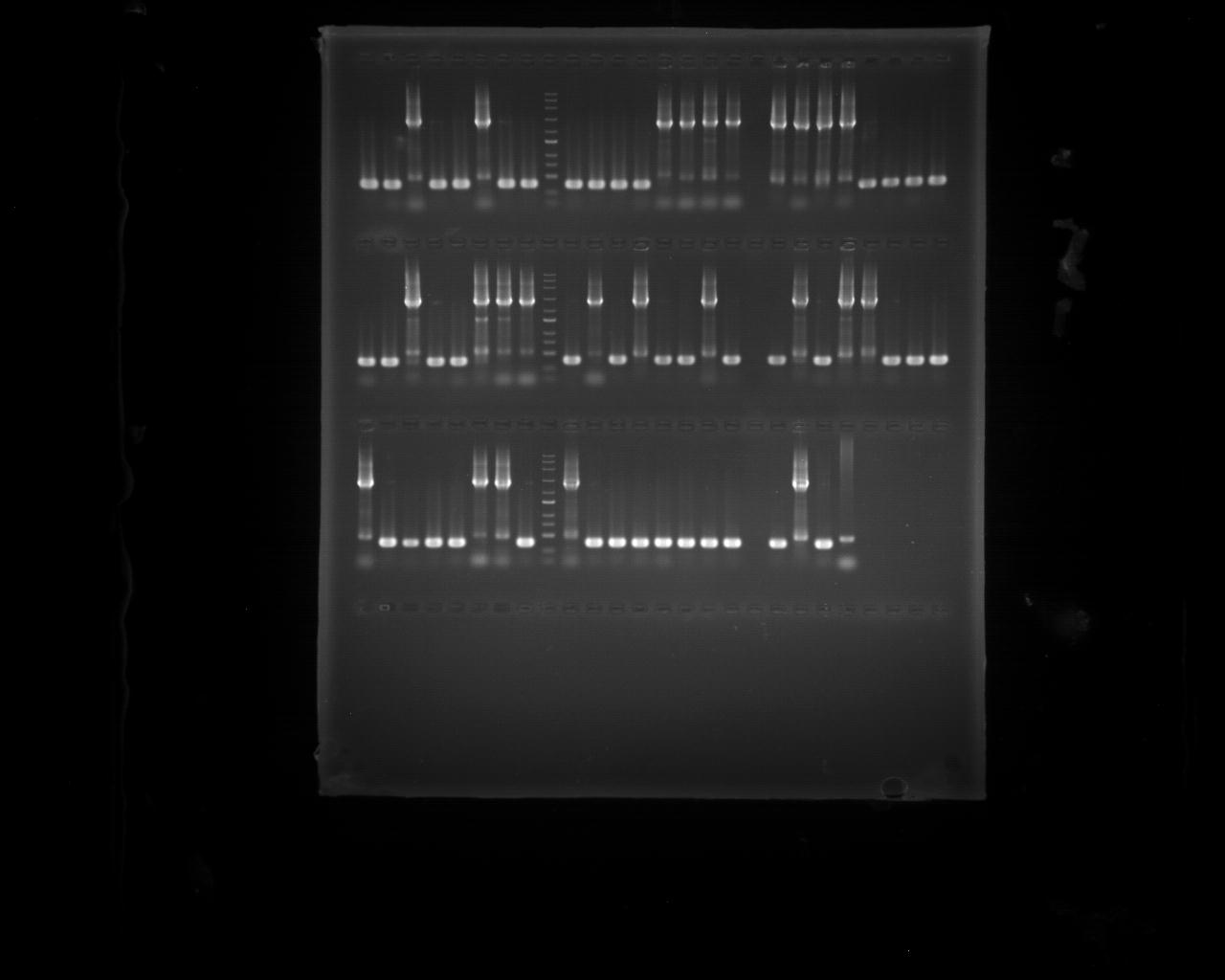

Supplement: koaf217_Supplementary_Data [file koaf217_supplementary_data.zip › Figure S3 source files/Figure S3 source files/Figure S3 panel 6 and 7 sorce image.jpg]

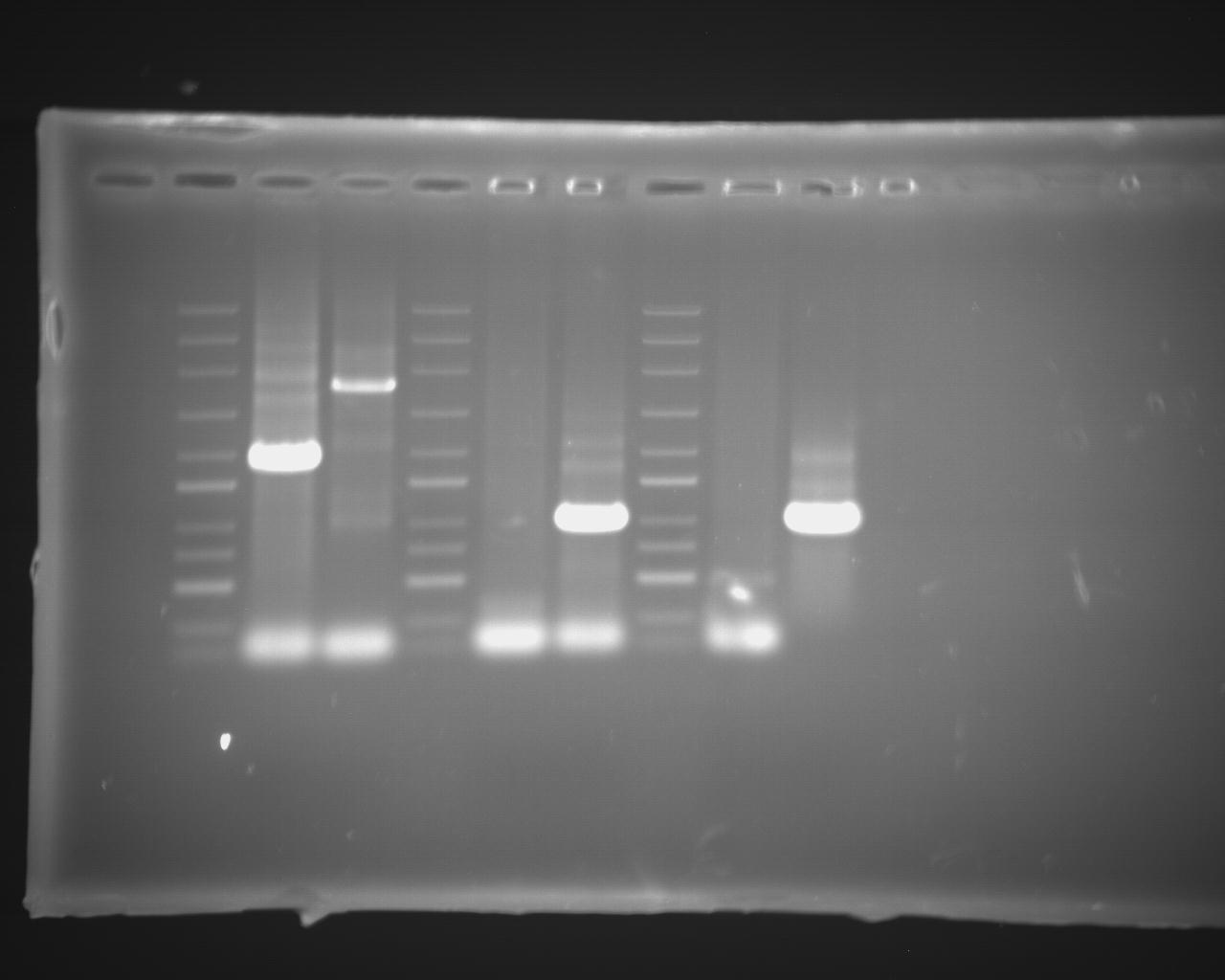


R1+TIR

F1+TIR

F1+R1


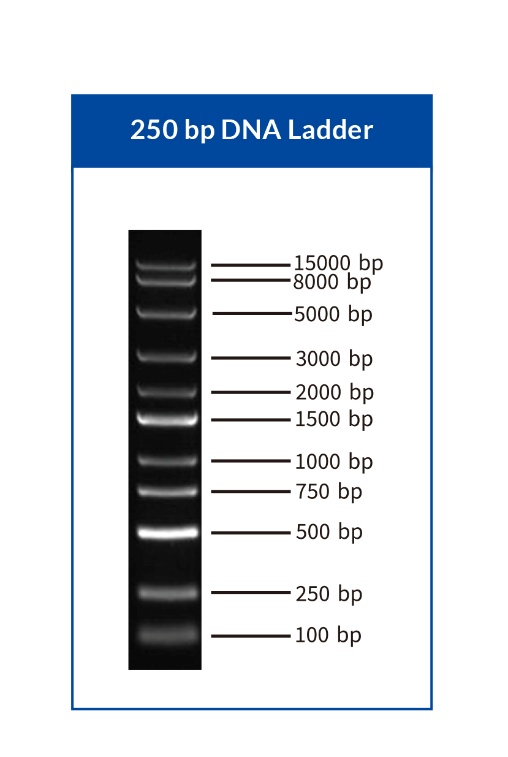

Supplement: koaf217_Supplementary_Data [file koaf217_supplementary_data.zip › Figure S7B source files/Figure S7B source files/Figure S7B source file-description.docx]

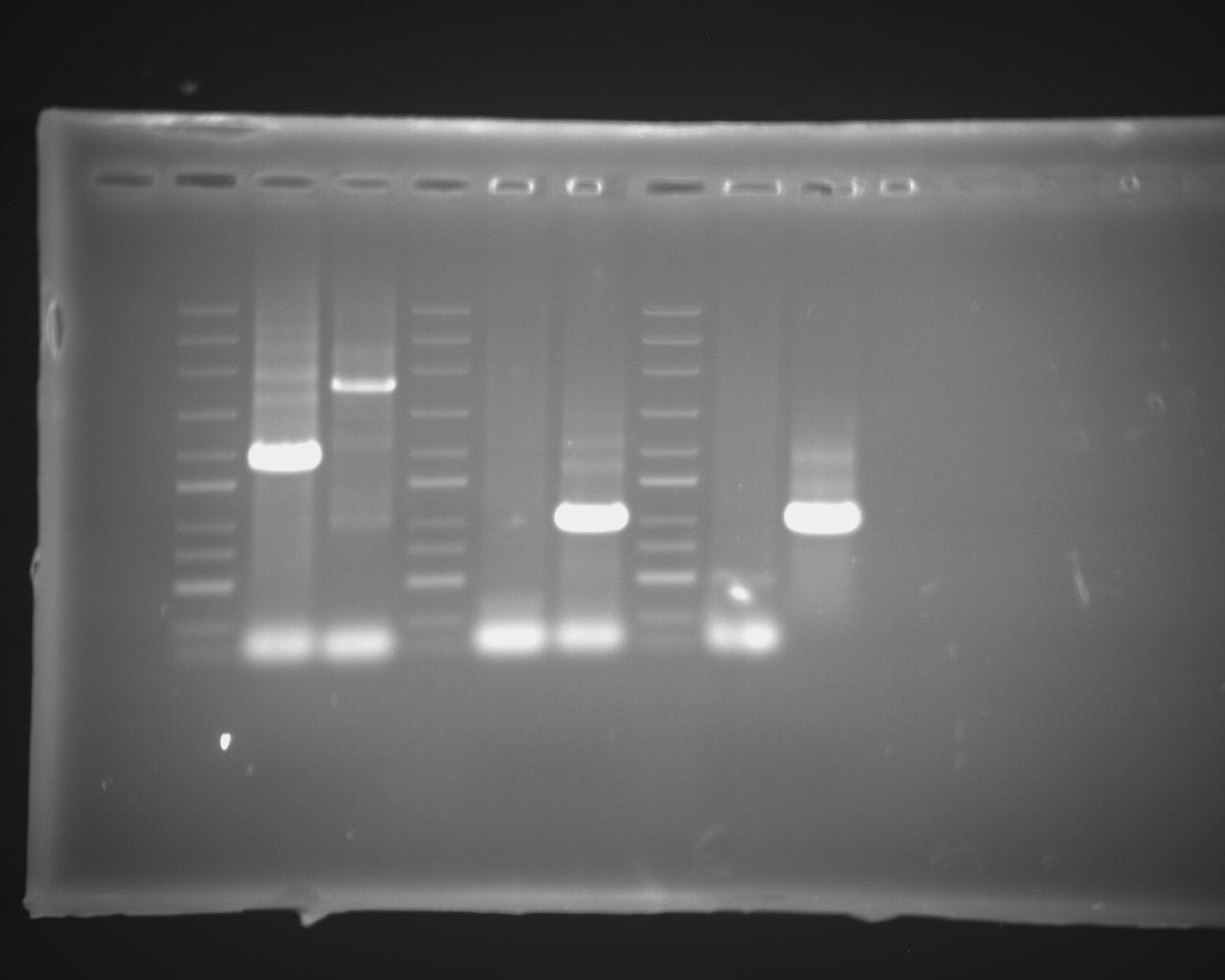

Supplement: koaf217_Supplementary_Data [file koaf217_supplementary_data.zip › Figure S7B source files/Figure S7B source files/Figure S7B source image.jpg]
